# Hydration of $p-$aminobenzoic acid: structures and non-covalent bondings of aminobenzoic acid-water clusters

**DOI:** 10.1007/s00894-023-05810-2

**Published:** 2024-01-12

**Authors:** Diane Anni, Jean Claude Amika Mbema, Alhadji Malloum, Jeanet Conradie

**Affiliations:** 1 Department of Physics, Faculty of Science, University of Maroua, PO BOX 46, Maroua, Cameroon; 2 Department of Chemistry, University of the Free State, PO BOX 339, Bloemfontein, 9300 South Africa; 3 Department of Chemistry, UiT - The Arctic University of Norway, N-9037 Tromsø, Norway

**Keywords:** Aminobenzoic acid, Hydration free energy, Solvation, Non-covalent bondings, QTAIM analysis

## Abstract

****Context**:**

Micro-hydration of the aminobenzoic acid is essential to understand its interaction with surrounding water molecules. Understanding the micro-hydration of the aminobenzoic acid is also essential to study its remediation from wastewater. Therefore, we explored the potential energy surfaces (PESs) of the para-aminobenzoic acid-water clusters, $\text {ABW}_{n}$, $n=1-10$, to study the microsolvation of the aminobenzoic acid in water. In addition, we performed a quantum theory of atoms in molecules (QTAIM) analysis to identify the nature of non-covalent bondings in the aminobenzoic acid-water clusters. Furthermore, temperature effects on the stability of the located isomers have been examined. The located structures have been used to calculate the hydration free energy and the hydration enthalpy of the aminobenzoic acid using the cluster continuum solvation model. The hydration free energy and the hydration enthalpy of the aminobenzoic acid at room temperature are evaluated to be −7.0 kcal/mol and −18.1 kcal/mol, respectively. The hydration enthalpy is in perfect agreement with a previous experimental estimate. Besides, temperature effects on the calculated hydration enthalpy and free energy are reported. Finally, we calculated the gas phase binding energies of the most stable structures of the $\text {ABW}_{n}$ clusters using twelve functionals of density functional theory (DFT), including empirical dispersion. The DFT functionals are benchmarked against the DLPNO-CCSD(T)/CBS. We have found that the three most suitable DFT functionals are classified in the following order: PW6B95D3 > MN15 > $\omega$B97XD. Therefore, the PW6B95D3 functional is recommended for further study of the aminobenzoic acid-water clusters and similar systems.

****Methods**:**

The exploration started with classical molecular dynamics simulations followed by complete optimization at the PW6B95D3/def2-TZVP level of theory. Optimizations are performed using Gaussian 16 suite of codes. QTAIM analysis is performed using the AIMAll program.

**Supplementary Information:**

The online version contains supplementary material available at 10.1007/s00894-023-05810-2.

## Introduction

Para-aminobenzoic acid (also known as $p-$aminobenzoic acid or $4-$aminobenzoic acid) is an organic molecule with two functional groups: carboxyl and amino. Aminobenzoic acid has essential applications in Biochemistry and Chemistry. Furthermore, it is also used in industry for synthesis. Besides, $p-$aminobenzoic acid is a pollutant. Its remediation (or removal) has been the subject of several investigations [1–6]. Therefore, to understand the adsorption process of $p-$aminobenzoic acid, studying its interactions with water molecules surrounding it becomes crucial. Microsolvation of $p-$aminobenzoic acid has received little attention. Thus, in this work, we studied the para-aminobenzoic acid-water clusters. This study is performed in a prelude to investigating the adsorption of $p-$aminobenzoic acid for wastewater treatment. It is important to note that $p-$aminobenzoic acid coexists in water in both the zwitterionic and non-zwitterionic forms. However, the zwitterionic form is more prevalent due to its excellent stability in water. Microsolvation of the zwitterionic and non-zwitterionic forms of $p-$aminobenzoic acid has received negligible consideration. This work focuses on the non-zwitterionic form of $p-$aminobenzoic acid.

Structures of $o-$aminobenzoic acid (or $2-$aminobenzoic acid) water clusters (from monomer to trimer) have been investigated by Ghosh and Chaudhuri [7] at the B3LYP/aug-cc-pVDZ level of theory. For each cluster size, only one stable configuration is reported. The most stable $o-$aminobenzoic acid-water monomer and dimer structure have a cyclic OH$\cdots$O configuration. The most stable structure for the $o-$aminobenzoic acid-water trimer has a double cyclic OH$\cdots$O configuration [7]. In addition to the structures, NMR spin-spin couplings of the investigated structures have also been reported. Besides, microhydration of $o-$aminobenzoic acid in anionic protonated form has been studied by da Silva Olivier and coworkers [8]. Microhydration has been studied from one to five explicit water molecules at the B3LYP/TZVP level of theory in implicit solvent (using the polarizable continuum model, PCM). The study has examined the effects of the water molecules on the UV–Vis spectrum of $o-$aminobenzoic acid in the anionic protonated form [8]. Due to the anionic form, the structures reported by da Silva Olivier and coworkers [8] are different from those obtained by Ghosh and Chaudhuri [7]. It has been found that the maximum absorption wavelength increases with the number of explicit water molecules [8]. Rosbottom et al. [9] have studied the interactions of $p-$aminobenzoic acid with solvent molecules of water, ethanol, and acetonitrile. They started with molecular dynamics simulations and optimized the structures at the B3LYP/6-31 G(d) level of theory. They identified possible fixation sites of water, ethanol, and acetonitrile molecules on the $p-$aminobenzoic acid. They found that the water molecules prefer to be attached to the carboxyl group of $p-$aminobenzoic acid [9].

In addition to the structures of aminobenzoic acid-water clusters, solvation enthalpy and solvation free energy of the aminobenzoic acid has been reported by a few authors [10–13]. Turner et al. [11] have reported the solvation enthalpy of the $p-$aminobenzoic acid in the water, in ethanol, and in acetonitrile using an experimental approach. They have also calculated the hydration free energy of the $p-$aminobenzoic acid in the solvents mentioned above using molecular dynamics simulations. The same authors have reported dissolution enthalpy and dissolution free energies of the $p-$aminobenzoic acid [11]. Recently, Li et al. [13] have evaluated several thermodynamic properties of the $p-$aminobenzoic acid using experimental approaches and molecular dynamics simulations. Using molecular dynamics simulations, the authors calculated the hydration free energy of the $p-$aminobenzoic acid in several solvents: methyl acetate, n-propyl acetate, isopropyl acetate, acetone, and water [13].

An exploration of the literature shows that very few studies of the $p-$aminobenzoic acid-water clusters have been reported. Furthermore, the authors must thoroughly explore the possible structures even for the reported studies. Thus, we explored the potential energy surfaces (PESs) of $p-$aminobenzoic acid-water clusters, starting with classical molecular dynamics simulations followed by full optimizations at the PW6B95D3/def2-TZVP level of theory. Quantum theory of atoms in molecules (QTAIM) analysis has been performed to understand the nature of non-covalent interactions. The generated structures have been used to calculate the hydration enthalpy and the hydration free energy of the $p-$aminobenzoic acid at different temperatures.

## Methodology

We start this section by presenting the cluster continuum solvation model used in this work to compute the solvation free energy and the solvation enthalpy of aminobenzoic acid (see the “Solvation free energy and enthalpy” section). Then, the methodology used to sample initial configurations is presented (see the “Geometry sampling” section). Finally, we present the computational details, including the choice of the computational level of theory, the software used, and details to enhance the accuracy of the calculations (see the “Computational details” section).

**Fig. 1:**
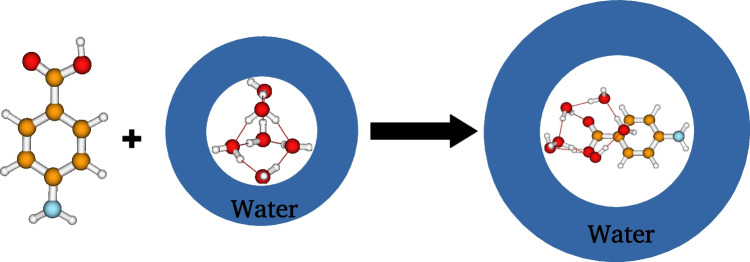
Schematic representation of the cluster continuum solvation model used in this work to calculate the solvation free energy and enthalpy of aminobenzoic acid in water. The representation is given for the case of six explicit water molecules

### Solvation free energy and enthalpy

The aminobenzoic acid’s solvation free energy and the solvation enthalpy are calculated using the cluster continuum solvation model. A schematic representation of the cluster continuum solvation model is given in Fig. 1, which expresses the Eq. 1. The main idea of the cluster continuum solvation model is to adopt a hybrid solvation model. The solvent molecules closer to the solute (aminobenzoic acid) are treated explicitly using quantum mechanics, while solvent molecules far from the solute are considered a continuum medium. The advantage of the model is that only a few explicit water molecules are required to achieve convergence. Therefore, the model will allow a considerable saving of computational time. The cluster continuum solvation model has been successfully applied in the literature to compute the solvation free energy of the proton in solutions [14–23]. Recently, we applied the cluster continuum solvation model to calculate the solvation free energy and enthalpy of phenol in water at different temperatures [24].

1 $$\begin{aligned} \text {AB}(g)+(\textrm{H}_{2}\textrm{O})_{n}(s)\longrightarrow \text {AB}(\textrm{H}_{2}\textrm{O})_{n}(s) \end{aligned}$$

The solvation free energy and the solvation enthalpy of aminobenzoic acid within the cluster continuum solvation model can be calculated using Eqs. 2 and 3, respectively.

2 $$\begin{aligned} \Delta G_{s}(\text {AB})_{n}= &   \Delta G_{s}[\text {AB}(\text {H}_{2}\text {O})_{n}]-\Delta G_{s}[(\text {H}_{2}\text {O})_{n}]\nonumber \\  &   -\Delta G_{g}[\text {AB}], \end{aligned}$$

3 $$\begin{aligned} \Delta H_{s}(\text {AB})_{n}= &   \Delta H_{s}[\text {AB}(\text {H}_{2}\text {O})_{n}]-\Delta H_{s}[(\text {H}_{2}\text {O})_{n}]\nonumber \\  &   -\Delta H_{g}[\text {AB}], \end{aligned}$$

where AB stands for aminobenzoic acid. The superscript *s* and *g* are solvent and gas phases, respectively. $\Delta G_{s}[X]$ and $\Delta H_{s}[X]$ represent X’s free energy and enthalpy in the solvent phase, respectively. Similar meanings for $\Delta G_{g}[X]$ and $\Delta H_{g}[X]$.

Examination of Eqs. 2 and 3 shows that the calculation of the solvation free energy and enthalpy of aminobenzoic acid is subjected to the determination of the structures of $\text {AB}(\text {H}_{2}\text {O})_{n}$ as well as the structures of $(\text {H}_{2}\text {O})_{n}$ for different values of *n*. The structures of $\text {AB}(\text {H}_{2}\text {O})_{n}$ are thoroughly explored in this work. The free energy and the enthalpy of $\text {AB}(\text {H}_{2}\text {O})_{n}$, as required in Eqs. 2 and 3, are calculated as Boltzmann average over the free energy and enthalpy of all possible configurations of the cluster. The structures of neutral water clusters, $(\text {H}_{2}\text {O})_{n}$, have been thoroughly explored in our previous works [25, 26] using ABCluster as described in the “Geometry sampling” section. However, only the most stable structures in our previous works have been re-optimized at the PW6B95D3/def2-TZVP to compute the solvation free energy and enthalpy of aminobenzoic acid in this work. This has been done for tractability and could slightly affect the calculated hydration energies. Consequently, one needs to determine the structures of $\text {AB}(\text {H}_{2}\text {O})_{n}$ for different values of *n* to be able to use Eqs. 2 and 3. In this work, we have determined different structures of $\text {AB}(\text {H}_{2}\text {O})_{n}$ for $n=1$ to $n=10$. We started this work by sampling different possible configurations for each value of *n*. The sampling has been performed using classical molecular dynamics as implemented in the ABCluster (see the “Geometry sampling” section).

### Geometry sampling

Initial configurations have been sampled using the ABCluster code of Zhang and Dolg [27, 28]. ABCluster samples all possible configurations on a given molecular cluster’s potential energy surface (PES). The sampling is performed using classical molecular dynamics with potential energy constituted of electrostatic and Lenard-Jones interactions. The potential energy parameters are retrieved from the CHARMM force field [29]. The sampling details using ABCluster can be found in our previous works on molecular clusters [30–34]. Moreover, more details on the artificial bee colony algorithm (global optimization algorithm used in ABCluster) can be found in the initial papers of Zhang and Dolg [27, 28]. The located configurations of different cluster sizes have been fully optimized at the PW6B95D3/def2-TZVP level of theory (see the “Computational details” section for more details).

### Computational details

The configurations located using ABCluster are fully optimized using the PW6B95D3 DFT functional. Due to non-covalent bondings that stabilize the aminobenzoic acid-water clusters, we had to consider the dispersive nature of the interactions. This dispersive nature justifies the choice of a functional including the third order Grimme’s dispersion corrections [35] (the PW6B95D3 functional). In addition, the PW6B95D3 functional is the most accurate for studying clusters with non-covalent interactions in our previous works [36–39]. Two different basis sets have been tested to optimize the configurations: cc-pVDZ and def2-TZVP. For the accuracy of the energies, the def2-TZVP (a triple zeta basis set) will be considered in the calculations of the solvation free energy and the solvation enthalpy. Frequency calculations have been systematically performed along with all optimizations. The frequency calculations have been performed to confirm the location of true local minima on the PESs and to calculate the free energy and enthalpy of the corresponding structure. Optimizations and frequency calculations have been performed using the Gaussian 16 suite of codes [40]. The *tight* option has been used for accurate optimization, and the *ultrafine* grid has been used for accurate integrals calculations. Optimizations and frequency calculations have been performed in the implicit solvation model. The solvation model based on density (SMD) has been used for the implicit solvation [41]. Thus, the structures of $\text {AB}(\text {H}_{2}\text {O})_{n}$ as well as $(\text {H}_{2}\text {O})_{n}$ for different values of *n* have been calculated in the implicit solvation model.

The gas phase binding energies of the most stable isomers have been calculated using twelve DFT functionals, including Grimme’s empirical dispersion [35, 42]. The functionals include B3LYP-D3 [43], B3PW91-D3 [43], M05-D3 [44], M052X-D3 [45], M06-D3 [46], M062X-D3 [46], MN15 [47], PBE1PBE-D3 [48], PBEPBE-D3 [49], PW6B95D3 [50], TPSSTPSS-D3 [51], and $\omega$B97XD [52]. The most stable structures located at the PW6B95D3/def2-TZVP level of theory have been fully re-optimized using the above functionals associated with the def2-TZVP basis set. Gaussian 16 suite of codes [40] has been used for these calculations along with *tight* and *ultrafine* options as described above. In addition, binding energies are also calculated at the DLPNO-CCSD(T)/CBS level of theory to serve as a benchmark for DFT functionals. Calculations at the DLPNO-CCSD(T)/CBS level of theory have been performed using the Orca program [53]. We used *tightpno* and *tightscf* for accuracy. In addition, we used the *AutoAux* option for the automatic generation of auxiliary basis sets [54]. The CBS extrapolation has been performed using the two-point strategy involving electronic energies calculated using the def2-TZVPP and the def2-QZVPP basis sets. Further details on the CBS extrapolation can be found in our previous works [38, 39].

We performed a quantum theory of atoms in molecules (QTAIM) analysis on the most stable structures to understand the nature of non-covalent bonding in the aminobenzoic acid-water clusters. The QTAIM analysis uses the AIMAll code [55]. The QTAIM analysis has been performed only on the most stable configurations obtained at the PW6B95D3/def2-TZVP level of theory. Regarding the relative population, the program TEMPO [56, 57] has been used to compute the relative population of the clusters at different temperatures.

## Results and discussions

In this section, we start by presenting the structures of the aminobenzoic acid-water clusters as optimized at the PW6B95D3/def2-TZVP level of theory. The structures are presented along with their solvent phase relative electronic energies, including zero point energy (ZPE) corrections (see the “Structures and relative energies” section). After presenting the stability, we examine the nature of non-covalent bondings stabilizing the studied clusters in the “Non-covalent bondings in $\text {ABW}_{n}$ structures” section. Next, we presented the relative population of the clusters to highlight the structures that significantly contribute to the cluster’s population (see the “Relative population of $\text {ABW}_{n}$ structures” secton). Then, the structures, as well as their free energies and enthalpies, are used to evaluate the absolute hydration free energy and the absolute hydration enthalpy of the aminobenzoic acid for different ranges of temperature (see the “Solvation free energy and solvation enthalpy” section). Finally, we present the binding energies calculated using twelve DFT functionals benchmarked against DLPNO-CCSD(T). These binding energies are calculated to select the most suitable functional for studying the interaction between the aminobenzoic acid and water molecules (see the “Gas phase binding energies and DFT benchmarking” section).

**Fig. 2:**
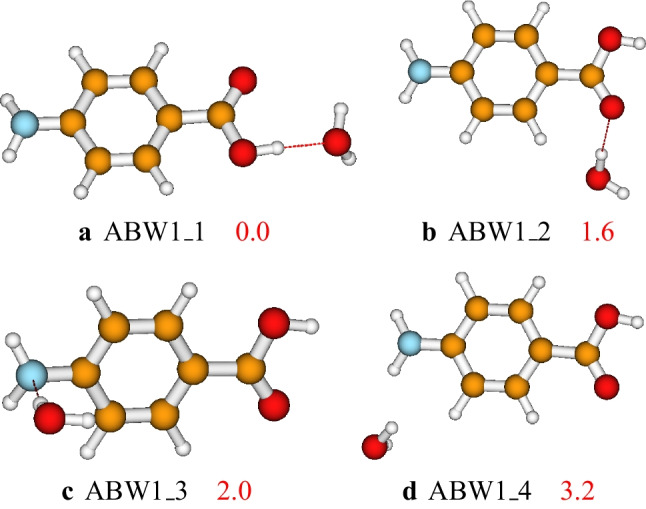
Structures and relative energies of $\text {ABW}_{1}$ as optimized at the PW6B95D3/def2-TZVP level of theory. The relative energies are reported in kcal/mol

**Fig. 3:**
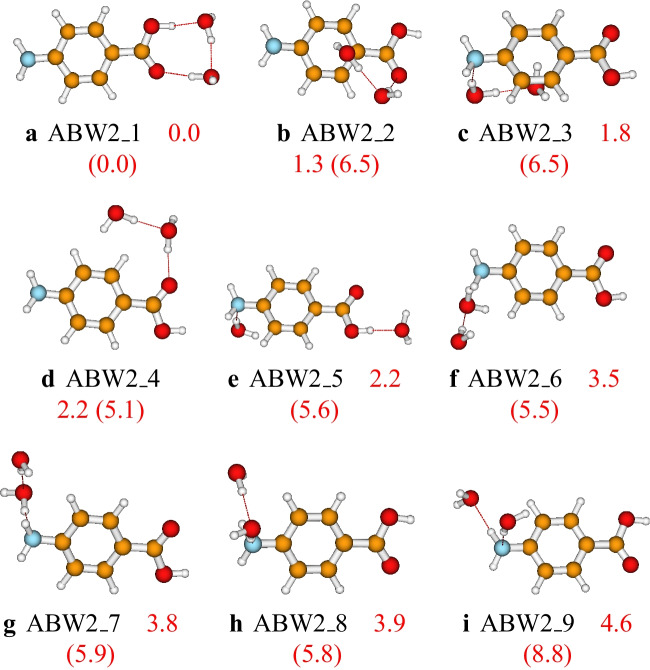
Structures and relative energies of $\text {ABW}_{2}$ as optimized at the PW6B95D3/def2-TZVP level of theory. Numbers are the calculated relative energies (in kcal/mol). Numbers in parenthesis are relative energies calculated at the PW6B95D3/cc-pVDZ level of theory

### Structures and relative energies

After complete optimization at the PW6B95D3/def2-TZVP level of theory, the configurations found different from one another have been retained. Four different configurations of the $\text {ABW}_{1}$ have been located on its PES within the ZPE-corrected electronic energy landscape of 3.2 kcal/mol. The located structures are reported in Fig. 2. In Fig. 2, the global minimum energy structure is **ABW1_1**. The second most stable isomer of the aminobenzoic acid-water monomer lies 1.6 kcal/mol, **ABW1_2**. In **ABW1_1**, the water monomer is a proton acceptor, while in **ABW1_2**, **ABW1_3**, and **ABW1_4**, the water monomer is a proton donor. In addition, we note that when the water molecule interacts with the amino- group, the generated isomer is less stable than that generated when the water molecule interacts with the carboxyl group (see Fig. 2). For $\text {ABW}_{1}$, the four structures have been optimized at the same level of theory using the CPCM and the PCM solvation models. The calculated relative energies are reported in Fig. S1 of the supporting information. It has been found that the structure’s geometry does not considerably change with the solvation model. In addition, all three solvation models (CPCM, PCM, and SMD) predicted the same structure as the most stable. However, the relative energy of the isomers **ABW1_3** and **ABW1_4** are exchanged using CPCM and PCM (see details in the supporting information).

To assess the influence of the computational level of theory on the geometry of the clusters, we optimized all the structures of $\text {ABW}_{1}$, $\text {ABW}_{2}$, and $\text {ABW}_{3}$ at the MP2/def2-TZVP level of theory. It has been found that most of the geometries obtained at the MP2 are identical to those obtained at the PW6B95D3. However, there are a few geometries where the water molecules exhibit a slight shift that does not visually change the geometries. Regarding their energies, it has been found that the relative energies at these two levels of theory follow different trends. However, for each cluster size, the most stable and the least stable structures are predicted to be the same at MP2 and PW6B95D3 levels of theory (see Figs. S2, S3, and S4 of the supporting information).

For the aminobenzoic acid-(water)_2_, nine different isomers are located on the cluster’s PES (see Fig. 3). The most stable configuration, **ABW2_1**, has three OH$\cdots$O hydrogen bondings forming a cyclic configuration. The stability of cyclic configurations for small-sized clusters perfectly agrees with the study of neutral water clusters [26, 58]. Generally, the isomers where the water molecules are attached to the carboxyl group are among the most stable. In contrast, the isomers where the water molecules are attached to the amino group have lesser stability (see Fig. 3). This stability trend is because the carboxyl group is more hydrophilic than the amino group. Consequently, water molecules establish more robust bondings with the carboxyl group than the amino group. This stability trend is also noted in larger-sized aminobenzoic acid-water clusters studied in this work. The relative energies calculated using the two basis sets (cc-pVDZ and def2-TZVP) follow almost the same stability trend (see Fig. 3). Both basis sets predicted the same global minimum energy structure and the same least stable structure. The relative energies calculated using the cc-pVDZ basis set are larger than those calculated using the def2-TZVP basis set. This difference highlights the overestimation of the energies calculated using the cc-pVDZ basis set.

**Fig. 4:**
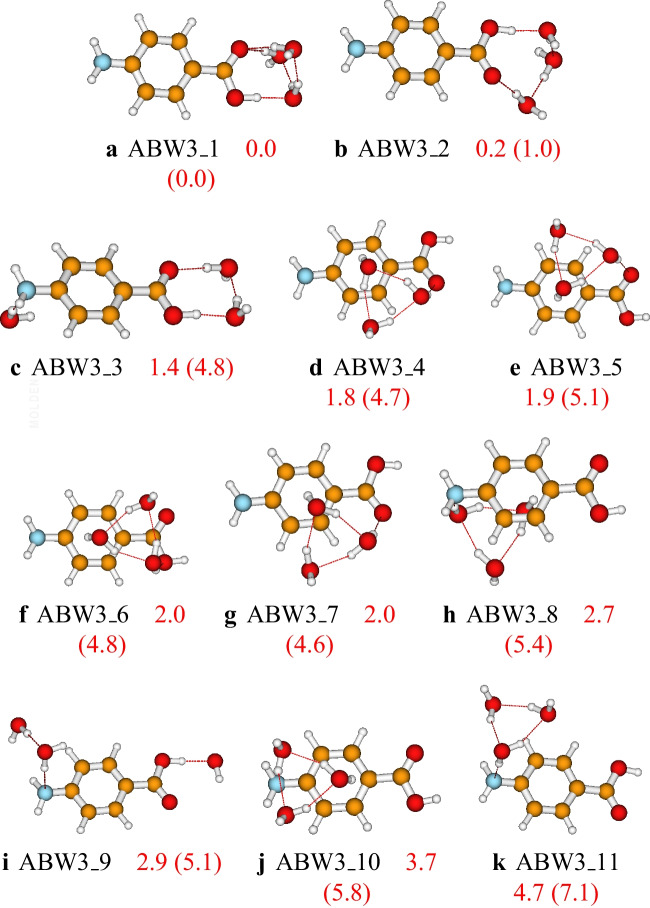
Structures and relative energies of $\text {ABW}_{3}$ as optimized at the PW6B95D3/def2-TZVP level of theory. Numbers are the calculated relative energies (in kcal/mol). Numbers in parenthesis are relative energies calculated at the PW6B95D3/cc-pVDZ level of theory

**Fig. 5:**
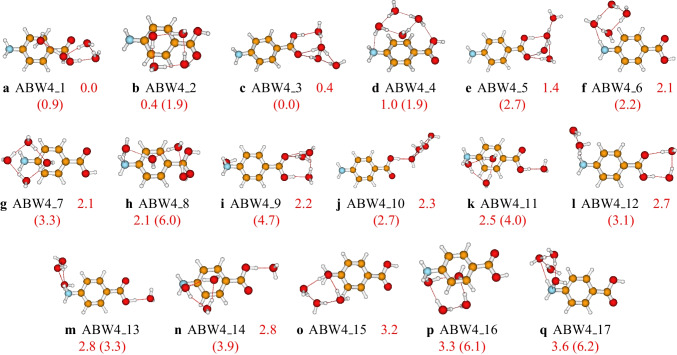
Structures and relative energies of $\text {ABW}_{4}$ as optimized at the PW6B95D3/def2-TZVP level of theory. Numbers are the calculated relative energies (in kcal/mol). Numbers in parenthesis are relative energies calculated at the PW6B95D3/cc-pVDZ level of theory

**Fig. 6:**
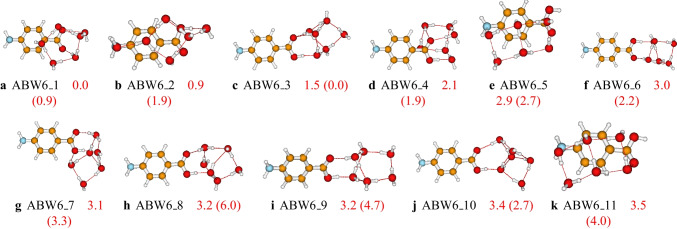
Structures and relative energies of $\text {ABW}_{6}$ as optimized at the PW6B95D3/def2-TZVP level of theory. Numbers are the calculated relative energies (in kcal/mol). Numbers in parenthesis are relative energies calculated at the PW6B95D3/cc-pVDZ level of theory. Only the eleven first isomers are reported here. The complete list of the located structures is provided in the supporting information

Eleven configurations of the aminobenzoic acid-water trimer have been identified on the PES of the cluster at the PW6B95D3/def2-TZVP level of theory. The located isomers and their relative energies are reported in Fig. 4. The predicted most stable isomer of the $\text {ABW}_{3}$ cluster, **ABW3_1**, has a pyramidal configuration of the water molecules and the COOH group. The second most stable isomer, **ABW3_2**, exhibits a folded cyclic configuration. In most of the structures of the $\text {ABW}_{3}$ cluster, the three water molecules form a cyclic configuration interacting with the aminobenzoic acid (see **ABW3_4** to **ABW3_8** in Fig. 4). Similar to the case of $\text {ABW}_{1}$ and $\text {ABW}_{2}$, when the water molecules interact with the carboxyl group, the generated structure is found to be more stable than the structure generated by the interaction with the amino group. Regarding the effect of the basis sets, we noted that both cc-pVDZ and def2-TZVP predicted the same global minimum energy structure. In addition, the stability trend is the same for both basis sets (see Fig. 4).

**Fig. 7:**
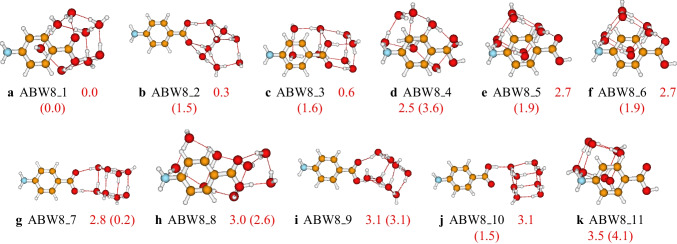
Structures and relative energies of $\text {ABW}_{8}$ as optimized at the PW6B95D3/def2-TZVP level of theory. Numbers are the calculated relative energies (in kcal/mol). Numbers in parenthesis are relative energies calculated at the PW6B95D3/cc-pVDZ level of theory. Only the eleven first isomers are reported here. The complete list of the located structures is provided in the supporting information

**Fig. 8:**
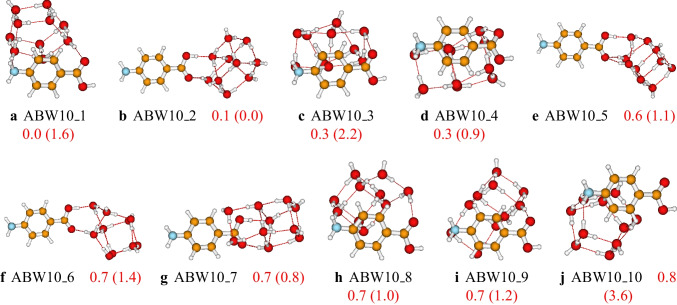
Structures and relative energies of $\text {ABW}_{10}$ as optimized at the PW6B95D3/def2-TZVP level of theory. Numbers are the calculated relative energies (in kcal/mol). Numbers in parenthesis are relative energies calculated at the PW6B95D3/cc-pVDZ level of theory. Only the ten first isomers are reported here. The complete list of the located structures is provided in the supporting information

We located seventeen structures on the PES of $\text {ABW}_{4}$ cluster, reported in Fig. 5. In the most stable structure, **ABW4_1**, the water molecules form a chain interacting with the aminobenzoic acid. As seen in the “Non-covalent bondings in $\text {ABW}_{n}$ structures” section, **ABW4_1** is stabilized by strong OH$\cdots$O hydrogen bondings and OH$\cdots \pi$ bonding interactions. **ABW4_1** has five OH$\cdots$O and two OH$\cdots \pi$ bonding interactions. There are two degenerate second most stable structures, **ABW4_2** and **ABW4_3**, lying 0.4 kcal/mol above the most stable configuration (see Fig. 5). The structures in which the water molecules interact with the carboxyl group are the most stable, while those in which the water molecules interact with the amino group are found to be the least stable. There are structures between these two groups in which the water molecules interact with the carboxyl and amino groups.

The structures of larger clusters of $\text {ABW}_{n}$ ($n=6-10$) are reported in Figs. 6, 7, and 8, respectively. Generally, the most stable configurations are the structures where the water molecules establish strong OH$\cdots$O hydrogen bondings with the carboxyl group. However, for the case of $\text {ABW}_{10}$ cluster, water molecules interact with both carboxyl and amino groups in the most stable configuration (see Fig. 8). This is due to the relatively large number of water molecules compared to smaller-sized clusters. It has been noted that the water molecules occupy one side of the aminobenzoic acid in all the studied clusters. This behavior is mainly attributed to the clusters’ stability and the number of explicit water molecules. In this work, we have limited the study to a maximum of ten explicit water molecules. With ten water molecules, if the water molecules were shared on both sides of the aminobenzoic acid, the resulting structure would be less stable than those reported in this work. Therefore, the water molecules prefer to be on one side for these small-sized clusters to enhance stability. Furthermore, even the initial structures generated in the gas phase by the ABCluster have similar behavior. Regarding the basis set effects on larger clusters, it is generally found that the predicted stability trend using these two basis sets (def2-TZVP and cc-pVDZ) is different. This difference could indicate that the cc-pVDZ basis set is not large enough to achieve accuracy. On the other hand, one could also expect that a basis set larger than def2-TZVP could be necessary. However, Grimme and coworkers [59] suggested that a basis set larger than a triple zeta is generally excessive for the description of structures and frequencies.

**Fig. 9:**
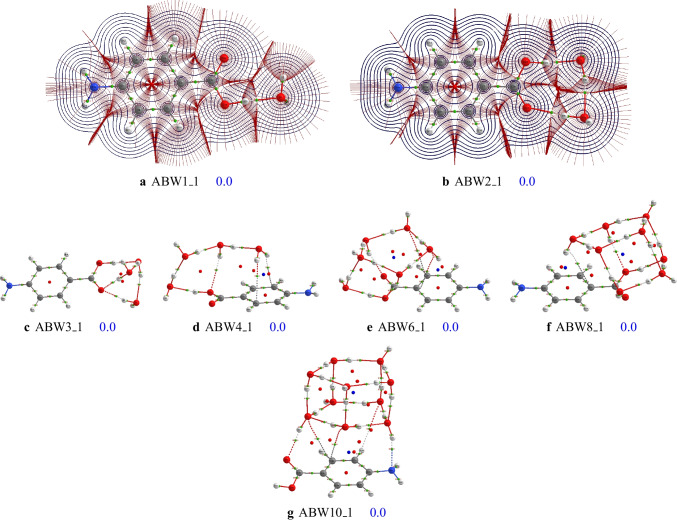
Critical points and bond paths of most stable configurations of the studied $\text {ABW}_{n}$. For the $\text {ABW}_{1}$ and $\text {ABW}_{2}$, the atomic basins, as well as the 2D contour map of the electron density, are represented

### Non-covalent bondings in $\text {ABW}_{n}$ structures

Understanding the nature of the interactions that link the water molecules to the aminobenzoic acid is essential to understanding its micro solvation in water. This is also important to understand the stability of the generated clusters. Therefore, QTAIM analysis has been performed on the most stable configurations of $\text {ABW}_{n}$ based on their structures and their electron densities calculated at the PW6B95D3/def2-TZVP level of theory. Bond paths, bond critical points, and their properties are calculated using the AIMAll program [55]. The most stable configurations’ calculated bond paths and critical points are reported Fig. 9. For **ABW1_1** and **ABW2_1**, we have also represented the 2D contour map of the electron density in the plane containing the phenyl group. Atomic basins of some atoms in the plane have also been represented. In order to determine the nature of non-covalent bondings, properties of all bond critical points are reported in the supporting information. These properties include the electron density, $\rho$, the Laplacian of the electron density, $\nabla ^{2}\rho$, the ellipticity, the kinetic energy, and the difference between the bond path length and the geometrical bond length.

The most stable structure of $\text {ABW}_{1}$ exhibits a strong OH$\cdots$O hydrogen bonding, where the water molecule acts as a proton acceptor. At the bond critical point of OH$\cdots$O, the electron density is evaluated to be ${0.0443}{\text {ea}}_{0}^{-3}$, while $\nabla ^{2} \rho$ is ${1157}{\text {ea}}_{0}^{-5}$, highlighting a strong hydrogen bonding. Similarly, the **ABW2_1** and **ABW3_1** are stabilized by strong hydrogen bondings. Non-covalent bonding different from OH$\cdots$O appears in **ABW4_1** (the reader is reminded that this analysis applies only to the most stable configurations). **ABW4_1** has five OH$\cdots$O hydrogen bondings and one OH$\cdots \pi$ and O$\cdots$C bonding interactions. Previous studies have found that there is a strong relationship between the strength of bonding and the value of the electron density at the corresponding bond critical point [60–67]. The higher the electron density at a bond critical point, the stronger the corresponding bonding. Thus, based on the value of $\rho$ at the bond critical points, the OH$\cdots \pi$ and O$\cdots$C bonding interactions are weaker than the five OH$\cdots$O hydrogen bondings. Similar bonding interactions are found in **ABW6_1**, **ABW8_1**. The isomer **ABW10_1** has all types of non-covalent interactions identified in this work. This can be ascribed to its size as compared to smaller-sized clusters. The water molecules interact both with the surface of the aminobenzoic acid, establishing OH$\cdots$O and OH$\cdots$N hydrogen bondings, and OH$\cdots \pi$ and O$\cdots$C bonding interactions (see Fig. 9). It is worth noting that similar non-covalent bondings have been recently identified in phenol-water clusters after QTAIM analysis performed at the $\omega$B97XD/aug-cc-pVDZ level of theory [24]. As phenol has no nitrogen atom, the OH$\cdots$N hydrogen bondings have not been identified. Instead, the absence of nitrogen leads to CH$\cdots$O non-covalent bondings, which have been identified as weak hydrogen bondings [24].

To have an idea about the strength of non-covalent bondings in $\text {ABW}_{n}$, we extracted the minimum and the maximum of the electron density, $\rho$, and the Laplacian of the electron density, $\nabla ^{2} \rho$, at bond critical points of non-covalent bondings (see Table 1). Based on the value of $\rho$ at bond critical points, it comes out from Table 1 that the hydrogen bondings are the most robust non-covalent interactions. The OH$\cdots$N hydrogen bonding is less robust than the OH$\cdots$O hydrogen bondings. The results show that the OH$\cdots \pi$ bonding interactions are the weakest non-covalent bondings of aminobenzoic acid-water clusters. The bonding strength trend perfectly agrees with our findings in phenol-water clusters [24]. The OH$\cdots$O hydrogen bondings are the most robust non-covalent bonding identified in phenol-water clusters.

**Table 1:** Minimum and maximum intervals of the electron density, $\rho$, and the Laplacian of the electron density, $\nabla ^{2} \rho$, at bond critical points based on the global minima energy structures of the studied clusters

	$\rho$ ($ea_{0}^{-3}$)		$\nabla ^{2}\rho$ ($ea_{0}^{-5}$)	
Bonding	Min	Max	Min	Max
OH$\cdots$O	0.0023	0.0496	0.0091	0.1171
OH$\cdots$N	0.0189	0.0189	0.0590	0.0590
OH$\cdots \pi$	0.0045	0.0056	0.0173	0.0185
O$\cdots$C	0.0030	0.0062	0.0107	0.0253

The full data of the electron density and its Laplacian at all bond critical points is provided in the supporting information

### Relative population of $\text {ABW}_{n}$ structures

In order to assess the effects of the temperature on the stability of the located structures, we have calculated their relative population/probability for temperature ranging from 20 to 400 K. The probabilities are calculated using the Boltzmann formula within the canonical distribution. The probability of isomer *k* of the cluster $\text {ABW}_{n}$, $P_{n}^{k}(T)$, can be calculated using Eq. 4.

4 $$\begin{aligned} P_{n}^{k}(T)=\dfrac{1}{\sum _{i} \exp \left\{ -\beta \left( G_{i}(T)-G_{k}(T)\right) \right\} }, \end{aligned}$$

where $\beta =k_{B}T$, $k_{B}$ is the Boltzmann constant, and $G_{i}(T)$ is the Gibbs free energy of isomer *i* at temperature *T*. The numerically evaluated relative probabilities of $\text {ABW}_{n}$ clusters are reported in Fig. S4 of the supporting information.

The results show that the most stable configurations dominate the population of the clusters, $\text {ABW}_{n}$, $n=2-10$, and for temperatures ranging from 20 to 400 K. It has been noted that only a few configurations around the most stable one contribute to the population. The results show that for each $\text {ABW}_{n}$ cluster, more than one isomer contributes to its population. The isomers that contribute to the population of the clusters have their relative energies within $\sim$2.0 kcal/mol.

### Solvation free energy and solvation enthalpy

**Fig. 10:**
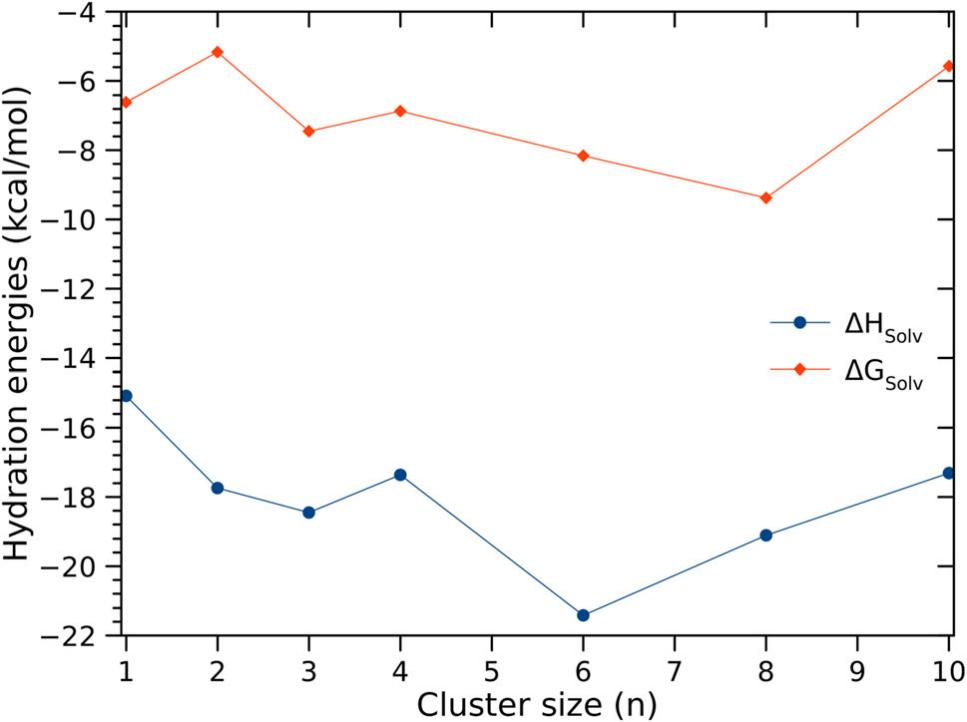
Hydration free energy and hydration enthalpy of aminobenzoic acid at room temperature, calculated using the cluster continuum model at the PW6B95D3/def2-TZVP level of theory

The solvation enthalpy and the solvation free energy of aminobenzoic acid in water are calculated using the cluster continuum solvation model through Eqs. 2 and 3. The calculated hydration free energy and enthalpy at room temperature are reported in Fig. 10 for different cluster sizes *n*.

**Table 2:** Hydration free energy and enthalpy calculated using three implicit solvation models (CPCM, PCM, and SMD) and reported in kcal/mol

Models	$\Delta H_{Solv}$	$\Delta G_{Solv}$
CPCM	$-$5.6	4.0
PCM	$-$5.6	4.0
SMD	$-$15.1	$-$6.6

**Fig. 11:**
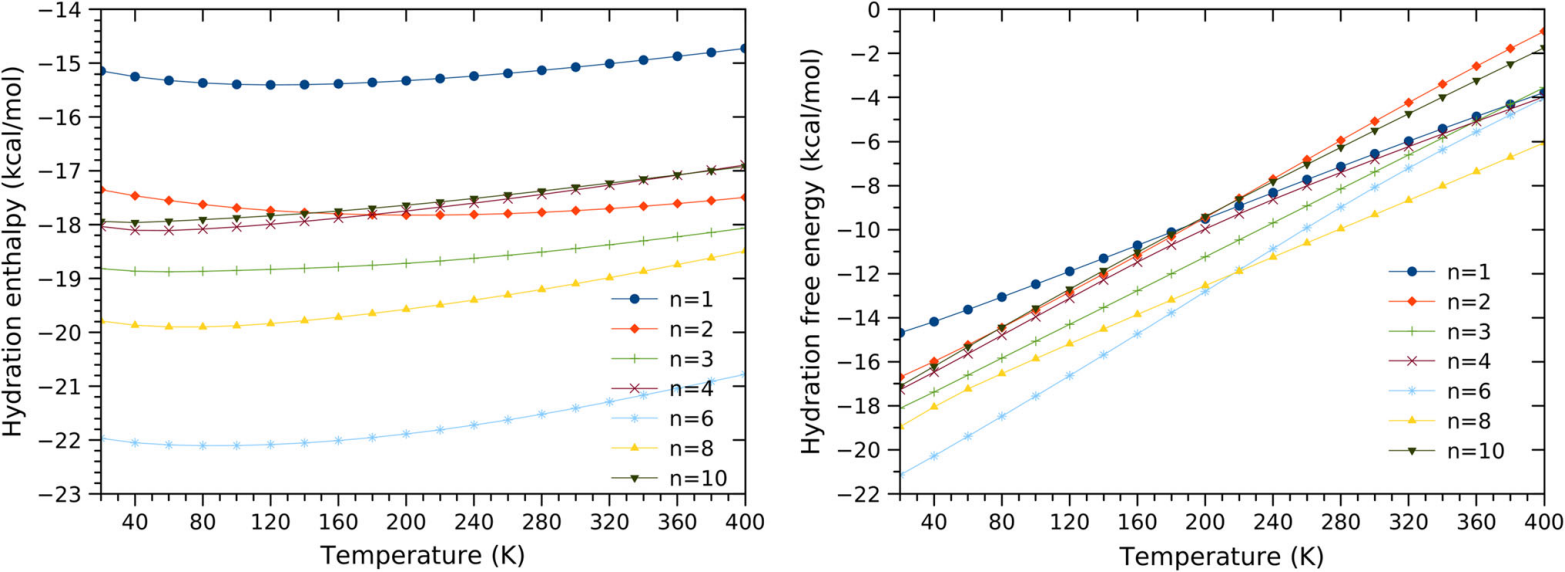
Hydration enthalpy and hydration free energy of aminobenzoic acid calculated for temperatures ranging from 20 to 400 K. The hydration enthalpy and free energy are calculated using the Eqs. 2 and 3, respectively

Examination of Fig. 10 shows that the hydration free energy and enthalpy do not considerably change with the cluster size change. This indicates that one explicit water molecule is enough for the hybrid solvation of aminobenzoic acid. It also indicates that one water molecule is enough in the cluster continuum solvation model. Therefore, we averaged over the estimated values for different cluster sizes to calculate the hydration free energy and the hydration enthalpy. Thus, the hydration enthalpy and the hydration free energy of aminobenzoic acid are numerically estimated to be ${-18.1}$ kcal/mol and ${-7.0}$kcal/mol, respectively. Previously, Turner and coworkers [11] estimated the solvation enthalpy and the solvation free energy of aminobenzoic acid in ethanol, acetonitrile, and water. The solvation enthalpy is estimated using an experimental technique, while the solvation free energy is calculated using molecular dynamics simulations [11]. Turner and coworkers [11] estimated the hydration enthalpy of aminobenzoic acid to be ${-17.8}$kcal/mol. Besides, they estimated the hydration free energy (using molecular dynamics) to be ${-10.1}$kcal/mol. The hydration enthalpy of the aminobenzoic acid estimated in this work is found to be in perfect agreement with the experimental estimate of Turner and coworkers [11]. However, our estimated hydration free energy is found to be underestimated as compared to the reported value of Turner and coworkers [11]. Recently, Li et al. [13] estimated the hydration free energy of the aminobenzoic acid in several solvents, including water, using molecular dynamics simulations. They estimated the hydration free energy of aminobenzoic acid in water to be ${-7.6}$kcal/mol, in perfect agreement with our estimate. Thus, the calculated hydration free energy of Turner and coworkers [11] could be overestimated. Despite the agreement of the calculated hydration enthalpy with the experiment, it is essential to discuss some possibilities that led to the agreement. There is a high probability that the computational methodology used in this work has led to the accuracy of the hydration enthalpy and free energy. However, there is also a chance (that can not be excluded) that the accuracy of the hydration free energy and enthalpy is a result of an error cancellation. As the hydration enthalpy and free energy are calculated using the difference between the energy of $\text {ABW}_{n}$, AB, and $\textrm{W}_{n}$, an overestimation or an underestimation of individual energy could cancel each other and lead to the accuracy of the hydration free energy and enthalpy. Regardless of the source of the accuracy of the present results, the credit for the accuracy goes to our methodology. It is important to note that the accuracy is not accidental as this methodology has been successfully used in our previous work to estimate accurately the solvation free energy of the proton in ammonia [20], methanol [21], and acetonitrile [23].

To assess the effect of the implicit solvation on the calculated hydration free energy and enthalpy, these energies are calculated using two more implicit models: the CPCM and the PCM. The hydration free energy and enthalpy calculated using these models (at room temperature) are reported in Table 2. It can be seen that the CPCM and PCM models predicted the same values for the hydration energy and enthalpy of the aminobenzoic acid. In addition, it has been found that the difference between the predicted values using PCM and the ones predicted using the SMD model is considerably important. Moreover, the CPCM and the PCM models predicted a positive value (4.0kcal/mol) of the hydration free energy. In contrast, the previous estimate based on molecular dynamics predicted ${-10.1}$kcal/mol (as mentioned above). On the other hand, the predicted hydration enthalpy using PCM and CPCM (${-5.6}$kcal/mol) is considerably far from the experimental value of ${-17.8}$kcal/mol. Therefore, the SMD model chosen in this work is better than the CPCM and PCM models for the estimation of the hydration free energy and enthalpy of the aminobenzoic acid.

**Table 3:** Binding energies of aminobenzoic acid-water clusters, $\text {ABW}_{n}$, calculated using twelve different DFT functionals benchmarked to DLPNO-CCSD(T)

$\text {ABW}_{n}$	B3LYP	B3PW91	M052X	M05	M062X	M06	MN15	PBE0	PBE	PW6	TPSS	wB97	DLPNO
$\text {ABW}_{1}$	$-$12.3	$-$11.9	$-$12.1	$-$11.7	$-$12.1	$-$12.0	$-$11.8	$-$12.6	$-$13.3	$-$11.4	$-$12.5	$-$11.5	$-$8.5
$\text {ABW}_{2}$	$-$25.8	$-$24.7	$-$24.6	$-$24.7	$-$24.5	$-$24.2	$-$23.7	$-$26.2	$-$27.6	$-$23.3	$-$26.0	$-$24.2	$-$21.7
$\text {ABW}_{3}$	$-$36.1	$-$34.6	$-$34.8	$-$34.2	$-$34.8	$-$34.1	$-$33.1	$-$36.4	$-$38.6	$-$32.3	$-$36.2	$-$33.4	$-$28.8
$\text {ABW}_{4}$	$-$45.1	$-$43.7	$-$43.2	$-$42.9	$-$43.2	$-$44.0	$-$41.7	$-$45.7	$-$48.6	$-$40.5	$-$45.4	$-$41.8	$-$33.7
$\text {ABW}_{6}$	$-$76.8	$-$73.5	$-$73.2	$-$73.6	$-$73.0	$-$72.5	$-$70.5	$-$77.3	$-$82.2	$-$68.8	$-$77.1	$-$70.8	$-$59.8
$\text {ABW}_{8}$	$-$106.4	$-$101.2	$-$101.2	$-$102.3	$-$100.1	$-$98.8	$-$95.5	$-$107.5	$-$114.6	$-$94.8	$-$107.0	$-$98.3	—–
$\text {ABW}_{10}$	$-$131.9	$-$124.8	$-$125.4	$-$127.0	$-$124.8	$-$123.2	$-$118.8	$-$132.5	$-$141.5	$-$117.4	$-$132.1	$-$121.9	—–
MAD	8.7	7.2	7.1	6.9	7.0	6.9	5.7	9.2	11.6	4.8	9.0	5.9	0.0
MAX	17.0	13.7	13.4	13.8	13.2	12.8	10.7	17.5	22.5	9.0	17.3	11.1	0.0
RMSE	10.0	8.3	8.1	8.1	8.0	7.9	6.5	10.5	13.3	5.5	10.3	6.7	0.0

For DFT functionals, the basis set used in the calculations is def2-TZVP, while for DLPNO-CCSD(T), we used the two-point strategy complete basis set (CBS) extrapolation. Statistical descriptors, including the mean absolute deviation (MAD), the maximum deviation (MAX), and the root mean squared error (RMSE), are calculated in reference to the DLPNO-CCSD(T)/CBS binding energies. To fit the table on the page, some names have been truncated: PW6=PW6B95D3, wB97=$\omega$B97XD, DLPNO=DLPNO-CCSD(T)

After establishing the reliability of our estimated hydration free energy and enthalpy at room temperature, we examine the effect of temperature on the calculated values. The aminobenzoic acid’s hydration free energy and enthalpy as a function of temperature are reported in Fig. 11. As seen in Fig. 11, the hydration enthalpy is less affected and exhibits a minor change with the temperature change. Regarding the hydration free energy of aminobenzoic acid, we noted that it exhibits an almost linear variation with the temperature change (see Fig. 11). This behavior of the hydration free energy and enthalpy has been noted in our previous work on the hydration of phenol [24]. In agreement with the current results, we have found that phenol’s hydration free energy increases linearly as a temperature function.

### Gas phase binding energies and DFT benchmarking

In order to determine the appropriate DFT functional to study the interaction between the aminobenzoic acid and the water molecules, we calculated the binding energies of the studied $\text {ABW}_{n}$ clusters. The binding energies are calculated using twelve different DFT functionals, including Grimme’s empirical dispersions (except the MN15). The methodology provides the functionals, and they are reported in Table 3. The binding energy of $\text {ABW}_{n}$ is calculated using Eq. 5.

5 $$\begin{aligned} \Delta E_{n}= E(\text {ABW}_{n})-E(\text {AB})-nE(\textrm{H}_{2}\textrm{O}), \end{aligned}$$

where *E*(*X*) is the electronic energy of the molecule *X*. For each of the functionals, the molecules involved in Eq. 5 are fully re-optimized using the functional associated with the def2-TZVP basis set. Basis set superposition error has not been considered in this work. To calculate the binding energies, only the most stable configurations of $\text {ABW}_{n}$ have been considered. For each DFT functional, and for each cluster size, the most stable structure has been fully re-optimized. After optimization, it has been found that there is no visual difference between the geometries resulting from different functionals. In addition, the binding energies are also calculated at the DLPNO-CCSD(T)/CBS to serve as a benchmark for the DFT functionals. The calculated binding energies using the twelve functionals and the DLPNO-CCSD(T) method are reported in Table 3. Statistical descriptors, including the mean absolute deviation (MAD), the maximum deviation (MAX), and the root mean squared error (RMSE), are calculated in reference to the DLPNO-CCSD(T)/CBS binding energies. The statistical descriptors are also reported in Table 3.

**Fig. 12:**
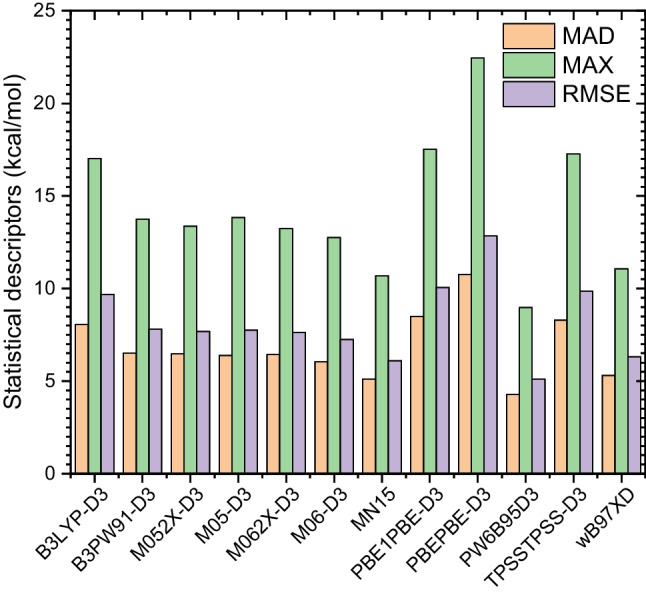
Calculated statistical descriptors related to the studied functionals, including the mean absolute deviation (MAD), the maximum deviation (MAX), and the root mean squared error (RMSE). These values are reported in kcal/mol

It comes out from Table 3 that the MAD varies from 4.8kcal/mol to 11.6kcal/mol, while the RMSE varies from 5.5kcal/mol to 13.3kcal/mol. To easily assess the performance of these functionals, the calculated statistical descriptors are reported in Fig. 12. It can be seen that the PW6B95D3 functional has the smallest MAD and the smallest RMSE. Therefore, the PW6B95D3 functional is the most suitable for studying the aminobenzoic acid-water clusters. Besides, we note that the PBEPBE-D3 functional has the highest MAD and RMSE, highlighting the unsuitability of the functional for the aminobenzoic acid-water clusters. The results show that the first three most suitable DFT functionals are classified in the following order: PW6B95D3 > MN15 > $\omega$B97XD. It is worth mentioning that the most suitable DFT functional, PW6B95D3, has a MAD of 4.8kcal/mol, which is not negligible. This considerable MAD could be ascribed to the basis set superposition error. Therefore, counterpoise corrections should be considered to reduce the basis set superposition error for a more accurate description. The functional PW6B95D3 is the most suitable for studying molecular clusters [36–39]. Consequently, based on this work and our past investigations, the PW6B95D3 functional is recommended for studying molecular clusters.

## Conclusions

This work thoroughly explored the potential energy surfaces (PESs) of aminobenzoic acid-water clusters, $\text {ABW}_{n}$, $n=1-10$. The exploration started with classical molecular dynamics, followed by complete optimization at the PW6B95D3/def2-TZVP level of theory. In addition, we performed a quantum theory of atoms in molecules (QTAIM) analysis to understand the nature of non-covalent bonding in the clusters. Relative populations of the clusters as a function of temperature are also reported. Using the located structures of aminobenzoic acid-water clusters, we calculated the hydration enthalpy and the hydration free energy of the aminobenzoic acid using the cluster continuum solvation model. Finally, we calculated the binding energies of the most stable configurations using twelve DFT functionals and DLPNO-CCSD(T), including empirical dispersion. The binding energies are evaluated to benchmark the functionals.

Several stable structures have been located on the PESs of $\text {ABW}_{n}$, $n=1-10$. We noted that the most stable configurations are obtained when the water molecules establish strong OH$\cdots$O hydrogen bondings. Moreover, it has been found that the structures where the water molecules interact with the carboxyl group are more stable than those in which the water molecules interact with the amino group. The QTAIM analysis reveals that OH$\cdots$O hydrogen bondings (with carboxyl group) are more robust than the OH$\cdots$N hydrogen bonding (with the amino group). In addition to OH$\cdots$O and OH$\cdots$N hydrogen bondings, we have identified two more non-covalent interactions: the OH$\cdots \pi$ and the O$\cdots$C bonding interactions. The OH$\cdots$O hydrogen bonding is found to be the most robust non-covalent interactions, while the OH$\cdots \pi$ are found to be the weakest. Elsewhere, the study of temperature effects on the population of the $\text {ABW}_{n}$ clusters shows that the most stable isomers dominate the population of clusters.

The hydration free energy and the hydration enthalpy of the aminobenzoic acid at room temperature are estimated to be ${-7.0}$kcal/mol and ${-18.1}$kcal/mol. The estimated hydration enthalpy is found to be in perfect agreement with an experimental estimate. As for the hydration free energy, it agrees with the estimate based on molecular dynamics simulations. Besides, we examined the effects of temperature on the calculated hydration free energy and enthalpy. The results show that the hydration enthalpy is not affected by the change in temperature, while the hydration free energy exhibits a linear variation as a function of temperature.

To recommend the most suitable DFT functional for the study of the aminobenzoic acid-water clusters, we calculated the binding energy of the most stable configurations in the gas phase. The binding energies are calculated using twelve DFT functionals, including empirical dispersion benchmarked to DLPNO-CCSD(T)/CBS. The def2-TZVP basis set was used in association with the functionals. The results show that the PW6B95D3 functional has the smallest mean absolute deviation (MAD) and the slightest root mean squared error (RMSE). In addition, we have found that the three most suitable DFT functionals with negligible differences are PW6B95D3 > MN15 > $\omega$B97XD. We also noted that the PBEPBE-D3 functional has the highest MAD and RMSE compared to the DLPNO-CCSD(T)/CBS level of theory. Finally, based on the current results and our previous benchmarks on molecular clusters, the PW6B95D3 functional can be considered for studying non-covalent bonding systems.

### Supplementary Information

Below is the link to the electronic supplementary material.

Supplementary file 1 (pdf 5455 KB)

Supplementary file 2 (pdf 31 KB)

Supplementary file 3 (pdf 332 KB)

## Data Availability

The data used in this work is provided in the manuscript or in the supporting information.

## Declarations

### Supporting information

A complete list of the structures of $\text {ABW}_n$ for $n=6$, $n=8$, and $n=10$, as well as their relative energies, are reported in Figs. S1–S3, respectively. In addition, the curves of the relative population of the studied clusters are reported in Fig. S4.
