# Supplementary material for: Hydration of p - aminobenzoic acid: structures and non-covalent bondings of aminobenzoic acid-water clusters
Source: J Mol Model. 2024 Jan 12;30(2):38. doi: 10.1007/s00894-023-05810-2 (PMC10786749; doi:10.1007/s00894-023-05810-2)
Supplement: Supplementary file 1 — (pdf 5455 KB) [file 894_2023_5810_MOESM1_ESM.pdf]

Structures and Non-Covalent Bondings of Aminobenzoic Acid-Water clusters

Diane Anni[◇,a], Jean Claude Amika Mbema[◇,a], Alhadji Malloum[◇,†,*] and Jeanet Conradie[†,‡]

[◇] Department of Physics, Faculty of Science, University of Maroua, PO BOX 46, Maroua, Cameroon.

[†] Department of Chemistry, University of the Free State, PO BOX 339, Bloemfontein 9300, South Africa.

[‡]Department of Chemistry, UiT - The Arctic University of Norway, N-9037 Tromsø, Norway.

December 4, 2023

SUPPORTING INFORMATION:

KEYWORDS: Aminobenzoic acid; Hydration free energy; Solvation; non-Covalent bondings; QTAIM analysis

1 Data description

The data reported in this supporting information comprises the full lists of the located isomers of the aminobenzoic acid-water clusters, and their relative electronic energies as calculated at the PW6B95D3/def2TZVP level of theory. These lists are reported in Figure 5, Figure 6, and Figure 7 for the aminobenzoic acid-water hexamer, octamer, and decamer, respectively. In addition, we have also reported in this supporting information, the relative population of the studied clusters for temperatures ranging from 20 K to 400 K. The relative population are reported in Figure 8.

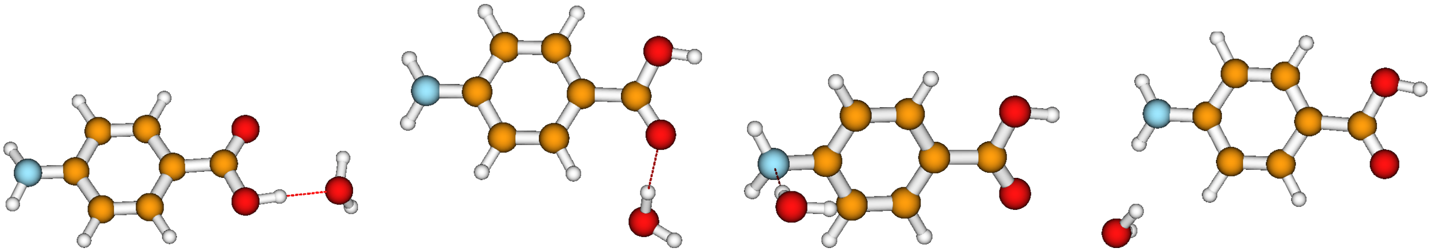

a ABW1_1 0.0 (0.0) 0.0 **b** ABW1_2 1.9 (1.9) 1.6 **c** ABW1_3 2.9 (2.9) 2.0 **d** ABW1_4 2.6 (2.5) 3.2

Fig. S 1 Structures and relative energies of ABW_1 as optimized at the PW6B95D3/def2TZVP level of theory using three implicit solvation models (CPCM, PCM, and SMD). Numbers are the calculated relative energies (in kcal/mol). The first number is the relative energy obtained using the CPCM model, the second is obtained with PCM, and the third is obtained with SMD. The corresponding relative energies reported in the manuscript are obtained using the SMD solvation model.

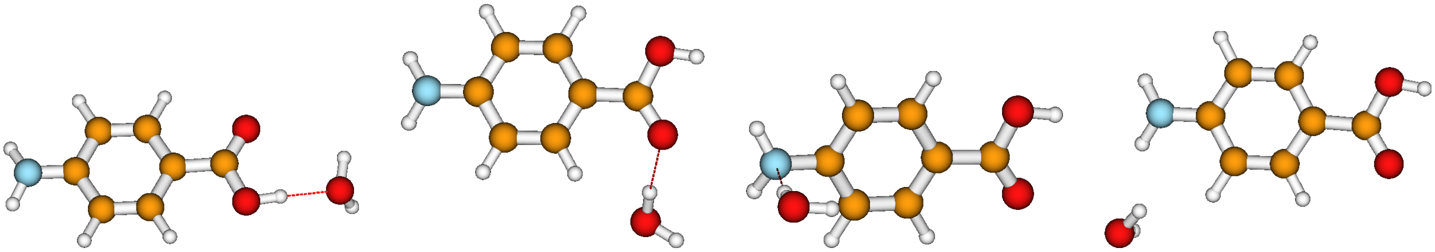

a ABW1_1 0.0 (0.0) **b** ABW1_2 1.6 (2.1) **c** ABW1_3 2.0 (1.4) **d** ABW1_4 3.2 (3.4)

Fig. S 2 Structures and relative energies of ABW_1 as optimized at the PW6B95D3/def2TZVP and MP2/def2TZVP levels of theory using the SMD implicit solvation model. Numbers are the calculated relative energies (in kcal/mol). Relative energies in MP2/def2TZVP are reported in parenthesis.

* *E-mail: MalloumA@ufs.ac.za; Tel: +237 695 15 10 56*

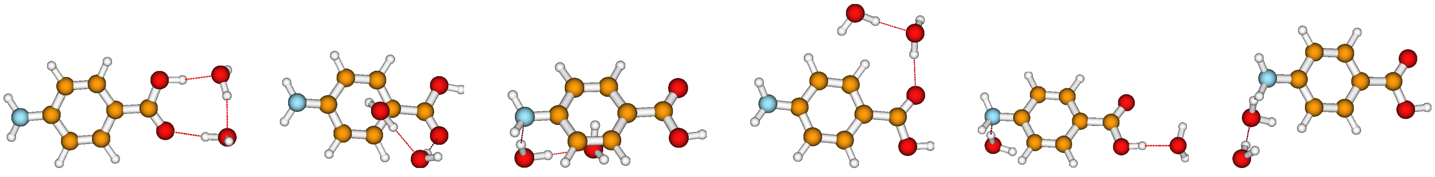

a ABW2_1 0.0 (0.0) **b** ABW2_2 1.3 (1.4) **c** ABW2_3 1.8 (1.1) **d** ABW2_4 2.2 (1.1) **e** ABW2_5 2.2 (0.9) **f** ABW2_6 3.5 (3.0)

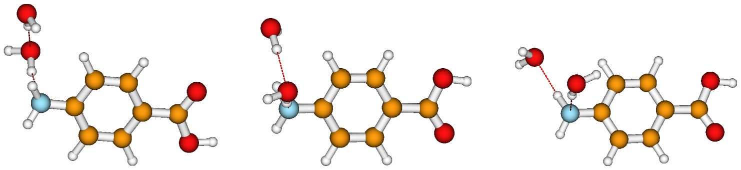

g ABW2_7 3.8 (3.2) **h** ABW2_8 3.9 (3.1) **i** ABW2_9 4.6 (3.4)

Fig. S 3 Structures and relative energies of ABW_2 as optimized at the PW6B95D3/def2TZVP and MP2/def2TZVP levels of theory using the SMD implicit solvation model. Numbers are the calculated relative energies (in kcal/mol). Relative energies in MP2/def2TZVP are reported in parenthesis.

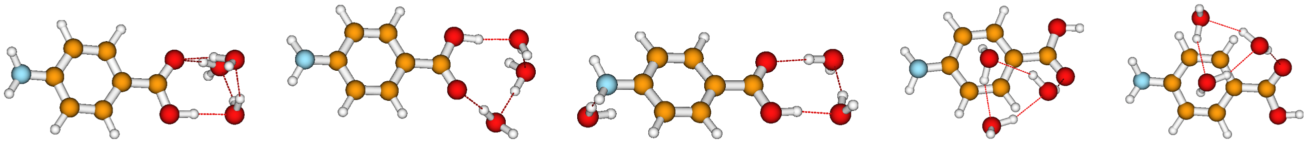

a ABW3_1 0.0 (0.4) **b** ABW3_2 0.2 (0.0) **c** ABW3_3 1.4 (0.4) **d** ABW3_4 1.8 (2.5) **e** ABW3_5 1.9 (2.3)

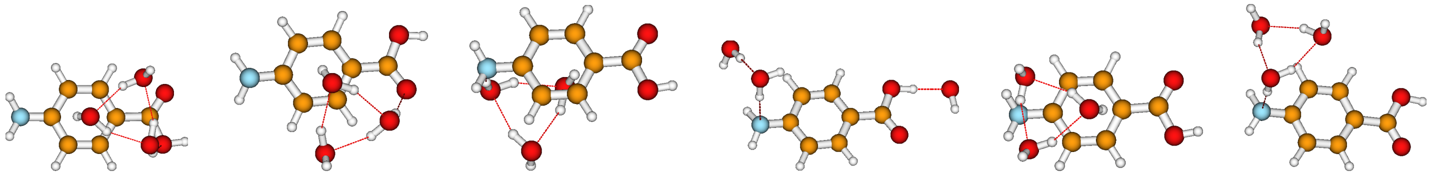

f ABW3_6 2.0 (2.4) **g** ABW3_7 2.0 (2.2) **h** ABW3_8 2.7 (2.0) **i** ABW3_9 2.9 (1.7) **j** ABW3_10 3.7 (3.4) **k** ABW3_11 4.7 (3.4)

Fig. S 4 Structures and relative energies of ABW_3 as optimized at the PW6B95D3/def2TZVP and MP2/def2TZVP levels of theory using the SMD implicit solvation model. Numbers are the calculated relative energies (in kcal/mol). Relative energies in MP2/def2TZVP are reported in parenthesis.

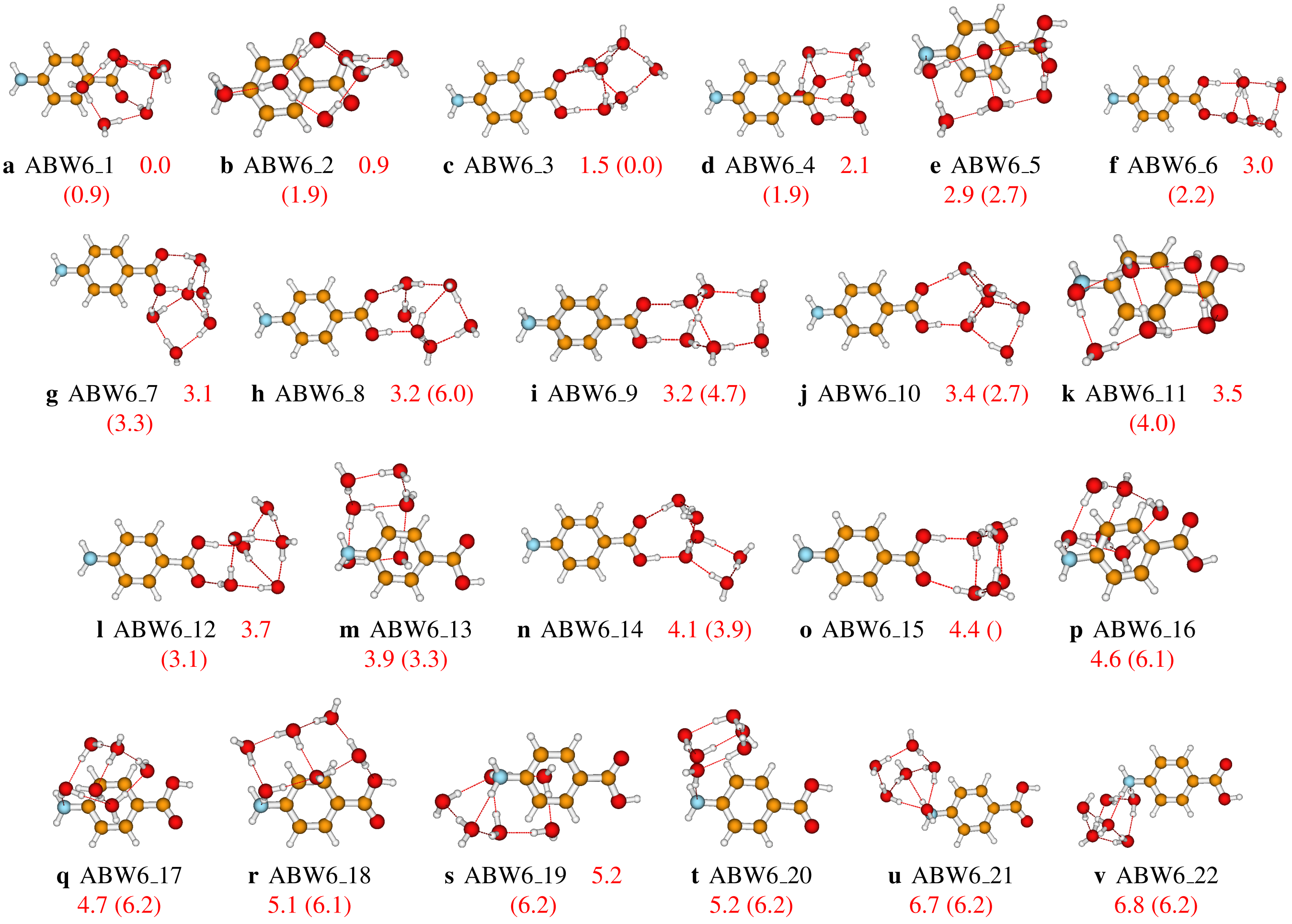

Fig. S 5 Structures and relative energies of ABW_6 as optimized at the PW6B95D3/def2TZVP level of theory. Numbers are the calculated relative energies (in kcal/mol). Numbers in parenthesis are relative energies calculated at the PW6B95D3/cc-pVDZ level of theory.

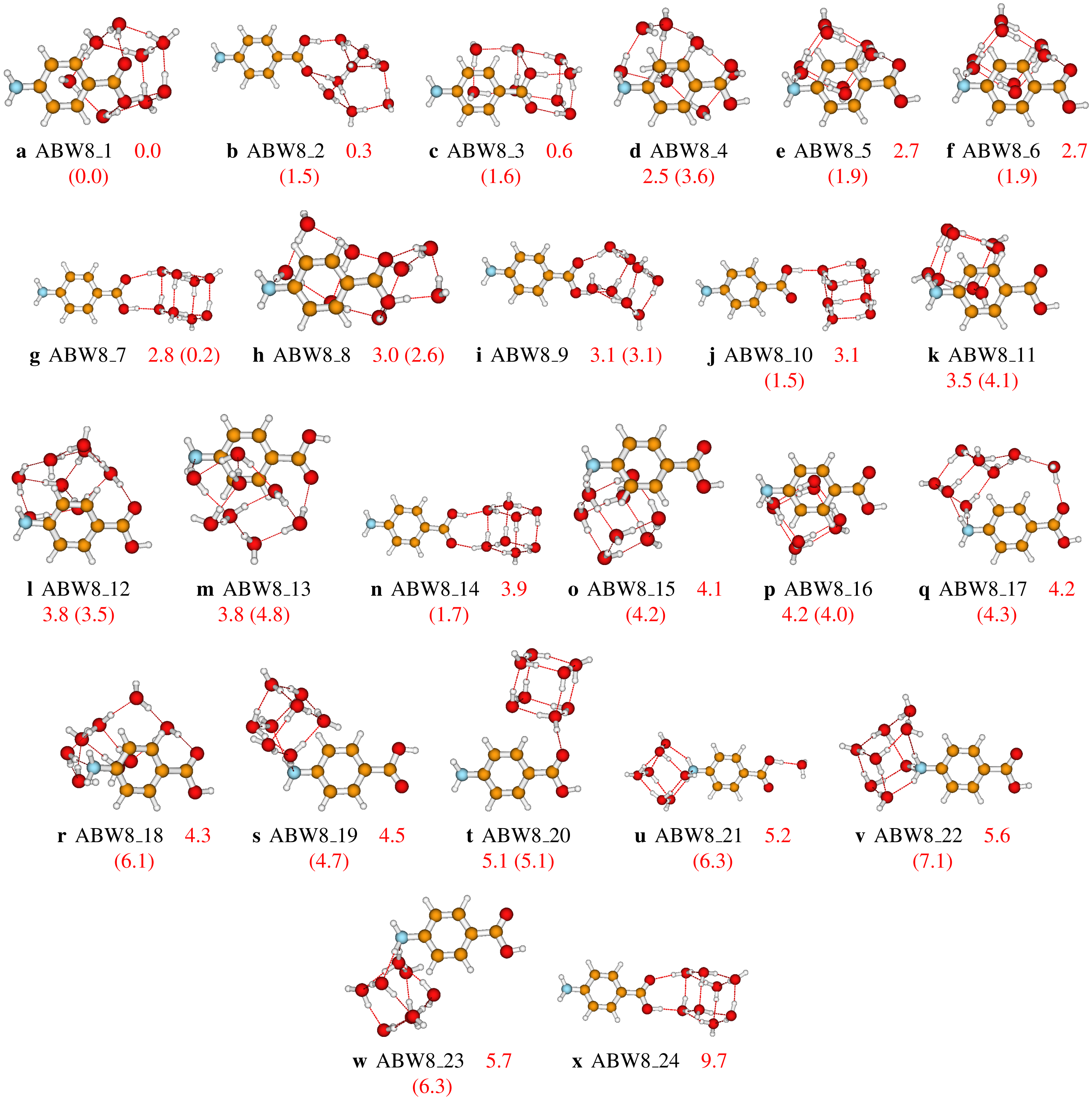

Fig. S 6 Structures and relative energies of ABW_8 as optimized at the PW6B95D3/def2TZVP level of theory. Numbers are the calculated relative energies (in kcal/mol). Numbers in parenthesis are relative energies calculated at the PW6B95D3/cc-pVDZ level of theory.

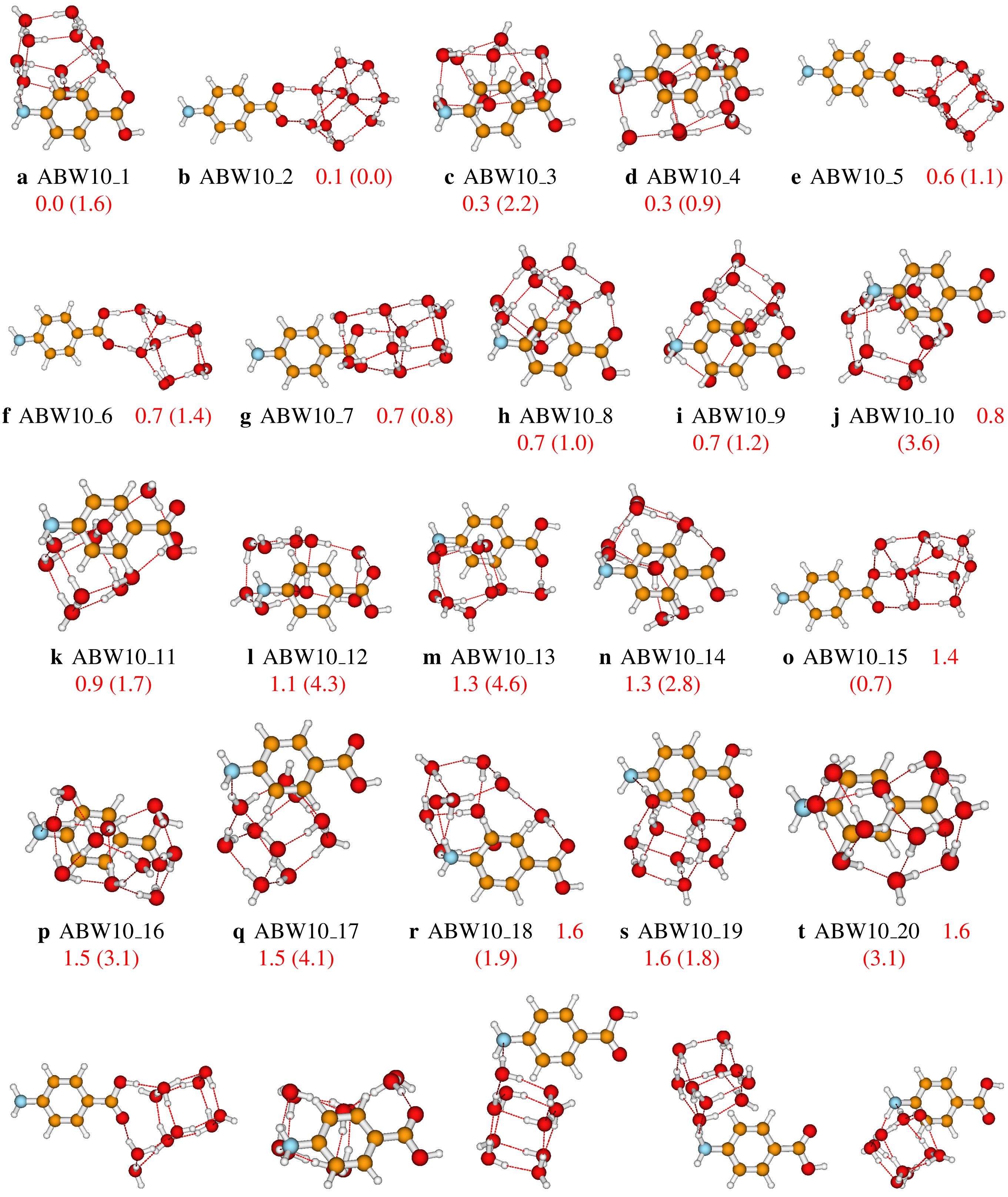

a ABW10_1 0.0 (1.6)
b ABW10_2 0.1 (0.0)
c ABW10_3 0.3 (2.2)
d ABW10_4 0.3 (0.9)
e ABW10_5 0.6 (1.1)
f ABW10_6 0.7 (1.4)
g ABW10_7 0.7 (0.8)
h ABW10_8 0.7 (1.0)
i ABW10_9 0.7 (1.2)
j ABW10_10 0.8 (3.6)
k ABW10_11 0.9 (1.7)
l ABW10_12 1.1 (4.3)
m ABW10_13 1.3 (4.6)
n ABW10_14 1.3 (2.8)
o ABW10_15 1.4 (0.7)
p ABW10_16 1.5 (3.1)
q ABW10_17 1.5 (4.1)
r ABW10_18 1.6 (1.9)
s ABW10_19 1.6 (1.8)
t ABW10_20 1.6 (3.1)
u ABW10_21 1.7 (1.5)
v ABW10_22 2.2 (3.6)
w ABW10_23 2.4 (3.7)
x ABW10_24 2.4 (3.6)
y ABW10_25 5.2 (5.9)

Fig. S 7 Structures and relative energies of ABW_{10} as optimized at the PW6B95D3/def2TZVP level of theory. Numbers are the calculated relative energies (in kcal/mol). Numbers in parenthesis are relative energies calculated at the PW6B95D3/cc-pVDZ level of theory.

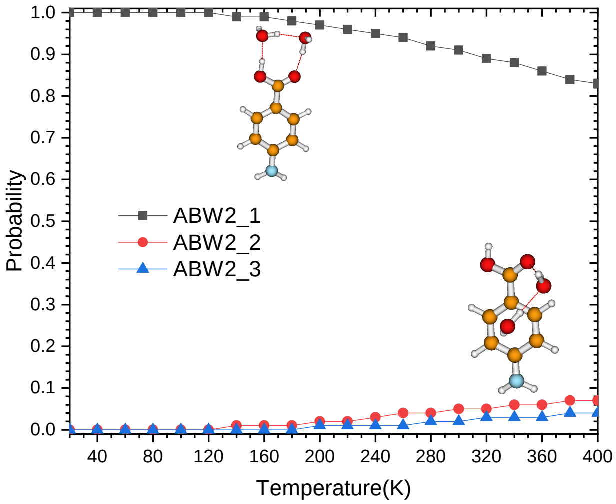

a ABW_2

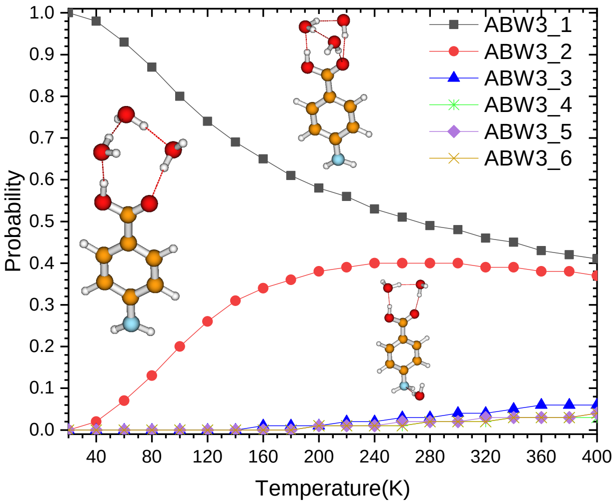

b ABW_3

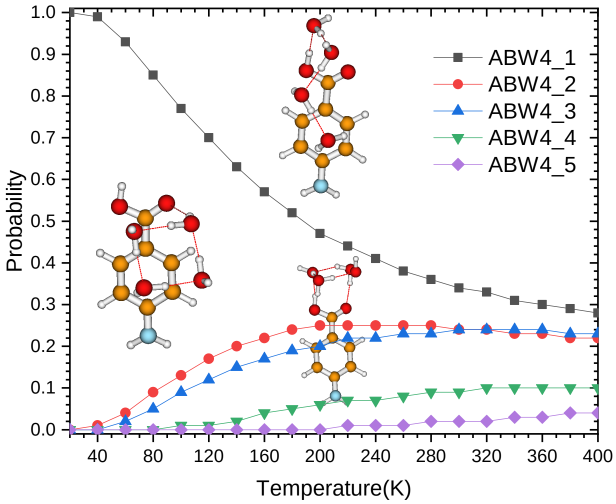

c ABW_4

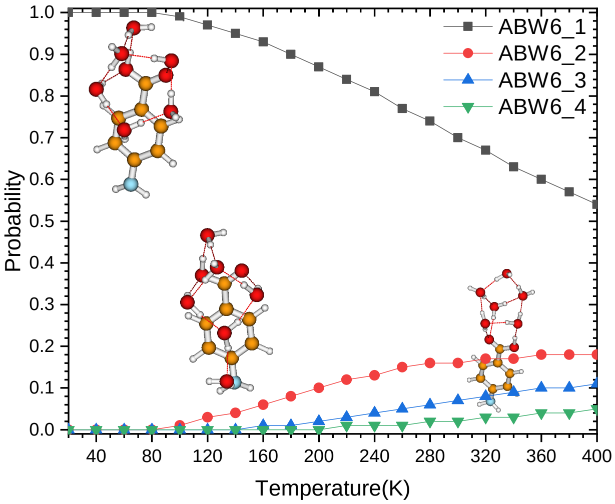

d ABW_6

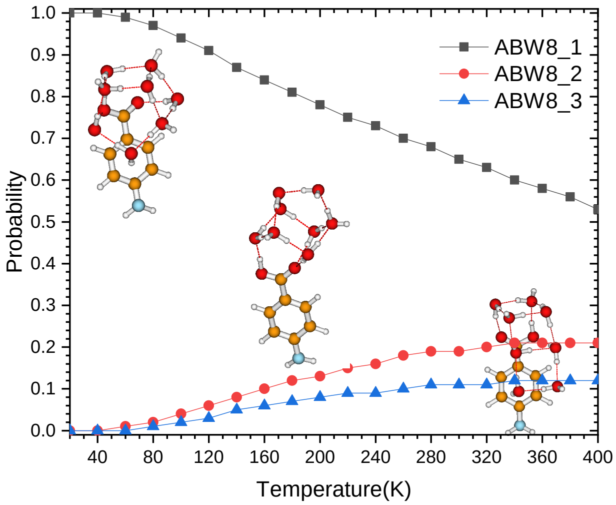

e ABW_8

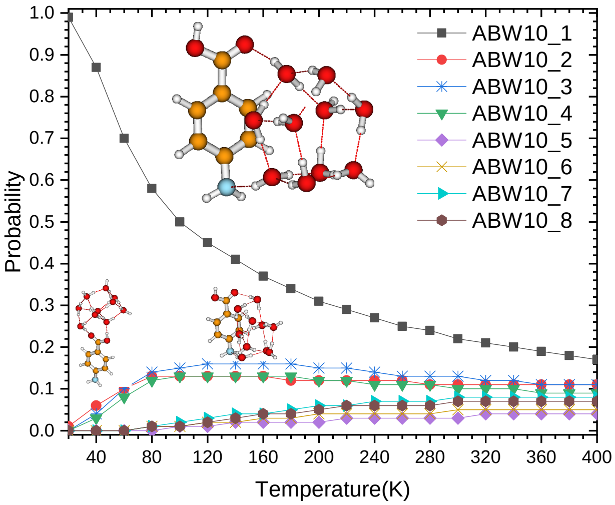

f ABW_{10}

Fig. S 8 Relative population (probability) of the located isomers reported for each cluster size.
