# Supplementary material for: Hydration of p - aminobenzoic acid: structures and non-covalent bondings of aminobenzoic acid-water clusters
Source: J Mol Model. 2024 Jan 12;30(2):38. doi: 10.1007/s00894-023-05810-2 (PMC10786749; doi:10.1007/s00894-023-05810-2)
Supplement: Supplementary file 2 — (pdf 31 KB) [file 894_2023_5810_MOESM2_ESM.pdf]

| Amino1 | | Name | Atoms | Rho | Laplacian Rh | Ellipticity | K | BPL-GBL |
|---|---|---|---|---|---|---|---|---|
| | 1 | BCP1 | C17 - O19 | 0,3193 | -0,6696 | 0,0291 | 0,5189 | 0,0006 |
| | 2 | BCP2 | O1 - H20 | 0,0443 | 0,1157 | 0,0350 | 0,0076 | 0,0004 |
| | 3 | BCP3 | O1 - H2 | 0,3574 | -2,4993 | 0,0165 | 0,6885 | 0,0001 |
| | 4 | BCP4 | O1 - H3 | 0,3579 | -2,5039 | 0,0164 | 0,6897 | 0,0001 |
| | 5 | BCP5 | C4 - C5 | 0,3162 | -0,9304 | 0,1858 | 0,3364 | 0,0002 |
| | 6 | BCP6 | C5 - C6 | 0,3282 | -0,9845 | 0,2226 | 0,3611 | 0,0001 |
| | 7 | BCP7 | C6 - H11 | 0,2886 | -1,0292 | 0,0231 | 0,2968 | 0,0000 |
| | 8 | BCP8 | C6 - C7 | 0,3153 | -0,9389 | 0,1890 | 0,3359 | 0,0004 |
| | 9 | BCP9 | C8 - C9 | 0,3290 | -0,9886 | 0,2230 | 0,3627 | 0,0000 |
| | 10 | BCP10 | C7 - C8 | 0,3150 | -0,9377 | 0,1876 | 0,3352 | 0,0004 |
| | 11 | BCP11 | C7 - N14 | 0,3271 | -1,0720 | 0,0837 | 0,4826 | 0,0005 |
| | 12 | BCP12 | C4 - C9 | 0,3168 | -0,9351 | 0,1852 | 0,3375 | 0,0001 |
| | 13 | BCP13 | C8 - H12 | 0,2886 | -1,0297 | 0,0229 | 0,2969 | 0,0000 |
| | 14 | BCP14 | C5 - H10 | 0,2927 | -1,0642 | 0,0088 | 0,3028 | 0,0000 |
| | 15 | BCP15 | C9 - H13 | 0,2919 | -1,0592 | 0,0084 | 0,3015 | 0,0000 |
| | 16 | BCP16 | N14 - H16 | 0,3456 | -1,8667 | 0,0414 | 0,5196 | 0,0001 |
| | 17 | BCP17 | N14 - H15 | 0,3457 | -1,8671 | 0,0414 | 0,5197 | 0,0001 |
| | 18 | BCP18 | C4 - C17 | 0,2869 | -0,8223 | 0,1555 | 0,2811 | 0,0002 |
| | 19 | BCP19 | C17 - O18 | 0,4141 | -0,3120 | 0,0962 | 0,7491 | 0,0002 |
| | 20 | BCP20 | O19 - H20 | 0,3224 | -2,2051 | 0,0114 | 0,6131 | 0,0005 |

| Amino2 | | | | | | | | |
|---|---|---|---|---|---|---|---|---|
| | 1 | BCP1 | O1 - H23 | 0,0477 | 0,1166 | 0,0306 | 0,0097 | 0,0004 |
| | 2 | BCP2 | O1 - H2 | 0,3392 | -2,3345 | 0,0159 | 0,6486 | 0,0001 |
| | 3 | BCP3 | O1 - H3 | 0,3589 | -2,4972 | 0,0147 | 0,6897 | 0,0001 |
| | 4 | BCP4 | H2 - O4 | 0,0335 | 0,1063 | 0,0450 | 0,0020 | 0,0023 |
| | 5 | BCP5 | O4 - H5 | 0,3598 | -2,4977 | 0,0156 | 0,6907 | 0,0001 |
| | 6 | BCP6 | C20 - O21 | 0,4094 | -0,3341 | 0,0913 | 0,7380 | 0,0003 |
| | 7 | BCP7 | H6 - O21 | 0,0299 | 0,1057 | 0,0162 | 0,0000 | 0,0056 |
| | 8 | BCP8 | O4 - H6 | 0,3437 | -2,3770 | 0,0167 | 0,6592 | 0,0001 |
| | 9 | BCP9 | C7 - C20 | 0,2878 | -0,8266 | 0,1580 | 0,2830 | 0,0002 |
| | 10 | BCP10 | C7 - C8 | 0,3160 | -0,9296 | 0,1849 | 0,3360 | 0,0002 |
| | 11 | BCP11 | C8 - C9 | 0,3286 | -0,9862 | 0,2232 | 0,3618 | 0,0001 |
| | 12 | BCP12 | C9 - H14 | 0,2886 | -1,0297 | 0,0231 | 0,2969 | 0,0000 |
| | 13 | BCP13 | C9 - C10 | 0,3153 | -0,9391 | 0,1879 | 0,3358 | 0,0004 |
| | 14 | BCP14 | C7 - C12 | 0,3166 | -0,9342 | 0,1847 | 0,3371 | 0,0002 |
| | 15 | BCP15 | C10 - C11 | 0,3150 | -0,9381 | 0,1867 | 0,3352 | 0,0004 |
| | 16 | BCP16 | C10 - N17 | 0,3277 | -1,0740 | 0,0836 | 0,4855 | 0,0005 |
| | 17 | BCP17 | C11 - C12 | 0,3292 | -0,9895 | 0,2235 | 0,3631 | 0,0000 |
| | 18 | BCP18 | C11 - H15 | 0,2886 | -1,0299 | 0,0229 | 0,2969 | 0,0000 |
| | 19 | BCP19 | C8 - H13 | 0,2928 | -1,0650 | 0,0086 | 0,3030 | 0,0000 |

| | | | | | | | |
|---|---|---|---|---|---|---|---|
| 20 | BCP20 | C12 - H16 | 0,2921 | -1,0603 | 0,0083 | 0,3017 | 0,0000 |
| 21 | BCP21 | N17 - H18 | 0,3457 | -1,8702 | 0,0414 | 0,5204 | 0,0001 |
| 22 | BCP22 | N17 - H19 | 0,3456 | -1,8693 | 0,0414 | 0,5202 | 0,0001 |
| 23 | BCP23 | C20 - O22 | 0,3239 | -0,6527 | 0,0311 | 0,5310 | 0,0008 |
| 24 | BCP24 | O22 - H23 | 0,3164 | -2,1480 | 0,0108 | 0,5990 | 0,0007 |

Amino3

| | | | | | | | |
|---|---|---|---|---|---|---|---|
| 1 | BCP1 | H2 - O24 | 0,0233 | 0,0875 | 0,0353 | -0,0018 | 0,0068 |
| 2 | BCP2 | O1 - H2 | 0,3488 | -2,4142 | 0,0170 | 0,6689 | 0,0001 |
| 3 | BCP3 | O1 - H3 | 0,3597 | -2,4975 | 0,0162 | 0,6906 | 0,0001 |
| 4 | BCP4 | O1 - H6 | 0,0265 | 0,0932 | 0,0709 | -0,0008 | 0,0056 |
| 5 | BCP5 | O4 - H26 | 0,0480 | 0,1134 | 0,0149 | 0,0102 | 0,0003 |
| 6 | BCP6 | O4 - H5 | 0,3453 | -2,3787 | 0,0148 | 0,6602 | 0,0001 |
| 7 | BCP7 | O4 - H6 | 0,3454 | -2,3798 | 0,0148 | 0,6605 | 0,0001 |
| 8 | BCP8 | C23 - O24 | 0,4056 | -0,3873 | 0,0808 | 0,7298 | 0,0003 |
| 9 | BCP9 | H9 - O24 | 0,0239 | 0,0894 | 0,0372 | -0,0017 | 0,0069 |
| 10 | BCP10 | H5 - O7 | 0,0264 | 0,0924 | 0,0688 | -0,0008 | 0,0052 |
| 11 | BCP11 | O7 - H8 | 0,3598 | -2,4982 | 0,0162 | 0,6908 | 0,0001 |
| 12 | BCP12 | O7 - H9 | 0,3484 | -2,4122 | 0,0169 | 0,6684 | 0,0001 |
| 13 | BCP13 | C10 - C23 | 0,2901 | -0,8371 | 0,1626 | 0,2876 | 0,0001 |
| 14 | BCP14 | C10 - C11 | 0,3155 | -0,9280 | 0,1832 | 0,3351 | 0,0002 |
| 15 | BCP15 | C11 - C12 | 0,3291 | -0,9886 | 0,2247 | 0,3629 | 0,0001 |
| 16 | BCP16 | C12 - C13 | 0,3147 | -0,9368 | 0,1857 | 0,3347 | 0,0004 |
| 17 | BCP17 | C12 - H17 | 0,2887 | -1,0306 | 0,0231 | 0,2971 | 0,0000 |
| 18 | BCP18 | C10 - C15 | 0,3162 | -0,9326 | 0,1829 | 0,3362 | 0,0001 |
| 19 | BCP19 | C13 - C14 | 0,3146 | -0,9365 | 0,1848 | 0,3344 | 0,0004 |
| 20 | BCP20 | C13 - N20 | 0,3291 | -1,0773 | 0,0844 | 0,4926 | 0,0005 |
| 21 | BCP21 | C14 - C15 | 0,3297 | -0,9923 | 0,2248 | 0,3643 | 0,0000 |
| 22 | BCP22 | C14 - H18 | 0,2888 | -1,0308 | 0,0230 | 0,2971 | 0,0000 |
| 23 | BCP23 | C11 - H16 | 0,2928 | -1,0655 | 0,0083 | 0,3030 | 0,0000 |
| 24 | BCP24 | N20 - H22 | 0,3458 | -1,8782 | 0,0415 | 0,5221 | 0,0001 |
| 25 | BCP25 | C15 - H19 | 0,2922 | -1,0616 | 0,0080 | 0,3019 | 0,0000 |
| 26 | BCP26 | N20 - H21 | 0,3458 | -1,8788 | 0,0415 | 0,5223 | 0,0001 |
| 27 | BCP27 | C23 - O25 | 0,3256 | -0,6510 | 0,0314 | 0,5353 | 0,0007 |
| 28 | BCP28 | O25 - H26 | 0,3152 | -2,1311 | 0,0108 | 0,5952 | 0,0006 |

Amino4

| | | | | | | | |
|---|---|---|---|---|---|---|---|
| 1 | BCP1 | O1 - H2 | 0,3414 | -2,3510 | 0,0161 | 0,6530 | 0,0001 |
| 2 | BCP2 | O1 - H3 | 0,3582 | -2,4829 | 0,0157 | 0,6871 | 0,0001 |
| 3 | BCP3 | O1 - H5 | 0,0333 | 0,1045 | 0,0221 | 0,0019 | 0,0005 |
| 4 | BCP4 | O4 - H6 | 0,3600 | -2,4965 | 0,0154 | 0,6907 | 0,0001 |
| 5 | BCP5 | O4 - H5 | 0,3388 | -2,3287 | 0,0164 | 0,6472 | 0,0001 |
| 6 | BCP6 | H3 - O28 | 0,0085 | 0,0354 | 0,1922 | -0,0021 | 0,0522 |
| 7 | BCP7 | O4 - H8 | 0,0353 | 0,1071 | 0,0215 | 0,0029 | 0,0006 |
| 8 | BCP8 | O7 - H8 | 0,3358 | -2,3029 | 0,0160 | 0,6405 | 0,0001 |
| 9 | BCP9 | O7 - H29 | 0,0463 | 0,1154 | 0,0191 | 0,0089 | 0,0009 |
| 10 | BCP10 | O7 - H9 | 0,3589 | -2,4933 | 0,0147 | 0,6891 | 0,0001 |
| 11 | BCP11 | H2 - O10 | 0,0308 | 0,0997 | 0,0269 | 0,0009 | 0,0003 |

| | | | | | | | |
|---|---|---|---|---|---|---|---|
| 12 | BCP12 | O10 - H11 | 0,3579 | -2,4925 | 0,0168 | 0,6879 | 0,0001 |
| 13 | BCP13 | O10 - H12 | 0,3568 | -2,4782 | 0,0170 | 0,6847 | 0,0001 |
| 14 | BCP14 | O10 - C18 | 0,0049 | 0,0179 | 1,9631 | -0,0010 | 0,4127 |
| 15 | BCP15 | H12 - C15 | 0,0056 | 0,0185 | 1,4644 | -0,0010 | 0,1015 |
| 16 | BCP16 | C13 - C14 | 0,3163 | -0,9311 | 0,1858 | 0,3367 | 0,0002 |
| 17 | BCP17 | C14 - C15 | 0,3277 | -0,9823 | 0,2212 | 0,3601 | 0,0001 |
| 18 | BCP18 | C15 - C16 | 0,3148 | -0,9361 | 0,1882 | 0,3348 | 0,0004 |
| 19 | BCP19 | C16 - C17 | 0,3148 | -0,9372 | 0,1861 | 0,3348 | 0,0004 |
| 20 | BCP20 | C13 - C18 | 0,3170 | -0,9362 | 0,1842 | 0,3378 | 0,0001 |
| 21 | BCP21 | C17 - C18 | 0,3291 | -0,9894 | 0,2221 | 0,3629 | 0,0000 |
| 22 | BCP22 | C14 - H19 | 0,2924 | -1,0630 | 0,0087 | 0,3024 | 0,0000 |
| 23 | BCP23 | C15 - H20 | 0,2887 | -1,0304 | 0,0230 | 0,2970 | 0,0000 |
| 24 | BCP24 | C17 - H21 | 0,2888 | -1,0310 | 0,0227 | 0,2971 | 0,0000 |
| 25 | BCP25 | C18 - H22 | 0,2920 | -1,0600 | 0,0083 | 0,3016 | 0,0000 |
| 26 | BCP26 | C16 - N23 | 0,3278 | -1,0753 | 0,0846 | 0,4853 | 0,0005 |
| 27 | BCP27 | N23 - H24 | 0,3456 | -1,8698 | 0,0413 | 0,5203 | 0,0001 |
| 28 | BCP28 | N23 - H25 | 0,3457 | -1,8706 | 0,0414 | 0,5205 | 0,0001 |
| 29 | BCP29 | C13 - C26 | 0,2871 | -0,8232 | 0,1549 | 0,2813 | 0,0002 |
| 30 | BCP30 | C26 - O27 | 0,4147 | -0,3035 | 0,0973 | 0,7504 | 0,0002 |
| 31 | BCP31 | C26 - O28 | 0,3191 | -0,6729 | 0,0295 | 0,5182 | 0,0006 |
| 32 | BCP32 | O28 - H29 | 0,3191 | -2,1680 | 0,0113 | 0,6044 | 0,0005 |

Amino6

| | | | | | | | |
|---|---|---|---|---|---|---|---|
| 1 | BCP1 | O1 - H18 | 0,0356 | 0,1089 | 0,0253 | 0,0028 | 0,0004 |
| 2 | BCP2 | O1 - H2 | 0,3600 | -2,4972 | 0,0156 | 0,6909 | 0,0000 |
| 3 | BCP3 | O1 - H3 | 0,3391 | -2,3297 | 0,0166 | 0,6478 | 0,0001 |
| 4 | BCP4 | O4 - H6 | 0,3358 | -2,3098 | 0,0166 | 0,6417 | 0,0001 |
| 5 | BCP5 | O4 - H5 | 0,3593 | -2,5096 | 0,0152 | 0,6925 | 0,0000 |
| 6 | BCP6 | H6 - O7 | 0,0355 | 0,1075 | 0,0198 | 0,0030 | 0,0009 |
| 7 | BCP7 | O7 - H8 | 0,3563 | -2,4660 | 0,0154 | 0,6832 | 0,0001 |
| 8 | BCP8 | O7 - H9 | 0,3381 | -2,3177 | 0,0156 | 0,6449 | 0,0001 |
| 9 | BCP9 | H3 - O10 | 0,0334 | 0,1030 | 0,0258 | 0,0021 | 0,0006 |
| 10 | BCP10 | O4 - H12 | 0,0282 | 0,0998 | 0,0560 | -0,0004 | 0,0031 |
| 11 | BCP11 | O10 - H11 | 0,3497 | -2,4127 | 0,0156 | 0,6696 | 0,0001 |
| 12 | BCP12 | O10 - H12 | 0,3462 | -2,3916 | 0,0153 | 0,6635 | 0,0001 |
| 13 | BCP13 | O4 - H14 | 0,0318 | 0,1055 | 0,0361 | 0,0010 | 0,0023 |
| 14 | BCP14 | O13 - H14 | 0,3402 | -2,3492 | 0,0158 | 0,6514 | 0,0001 |
| 15 | BCP15 | O13 - H15 | 0,3580 | -2,4881 | 0,0149 | 0,6873 | 0,0001 |
| 16 | BCP16 | H9 - O16 | 0,0335 | 0,1036 | 0,0269 | 0,0021 | 0,0004 |
| 17 | BCP17 | O16 - C19 | 0,0047 | 0,0177 | 2,6229 | -0,0010 | 0,6406 |
| 18 | BCP18 | O16 - H17 | 0,3582 | -2,4743 | 0,0153 | 0,6854 | 0,0000 |
| 19 | BCP19 | O16 - H18 | 0,3374 | -2,3137 | 0,0162 | 0,6439 | 0,0001 |
| 20 | BCP20 | O1 - C24 | 0,0030 | 0,0107 | 1,0359 | -0,0006 | 0,2257 |
| 21 | BCP21 | H11 - O33 | 0,0237 | 0,0916 | 0,0494 | -0,0019 | 0,0063 |
| 22 | BCP22 | C19 - C20 | 0,3155 | -0,9275 | 0,1839 | 0,3351 | 0,0002 |
| 23 | BCP23 | C20 - C21 | 0,3289 | -0,9876 | 0,2238 | 0,3624 | 0,0001 |
| 24 | BCP24 | C21 - C22 | 0,3145 | -0,9359 | 0,1854 | 0,3343 | 0,0004 |
| 25 | BCP25 | C22 - C23 | 0,3144 | -0,9357 | 0,1845 | 0,3341 | 0,0004 |
| 26 | BCP26 | C19 - C24 | 0,3166 | -0,9347 | 0,1829 | 0,3370 | 0,0001 |
| 27 | BCP27 | C23 - C24 | 0,3295 | -0,9914 | 0,2234 | 0,3638 | 0,0000 |
| 28 | BCP28 | C20 - H25 | 0,2926 | -1,0638 | 0,0084 | 0,3026 | 0,0000 |

| | | | | | | | |
|---|---|---|---|---|---|---|---|
| 29 | BCP29 | C21 - H26 | 0,2888 | -1,0312 | 0,0230 | 0,2971 | 0,0000 |
| 30 | BCP30 | C23 - H27 | 0,2888 | -1,0315 | 0,0228 | 0,2972 | 0,0000 |
| 31 | BCP31 | C24 - H28 | 0,2923 | -1,0624 | 0,0076 | 0,3020 | 0,0000 |
| 32 | BCP32 | C22 - N29 | 0,3291 | -1,0783 | 0,0845 | 0,4920 | 0,0005 |
| 33 | BCP33 | N29 - H30 | 0,3458 | -1,8780 | 0,0415 | 0,5221 | 0,0001 |
| 34 | BCP34 | N29 - H31 | 0,3457 | -1,8783 | 0,0415 | 0,5222 | 0,0001 |
| 35 | BCP35 | H8 - O34 | 0,0140 | 0,0576 | 0,0855 | -0,0029 | 0,0124 |
| 36 | BCP36 | C19 - C32 | 0,2893 | -0,8334 | 0,1613 | 0,2861 | 0,0002 |
| 37 | BCP37 | C32 - O33 | 0,4110 | -0,3386 | 0,0901 | 0,7422 | 0,0003 |
| 38 | BCP38 | O13 - H35 | 0,0477 | 0,1165 | 0,0285 | 0,0097 | 0,0008 |
| 39 | BCP39 | C32 - O34 | 0,3206 | -0,6631 | 0,0276 | 0,5227 | 0,0006 |
| 40 | BCP40 | O34 - H35 | 0,3165 | -2,1468 | 0,0112 | 0,5988 | 0,0006 |

Amino8

| | | | | | | | |
|---|---|---|---|---|---|---|---|
| 1 | BCP1 | O1 - H2 | 0,3559 | -2,4633 | 0,0155 | 0,6827 | 0,0000 |
| 2 | BCP2 | O1 - H3 | 0,3418 | -2,3539 | 0,0155 | 0,6539 | 0,0001 |
| 3 | BCP3 | O4 - H5 | 0,3594 | -2,5057 | 0,0152 | 0,6918 | 0,0000 |
| 4 | BCP4 | O4 - H6 | 0,3382 | -2,3274 | 0,0165 | 0,6465 | 0,0001 |
| 5 | BCP5 | O4 - H9 | 0,0266 | 0,0952 | 0,0474 | -0,0009 | 0,0020 |
| 6 | BCP6 | O7 - H8 | 0,3438 | -2,3688 | 0,0147 | 0,6573 | 0,0001 |
| 7 | BCP7 | O7 - H9 | 0,3444 | -2,3800 | 0,0146 | 0,6595 | 0,0000 |
| 8 | BCP8 | O1 - H12 | 0,0343 | 0,1056 | 0,0521 | 0,0023 | 0,0005 |
| 9 | BCP9 | H11 - C25 | 0,0045 | 0,0173 | 2,1544 | -0,0010 | 0,3893 |
| 10 | BCP10 | O10 - H11 | 0,3582 | -2,4798 | 0,0154 | 0,6864 | 0,0000 |
| 11 | BCP11 | O10 - H12 | 0,3372 | -2,3141 | 0,0161 | 0,6436 | 0,0001 |
| 12 | BCP12 | H6 - O13 | 0,0325 | 0,1010 | 0,0171 | 0,0018 | 0,0009 |
| 13 | BCP13 | O13 - H14 | 0,3493 | -2,4109 | 0,0153 | 0,6693 | 0,0001 |
| 14 | BCP14 | O13 - H15 | 0,3433 | -2,3621 | 0,0151 | 0,6563 | 0,0001 |
| 15 | BCP15 | O10 - H18 | 0,0375 | 0,1105 | 0,0301 | 0,0039 | 0,0003 |
| 16 | BCP16 | H15 - O16 | 0,0304 | 0,1019 | 0,0657 | 0,0005 | 0,0010 |
| 17 | BCP17 | O16 - H18 | 0,3342 | -2,2943 | 0,0162 | 0,6381 | 0,0001 |
| 18 | BCP18 | O16 - H17 | 0,3593 | -2,5041 | 0,0150 | 0,6914 | 0,0000 |
| 19 | BCP19 | H3 - O19 | 0,0322 | 0,1062 | 0,0357 | 0,0011 | 0,0018 |
| 20 | BCP20 | H8 - O19 | 0,0281 | 0,0964 | 0,0423 | -0,0002 | 0,0031 |
| 21 | BCP21 | O4 - H23 | 0,0278 | 0,0960 | 0,0428 | -0,0003 | 0,0032 |
| 22 | BCP22 | O19 - H20 | 0,3325 | -2,2737 | 0,0161 | 0,6334 | 0,0001 |
| 23 | BCP23 | O19 - H21 | 0,3594 | -2,5050 | 0,0145 | 0,6917 | 0,0000 |
| 24 | BCP24 | O16 - H24 | 0,0299 | 0,1011 | 0,0355 | 0,0003 | 0,0010 |
| 25 | BCP25 | H20 - O22 | 0,0391 | 0,1115 | 0,0191 | 0,0048 | 0,0004 |
| 26 | BCP26 | O22 - H23 | 0,3459 | -2,3813 | 0,0150 | 0,6616 | 0,0001 |
| 27 | BCP27 | O22 - H24 | 0,3436 | -2,3650 | 0,0149 | 0,6570 | 0,0001 |
| 28 | BCP28 | C25 - C26 | 0,3154 | -0,9268 | 0,1843 | 0,3349 | 0,0002 |
| 29 | BCP29 | C26 - C27 | 0,3289 | -0,9882 | 0,2239 | 0,3626 | 0,0001 |
| 30 | BCP30 | C27 - C28 | 0,3147 | -0,9371 | 0,1855 | 0,3348 | 0,0004 |
| 31 | BCP31 | C28 - C29 | 0,3145 | -0,9360 | 0,1845 | 0,3342 | 0,0004 |
| 32 | BCP32 | C25 - C30 | 0,3162 | -0,9321 | 0,1841 | 0,3364 | 0,0002 |
| 33 | BCP33 | C29 - C30 | 0,3293 | -0,9901 | 0,2230 | 0,3633 | 0,0001 |
| 34 | BCP34 | C26 - H31 | 0,2923 | -1,0619 | 0,0085 | 0,3022 | 0,0000 |
| 35 | BCP35 | C27 - H32 | 0,2888 | -1,0315 | 0,0228 | 0,2972 | 0,0000 |
| 36 | BCP36 | C29 - H33 | 0,2888 | -1,0316 | 0,0227 | 0,2972 | 0,0000 |

| | | | | | | | |
|---|---|---|---|---|---|---|---|
| 37 | BCP37 | C30 - H34 | 0,2920 | -1,0606 | 0,0081 | 0,3017 | 0,0000 |
| 38 | BCP38 | C28 - N35 | 0,3291 | -1,0781 | 0,0849 | 0,4916 | 0,0005 |
| 39 | BCP39 | N35 - H36 | 0,3458 | -1,8780 | 0,0415 | 0,5221 | 0,0001 |
| 40 | BCP40 | N35 - H37 | 0,3458 | -1,8780 | 0,0415 | 0,5221 | 0,0001 |
| 41 | BCP41 | H2 - O40 | 0,0158 | 0,0639 | 0,0714 | -0,0028 | 0,0075 |
| 42 | BCP42 | O7 - H41 | 0,0496 | 0,1171 | 0,0399 | 0,0107 | 0,0012 |
| 43 | BCP43 | H14 - O39 | 0,0257 | 0,0987 | 0,0665 | -0,0016 | 0,0065 |
| 44 | BCP44 | C25 - C38 | 0,2890 | -0,8316 | 0,1593 | 0,2854 | 0,0002 |
| 45 | BCP45 | C38 - O39 | 0,4117 | -0,3261 | 0,0928 | 0,7435 | 0,0002 |
| 46 | BCP46 | C38 - O40 | 0,3210 | -0,6737 | 0,0311 | 0,5233 | 0,0005 |
| 47 | BCP47 | O40 - H41 | 0,3124 | -2,1054 | 0,0115 | 0,5886 | 0,0006 |

Amino10

| | | | | | | | |
|---|---|---|---|---|---|---|---|
| 1 | BCP1 | O1 - H2 | 0,3415 | -2,3521 | 0,0161 | 0,6534 | 0,0001 |
| 2 | BCP2 | O1 - H3 | 0,3505 | -2,4290 | 0,0151 | 0,6729 | 0,0000 |
| 3 | BCP3 | O4 - H5 | 0,3512 | -2,4232 | 0,0148 | 0,6725 | 0,0000 |
| 4 | BCP4 | O4 - H6 | 0,3359 | -2,2959 | 0,0158 | 0,6398 | 0,0001 |
| 5 | BCP5 | H2 - O7 | 0,0318 | 0,1036 | 0,0433 | 0,0012 | 0,0016 |
| 6 | BCP6 | O7 - H8 | 0,3590 | -2,4998 | 0,0140 | 0,6903 | 0,0000 |
| 7 | BCP7 | O7 - H9 | 0,3314 | -2,2601 | 0,0159 | 0,6301 | 0,0001 |
| 8 | BCP8 | O4 - H12 | 0,0252 | 0,0955 | 0,0425 | -0,0015 | 0,0053 |
| 9 | BCP9 | O4 - H18 | 0,0296 | 0,0976 | 0,0408 | 0,0004 | 0,0004 |
| 10 | BCP10 | O10 - H11 | 0,3451 | -2,3762 | 0,0152 | 0,6601 | 0,0001 |
| 11 | BCP11 | O10 - H12 | 0,3483 | -2,4063 | 0,0151 | 0,6673 | 0,0001 |
| 12 | BCP12 | O10 - H15 | 0,0363 | 0,1072 | 0,0154 | 0,0035 | 0,0003 |
| 13 | BCP13 | O13 - H14 | 0,3594 | -2,5053 | 0,0150 | 0,6918 | 0,0000 |
| 14 | BCP14 | O13 - H15 | 0,3349 | -2,2970 | 0,0164 | 0,6390 | 0,0001 |
| 15 | BCP15 | O1 - H17 | 0,0268 | 0,0911 | 0,0440 | -0,0005 | 0,0007 |
| 16 | BCP16 | H11 - O19 | 0,0285 | 0,0966 | 0,0298 | -0,0001 | 0,0025 |
| 17 | BCP17 | O16 - H17 | 0,3446 | -2,3729 | 0,0148 | 0,6589 | 0,0001 |
| 18 | BCP18 | O16 - H18 | 0,3425 | -2,3546 | 0,0150 | 0,6545 | 0,0001 |
| 19 | BCP19 | O16 - H20 | 0,0390 | 0,1115 | 0,0212 | 0,0047 | 0,0003 |
| 20 | BCP20 | O19 - H21 | 0,3593 | -2,4994 | 0,0140 | 0,6905 | 0,0000 |
| 21 | BCP21 | O19 - H20 | 0,3324 | -2,2682 | 0,0160 | 0,6324 | 0,0001 |
| 22 | BCP22 | H9 - O22 | 0,0388 | 0,1101 | 0,0194 | 0,0047 | 0,0003 |
| 23 | BCP23 | O13 - H24 | 0,0292 | 0,1007 | 0,0626 | 0,0000 | 0,0013 |
| 24 | BCP24 | H23 - O25 | 0,0312 | 0,0986 | 0,0422 | 0,0012 | 0,0006 |
| 25 | BCP25 | O22 - H23 | 0,3416 | -2,3414 | 0,0149 | 0,6518 | 0,0001 |
| 26 | BCP26 | O13 - H30 | 0,0286 | 0,0987 | 0,0234 | -0,0002 | 0,0028 |
| 27 | BCP27 | O22 - H24 | 0,3447 | -2,3749 | 0,0148 | 0,6596 | 0,0001 |
| 28 | BCP28 | O1 - H27 | 0,0255 | 0,0962 | 0,0439 | -0,0015 | 0,0046 |
| 29 | BCP29 | O19 - H26 | 0,0283 | 0,0962 | 0,0272 | -0,0001 | 0,0024 |
| 30 | BCP30 | O25 - H26 | 0,3458 | -2,3790 | 0,0153 | 0,6612 | 0,0001 |
| 31 | BCP31 | O25 - H27 | 0,3485 | -2,4068 | 0,0153 | 0,6677 | 0,0001 |
| 32 | BCP32 | O7 - H29 | 0,0285 | 0,0972 | 0,0328 | -0,0001 | 0,0026 |
| 33 | BCP33 | H6 - O28 | 0,0364 | 0,1076 | 0,0229 | 0,0035 | 0,0003 |
| 34 | BCP34 | O28 - H29 | 0,3456 | -2,3780 | 0,0150 | 0,6608 | 0,0001 |
| 35 | BCP35 | O28 - H30 | 0,3449 | -2,3732 | 0,0152 | 0,6594 | 0,0001 |
| 36 | BCP36 | O1 - C36 | 0,0062 | 0,0253 | 0,1604 | -0,0014 | 0,4111 |
| 37 | BCP37 | C44 - O45 | 0,4151 | -0,3109 | 0,0946 | 0,7518 | 0,0003 |

| | | | | | | | |
|---|---|---|---|---|---|---|---|
| 38 | BCP38 | O16 - C36 | 0,0042 | 0,0152 | 1,1656 | -0,0009 | 0,7189 |
| 39 | BCP39 | C31 - C32 | 0,3167 | -0,9335 | 0,1850 | 0,3374 | 0,0002 |
| 40 | BCP40 | C32 - C33 | 0,3285 | -0,9879 | 0,2180 | 0,3616 | 0,0001 |
| 41 | BCP41 | H5 - N41 | 0,0189 | 0,0590 | 0,0908 | -0,0015 | 0,0069 |
| 42 | BCP42 | C33 - C34 | 0,3172 | -0,9481 | 0,1916 | 0,3396 | 0,0004 |
| 43 | BCP43 | O28 - H39 | 0,0023 | 0,0091 | 0,5047 | -0,0006 | 0,1988 |
| 44 | BCP44 | C34 - C35 | 0,3161 | -0,9431 | 0,1886 | 0,3374 | 0,0005 |
| 45 | BCP45 | C31 - C36 | 0,3168 | -0,9354 | 0,1832 | 0,3374 | 0,0002 |
| 46 | BCP46 | C35 - C36 | 0,3294 | -0,9921 | 0,2199 | 0,3635 | 0,0000 |
| 47 | BCP47 | C32 - H37 | 0,2930 | -1,0676 | 0,0083 | 0,3033 | 0,0000 |
| 48 | BCP48 | C33 - H38 | 0,2891 | -1,0346 | 0,0215 | 0,2977 | 0,0000 |
| 49 | BCP49 | C35 - H39 | 0,2888 | -1,0325 | 0,0212 | 0,2970 | 0,0000 |
| 50 | BCP50 | C36 - H40 | 0,2926 | -1,0645 | 0,0073 | 0,3025 | 0,0000 |
| 51 | BCP51 | C34 - N41 | 0,3217 | -1,0502 | 0,0766 | 0,4597 | 0,0007 |
| 52 | BCP52 | N41 - H42 | 0,3448 | -1,8589 | 0,0382 | 0,5169 | 0,0001 |
| 53 | BCP53 | N41 - H43 | 0,3442 | -1,8508 | 0,0389 | 0,5150 | 0,0001 |
| 54 | BCP54 | H3 - O45 | 0,0216 | 0,0829 | 0,0108 | -0,0022 | 0,0077 |
| 55 | BCP55 | C31 - C44 | 0,2891 | -0,8346 | 0,1539 | 0,2868 | 0,0002 |
| 56 | BCP56 | C44 - O46 | 0,3145 | -0,6635 | 0,0243 | 0,5072 | 0,0007 |
| 57 | BCP57 | O46 - H47 | 0,3518 | -2,4953 | 0,0119 | 0,6821 | 0,0005 |
