# Supplementary material for: Hydration of p - aminobenzoic acid: structures and non-covalent bondings of aminobenzoic acid-water clusters
Source: J Mol Model. 2024 Jan 12;30(2):38. doi: 10.1007/s00894-023-05810-2 (PMC10786749; doi:10.1007/s00894-023-05810-2)
Supplement: Supplementary file 3 — (pdf 332 KB) [file 894_2023_5810_MOESM3_ESM.pdf]

Cartesian coordinates of : ABW1_ 1

Atomic number (AN) and Cartesian coordinates

AN	X	Y	Z
O	4.803903	0.409621	-0.048760
H	5.137937	0.562247	0.840159
H	4.879350	-0.540900	-0.178030
C	0.008504	-0.127538	-0.005951
C	-0.597041	1.128692	-0.009631
C	-1.964782	1.253124	-0.000926
C	-2.783167	0.116276	0.012124
C	-2.174544	-1.146079	0.011770
C	-0.807004	-1.258365	0.003619
H	0.014385	2.016241	-0.021375
H	-2.423443	2.230360	-0.005421
H	-2.795535	-2.029086	0.016894
H	-0.350112	-2.235440	0.002003
N	-4.137128	0.233957	0.066467
H	-4.539888	1.119809	-0.179560
H	-4.685785	-0.571682	-0.173323
C	1.460494	-0.291499	-0.014730
O	2.025518	-1.374388	-0.006485
O	2.140002	0.852542	-0.033687
H	3.112845	0.660898	-0.032812

Cartesian coordinates of : ABW1_ 2

Atomic number (AN) and Cartesian coordinates

AN	X	Y	Z
O	2.924763	2.474859	0.598773
H	2.762879	1.592487	0.233435
H	3.840581	2.661040	0.379886
C	0.243404	-0.517342	-0.110700
C	-0.767400	-1.401698	0.271040
C	-2.075069	-0.992492	0.331171
C	-2.425401	0.325772	0.005354
C	-1.409782	1.213305	-0.379736
C	-0.105794	0.794708	-0.434680
H	-0.519957	-2.418574	0.528473
H	-2.849445	-1.681676	0.631865
H	-1.668791	2.229990	-0.633230
H	0.663878	1.487608	-0.734968
N	-3.720428	0.723108	0.025931
H	-4.392623	0.143784	0.493867
H	-3.922306	1.705357	-0.003676
C	1.637471	-0.921095	-0.177823
O	2.571874	-0.181640	-0.445202
O	1.846696	-2.218646	0.080461

```
  H      2.797552     -2.385295      0.022824
 ---------------------------------------------------

Cartesian coordinates of :    ABW1_ 3

 ---------------------------------------------------
 Atomic number (AN) and Cartesian coordinates
  AN          X            Y            Z
 ---------------------------------------------------
  O      3.705659     -0.102750      1.896962
  H      3.528611     -0.066432      0.943589
  H      2.832865     -0.091495      2.297081
  C     -1.164402      0.040173     -0.097153
  C     -0.492139     -1.163046     -0.303972
  C      0.846225     -1.173442     -0.616430
  C      1.554002      0.025126     -0.732991
  C      0.881046      1.232359     -0.526310
  C     -0.456242      1.234258     -0.214567
  H     -1.023569     -2.096695     -0.220883
  H      1.362251     -2.107944     -0.776178
  H      1.423652      2.161026     -0.616041
  H     -0.970449      2.169321     -0.059416
  N      2.905577      0.014927     -0.981989
  H      3.264432     -0.815987     -1.421786
  H      3.289063      0.865310     -1.359474
  C     -2.586169      0.090734      0.233473
  O     -3.220253      1.111251      0.419447
  O     -3.165532     -1.115304      0.320788
  H     -4.098808     -0.984143      0.537150
 ---------------------------------------------------

Cartesian coordinates of :    ABW1_ 4

 ---------------------------------------------------
 Atomic number (AN) and Cartesian coordinates
  AN          X            Y            Z
 ---------------------------------------------------
  O     -4.619154     -1.580460      0.350252
  H     -4.258670     -1.503969     -0.537169
  H     -3.926487     -1.221283      0.911266
  C      1.266477     -0.033977     -0.021907
  C      0.980607      1.325334      0.112359
  C     -0.312792      1.781170      0.061780
  C     -1.377228      0.887906     -0.123712
  C     -1.088728     -0.477783     -0.262371
  C      0.207384     -0.922003     -0.211533
  H      1.783391      2.030093      0.255430
  H     -0.524481      2.834575      0.165249
  H     -1.899439     -1.175003     -0.410318
  H      0.417498     -1.974215     -0.321276
  N     -2.658545      1.330621     -0.132250
  H     -2.827063      2.312875     -0.246942
  H     -3.378989      0.712054     -0.456787
  C      2.625748     -0.552981      0.026435
```

O	2.931003	-1.725039	-0.096521
O	3.561972	0.389396	0.225964
H	4.424677	-0.046645	0.242422

Cartesian coordinates of : ABW2_ 1

Atomic number (AN) and Cartesian coordinates

AN	X	Y	Z
O	-4.183917	-1.319759	-0.101477
H	-4.422877	-0.381051	0.023452
H	-4.434233	-1.526012	-1.006147
O	-4.364565	1.429596	0.252927
H	-4.485170	1.626512	1.185297
H	-3.401573	1.357183	0.131086
C	0.494781	-0.022825	-0.037855
C	1.233378	-1.197397	0.106106
C	2.605438	-1.167810	0.143861
C	3.294929	0.047128	0.037166
C	2.552876	1.226933	-0.109888
C	1.182100	1.185853	-0.145466
H	0.722841	-2.143098	0.186937
H	3.167970	-2.082514	0.254033
H	3.074251	2.168167	-0.197038
H	0.622329	2.100417	-0.261227
N	4.651148	0.085183	0.115409
H	5.153304	-0.772978	-0.021244
H	5.116829	0.915926	-0.201517
C	-0.964186	-0.024722	-0.084892
O	-1.632414	0.993659	-0.221084
O	-1.514153	-1.223706	0.029667
H	-2.507208	-1.180112	-0.035951

Cartesian coordinates of : ABW2_ 2

Atomic number (AN) and Cartesian coordinates

AN	X	Y	Z
O	2.110022	-2.393170	-0.244121
H	2.321665	-1.491548	-0.528747
H	2.868107	-2.669259	0.277622
O	0.029520	-2.182599	1.707322
H	-0.538812	-1.442061	1.480182
H	0.705636	-2.197737	1.008183
C	0.358043	0.763488	-0.212354
C	-0.516508	1.363082	0.695539
C	-1.854079	1.056881	0.690568
C	-2.372874	0.141541	-0.237249

```
  C     -1.495190     -0.449391     -1.158747
  C     -0.159122     -0.145051     -1.137613
  H     -0.138171      2.066976      1.418711
  H     -2.522099      1.517863      1.401915
  H     -1.885956     -1.148407     -1.882329
  H      0.506406     -0.609594     -1.848138
  N     -3.685597     -0.192409     -0.220606
  H     -4.322065      0.391477      0.289981
  H     -4.065444     -0.678647     -1.011851
  C      1.786315      1.029408     -0.200801
  O      2.615735      0.407119     -0.847374
  O      2.153923      2.035031      0.601855
  H      3.116800      2.117002      0.561199
 ---------------------------------------------------

Cartesian coordinates of :    ABW2_ 3

 ---------------------------------------------------
 Atomic number (AN) and Cartesian coordinates
  AN          X             Y             Z
 ---------------------------------------------------
  O      0.861297      2.467020     -0.359172
  H      0.601204      1.833594     -1.035604
  H      0.380548      2.179507      0.422927
  O      3.532477      1.516675      0.132131
  H      3.435100      0.555615      0.084431
  H      2.635015      1.853571     -0.019800
  C     -1.244519     -0.348302      0.002089
  C     -0.543575     -0.425312      1.204705
  C      0.777973     -0.801846      1.223385
  C      1.441096     -1.117531      0.034146
  C      0.738687     -1.041376     -1.172741
  C     -0.581908     -0.664096     -1.182635
  H     -1.039596     -0.188172      2.131456
  H      1.316180     -0.857818      2.157214
  H      1.245845     -1.283723     -2.094198
  H     -1.118485     -0.609907     -2.116478
  N      2.775556     -1.432165      0.045082
  H      3.144109     -1.780243      0.913889
  H      3.126686     -1.910451     -0.766940
  C     -2.646597      0.055012     -0.057180
  O     -3.302798      0.138407     -1.077116
  O     -3.178532      0.340329      1.140429
  H     -4.101993      0.594447      1.006736
 ---------------------------------------------------

Cartesian coordinates of :    ABW2_ 4

 ---------------------------------------------------
 Atomic number (AN) and Cartesian coordinates
  AN          X             Y             Z
 ---------------------------------------------------
  O     -1.541789     -3.372738      0.529354
  H     -2.151698     -2.635674      0.349327
```

H	-0.693834	-2.952899	0.691039
O	-3.436425	-1.327173	0.018635
H	-2.987955	-0.475890	-0.111534
H	-3.808214	-1.541660	-0.840783
C	0.094153	0.973251	-0.067801
C	1.310515	1.565381	0.277794
C	2.473961	0.838910	0.271371
C	2.466853	-0.517444	-0.085397
C	1.244650	-1.110968	-0.433974
C	0.087805	-0.375559	-0.424416
H	1.339242	2.605164	0.559710
H	3.408767	1.304787	0.543713
H	1.224734	-2.154865	-0.709347
H	-0.842824	-0.847160	-0.695817
N	3.619234	-1.228922	-0.129032
H	4.432318	-0.851573	0.321725
H	3.561812	-2.229251	-0.179480
C	-1.155851	1.713866	-0.060174
O	-2.256628	1.242173	-0.298712
O	-1.021421	3.012626	0.236704
H	-1.899387	3.417750	0.222407

Cartesian coordinates of : ABW2_ 5

Atomic number (AN) and Cartesian coordinates

AN	X	Y	Z
O	-5.220098	-0.652196	0.135909
H	-5.327684	0.200184	0.569288
H	-5.441183	-1.300808	0.810707
O	4.086651	-1.335163	1.702816
H	4.052762	-0.765915	0.917959
H	3.171684	-1.590994	1.843017
C	-0.498329	0.271061	-0.141948
C	0.140166	-0.781058	-0.794770
C	1.497961	-0.754032	-1.010685
C	2.260239	0.334206	-0.580630
C	1.621883	1.389640	0.074887
C	0.265119	1.353650	0.287667
H	-0.433775	-1.626639	-1.136459
H	1.986477	-1.573117	-1.516259
H	2.205991	2.233315	0.409819
H	-0.220655	2.174236	0.791148
N	3.627118	0.334480	-0.739658
H	3.979897	-0.244973	-1.482807
H	4.071749	1.235777	-0.691525
C	-1.943420	0.274226	0.100892
O	-2.533293	1.173414	0.677303
O	-2.577110	-0.796696	-0.364590
H	-3.546008	-0.723460	-0.161271

Cartesian coordinates of : ABW2_ 6

Atomic number (AN) and Cartesian coordinates

AN	X	Y	Z
O	3.219726	-0.265961	1.755671
H	2.945035	0.480817	1.202192
H	2.400771	-0.735289	1.938767
O	4.332226	-1.576921	-0.555144
H	3.936893	-1.166193	0.231465
H	3.746170	-1.332164	-1.275142
C	-1.664952	0.033766	-0.073347
C	-0.628498	-0.844121	-0.386967
C	0.654592	-0.382426	-0.554734
C	0.940855	0.978682	-0.414211
C	-0.097608	1.859616	-0.099686
C	-1.377043	1.389837	0.066656
H	-0.831569	-1.896343	-0.499773
H	1.455731	-1.067545	-0.787322
H	0.117871	2.911646	0.008692
H	-2.174373	2.075376	0.305893
N	2.232933	1.429113	-0.520060
H	2.854796	0.860775	-1.071224
H	2.348748	2.417173	-0.667621
C	-3.040101	-0.420944	0.110837
O	-3.979834	0.299538	0.386706
O	-3.199695	-1.742595	-0.052730
H	-4.133451	-1.950983	0.087169

Cartesian coordinates of : ABW2_ 7

Atomic number (AN) and Cartesian coordinates

AN	X	Y	Z
O	-3.476339	0.060960	1.679517
H	-3.079232	0.669833	1.035194
H	-4.277924	0.501352	1.973961
O	-4.276740	-1.724410	-0.423343
H	-4.001699	-1.141568	0.304285
H	-3.692188	-1.495997	-1.150065
C	1.698241	-0.042398	-0.060474
C	1.411127	1.319969	-0.000917
C	0.132785	1.777038	-0.215819
C	-0.899948	0.880527	-0.498827
C	-0.614276	-0.486547	-0.553667
C	0.665275	-0.935136	-0.338553
H	2.197354	2.025832	0.210529
H	-0.083127	2.833636	-0.172494
H	-1.411395	-1.184566	-0.758811
H	0.879375	-1.990973	-0.385071
N	-2.189598	1.327277	-0.664665

H	-2.290622	2.304014	-0.883546
H	-2.791142	0.731286	-1.209945
C	3.045141	-0.564872	0.154305
O	3.348712	-1.741174	0.109301
O	3.958626	0.381772	0.413690
H	4.813646	-0.052435	0.539014

Cartesian coordinates of : ABW2_ 8

Atomic number (AN) and Cartesian coordinates

AN	X	Y	Z
O	3.459488	-0.009601	-1.679489
H	4.266108	-0.435240	-1.981724
H	3.076217	-0.629336	-1.037129
O	4.235001	1.769517	0.437267
H	3.971606	1.190807	-0.297884
H	3.653336	1.521338	1.159849
C	-1.709080	-0.026830	0.054600
C	-0.698574	0.886550	0.349809
C	0.589731	0.460394	0.566232
C	0.906367	-0.898447	0.494666
C	-0.105110	-1.814834	0.195590
C	-1.390140	-1.381023	-0.019189
H	-0.925455	1.938179	0.410462
H	1.370334	1.172892	0.784927
H	0.134127	-2.865795	0.139390
H	-2.167462	-2.093622	-0.244579
N	2.204991	-1.318882	0.659107
H	2.792070	-0.716985	1.213478
H	2.326669	-2.295935	0.865890
C	-3.089527	0.388738	-0.179227
O	-4.008615	-0.365401	-0.433251
O	-3.278572	1.713448	-0.091830
H	-4.212906	1.894876	-0.262890

Cartesian coordinates of : ABW2_ 9

Atomic number (AN) and Cartesian coordinates

AN	X	Y	Z
O	2.811226	0.858102	2.383430
H	2.776046	0.957893	1.420160
H	2.027343	0.342881	2.590321
O	4.212058	-1.610240	-0.846248
H	4.685798	-1.537683	-1.679077
H	4.874015	-1.424064	-0.174980
C	-1.649987	-0.005982	-0.117328
C	-0.686551	-0.978681	0.147240
C	0.648917	-0.711345	-0.031460

C	1.066335	0.545293	-0.484764
C	0.099425	1.521080	-0.749745
C	-1.232895	1.244895	-0.568954
H	-0.989191	-1.953019	0.494183
H	1.388616	-1.469444	0.174880
H	0.414231	2.492601	-1.099406
H	-1.971958	2.002006	-0.777849
N	2.400374	0.827888	-0.605271
H	3.022265	0.039335	-0.733861
H	2.635056	1.640576	-1.148584
C	-3.076615	-0.255198	0.059121
O	-3.958196	0.548356	-0.178343
O	-3.359668	-1.482550	0.521212
H	-4.319967	-1.556011	0.606034

Cartesian coordinates of : ABW3_ 1

Atomic number (AN) and Cartesian coordinates

AN	X	Y	Z
O	3.508304	-0.686864	-1.971000
H	2.747710	-0.845767	-1.391031
H	3.133190	-0.300950	-2.766765
O	3.864351	1.261251	0.072604
H	3.958412	0.608930	0.787718
H	3.945960	0.723752	-0.733672
O	3.548935	-0.956680	1.837670
H	3.217570	-0.678372	2.695268
H	2.760286	-1.028489	1.277863
C	-0.800892	0.077075	0.014506
C	-1.571067	1.240010	0.063936
C	-2.941720	1.178455	0.056773
C	-3.598913	-0.058784	0.000688
C	-2.824833	-1.227032	-0.050004
C	-1.455829	-1.153644	-0.043235
H	-1.084846	2.200997	0.105301
H	-3.528733	2.083549	0.092613
H	-3.321389	-2.184309	-0.096385
H	-0.870727	-2.058599	-0.084813
N	-4.953211	-0.126991	0.033294
H	-5.476364	0.708452	-0.154105
H	-5.392153	-0.990323	-0.229134
C	0.653968	0.113184	0.015819
O	1.353392	-0.897775	-0.039463
O	1.180294	1.324397	0.079758
H	2.177066	1.279841	0.076636

Cartesian coordinates of : ABW3_ 2

```
 ---------------------------------------------------
 Atomic number (AN) and Cartesian coordinates
  AN         X             Y             Z
 ---------------------------------------------------
  O      3.623734     -1.417029     -0.865008
  H      4.098810     -1.045976     -0.094405
  H      3.769481     -0.782523     -1.572686
  O      3.375477      1.991395      0.390664
  H      2.645749      1.549820     -0.074997
  H      3.894867      2.408819     -0.301310
  O      4.840471     -0.179203      1.316180
  H      4.412167     -0.504691      2.111998
  H      4.353722      0.634983      1.079572
  C     -0.961602     -0.091512     -0.231397
  C     -1.672847     -1.214720      0.191539
  C     -3.024157     -1.147616      0.423268
  C     -3.719930      0.054561      0.238133
  C     -3.004829      1.183634     -0.184682
  C     -1.654501      1.105181     -0.412859
  H     -1.156340     -2.148754      0.341476
  H     -3.565709     -2.021668      0.752082
  H     -3.530544      2.115694     -0.326945
  H     -1.114018      1.981077     -0.735329
  N     -5.064112      0.114344      0.425616
  H     -5.500358     -0.625325      0.945017
  H     -5.487072      1.019924      0.516411
  C      0.474603     -0.133249     -0.484010
  O      1.120572      0.835745     -0.868703
  O      1.039218     -1.310733     -0.268913
  H      2.011827     -1.280868     -0.493913
 ---------------------------------------------------

Cartesian coordinates of :    ABW3_ 3

 ---------------------------------------------------
 Atomic number (AN) and Cartesian coordinates
  AN         X             Y             Z
 ---------------------------------------------------
  O      4.744854     -1.623519      0.049203
  H      3.801496     -1.457904     -0.122823
  H      4.765754     -2.131094      0.864428
  O     -4.517955     -1.156086      1.926773
  H     -4.511417     -0.625482      1.114839
  H     -3.603839     -1.431116      2.031838
  O      4.676717      1.102146      0.597089
  H      5.057661      1.576399     -0.146935
  H      4.882160      0.161473      0.435444
  C     -0.008487      0.189224     -0.280711
  C     -0.706618      1.337777      0.086410
  C     -2.073053      1.402878     -0.049173
  C     -2.784665      0.315573     -0.560587
  C     -2.086531     -0.837625     -0.928718
  C     -0.721094     -0.893850     -0.790147
  H     -0.171645      2.186828      0.478929
```

```
  H     -2.608214      2.295936      0.235893
  H     -2.631975     -1.681133     -1.323913
  H     -0.188580     -1.785742     -1.079379
  N     -4.157288      0.351291     -0.638172
  H     -4.571967      1.267929     -0.643496
  H     -4.573441     -0.279588     -1.302519
  C      1.446638      0.092189     -0.150868
  O      2.080790     -0.902221     -0.478875
  O      2.021274      1.171438      0.351066
  H      3.012445      1.073392      0.405613
 ---------------------------------------------------

Cartesian coordinates of :    ABW3_ 4

 ---------------------------------------------------
 Atomic number (AN) and Cartesian coordinates
  AN          X             Y             Z
 ---------------------------------------------------
  O      0.548713      3.292672     -0.540928
  H     -0.389538      3.064988     -0.436165
  H      0.609389      4.213528     -0.274268
  O     -1.986499      2.248140      0.337240
  H     -2.271507      1.489143     -0.189066
  H     -1.372456      1.874702      0.986419
  O      0.458394      1.693065      1.913183
  H      0.739452      2.169331      1.116624
  H      0.722637      0.781520      1.757802
  C     -0.548456     -1.033078     -0.255599
  C      0.223304     -1.842377      0.580215
  C      1.591069     -1.731427      0.600307
  C      2.243660     -0.806605     -0.228148
  C      1.467515     -0.000678     -1.073857
  C      0.101386     -0.112148     -1.078986
  H     -0.257671     -2.560352      1.224198
  H      2.179136     -2.357379      1.253987
  H      1.959001      0.714500     -1.715403
  H     -0.484286      0.517861     -1.729884
  N      3.591315     -0.666059     -0.183520
  H      4.131778     -1.390535      0.252270
  H      4.041530     -0.165873     -0.927909
  C     -1.999628     -1.110064     -0.283011
  O     -2.727790     -0.339386     -0.889124
  O     -2.510926     -2.119318      0.433263
  H     -3.474909     -2.072142      0.371432
 ---------------------------------------------------

Cartesian coordinates of :    ABW3_ 5

 ---------------------------------------------------
 Atomic number (AN) and Cartesian coordinates
  AN          X             Y             Z
 ---------------------------------------------------
  O      0.795771      3.264923      0.466583
  H      0.909080      2.754932     -0.349511
```

H	0.788968	4.182747	0.182679
O	-1.896930	2.333892	-0.227827
H	-1.125750	2.674730	0.248488
H	-2.173082	1.546701	0.262273
O	0.346764	1.627178	-1.953247
H	0.678032	0.744329	-1.763991
H	-0.507719	1.673314	-1.495366
C	-0.581406	-1.016209	0.262905
C	0.164352	-1.856970	-0.565509
C	1.534518	-1.785957	-0.590174
C	2.215820	-0.873790	0.229332
C	1.465746	-0.034307	1.066102
C	0.097077	-0.104401	1.073738
H	-0.339395	-2.562756	-1.205541
H	2.102279	-2.432859	-1.241337
H	1.979631	0.674492	1.697002
H	-0.468590	0.553523	1.714823
N	3.569769	-0.829304	0.240045
H	4.069554	-1.277474	-0.505615
H	4.018956	-0.038825	0.664497
C	-2.034056	-1.041942	0.282860
O	-2.738514	-0.230877	0.864728
O	-2.576363	-2.050033	-0.410877
H	-3.538477	-1.966934	-0.359116

Cartesian coordinates of : ABW3_ 6

Atomic number (AN) and Cartesian coordinates

AN	X	Y	Z
O	-0.223360	2.585571	0.405665
H	-0.938448	1.952127	0.296194
H	0.413008	2.139573	0.986379
O	2.202336	1.424161	1.484002
H	2.135915	0.484147	1.678754
H	2.349684	1.462981	0.526435
O	1.936889	1.941574	-1.398446
H	1.101925	2.231473	-0.997427
H	2.412803	2.753965	-1.588728
C	0.118242	-1.004325	-0.115882
C	-0.552546	-0.917439	1.104720
C	-1.868105	-0.529075	1.159072
C	-2.565193	-0.206415	-0.014601
C	-1.882453	-0.271931	-1.238736
C	-0.569558	-0.666106	-1.281393
H	-0.035455	-1.159584	2.018960
H	-2.379790	-0.466825	2.107309
H	-2.405581	-0.011314	-2.146100
H	-0.055011	-0.715188	-2.228033
N	-3.877519	0.131682	0.028461
H	-4.277467	0.382680	0.913743
H	-4.284113	0.566046	-0.779609

```
  C      1.506657     -1.432552     -0.211451
  O      2.132801     -1.554749     -1.246683
  O      2.075618     -1.696698      0.978286
  H      2.988623     -1.973677      0.819935
 ---------------------------------------------------
```

Cartesian coordinates of :   ABW3_ 7

```
 ---------------------------------------------------
 Atomic number (AN) and Cartesian coordinates
  AN          X             Y             Z
 ---------------------------------------------------
  O     -0.328488      1.630630     -1.954496
  H      0.524785      1.666940     -1.493582
  H     -0.669877      0.751083     -1.767903
  O      1.917141      2.320787     -0.222526
  H      1.146574      2.668304      0.250030
  H      2.184342      1.531555      0.269142
  O     -0.769104      3.271773      0.463720
  H     -0.885936      2.763258     -0.352833
  H     -0.758128      4.189874      0.180857
  C      0.572448     -1.018879      0.261027
  C     -0.178749     -1.855925     -0.566138
  C     -1.548312     -1.774150     -0.592560
  C     -2.223268     -0.853190      0.222130
  C     -1.468065     -0.021302      1.061590
  C     -0.099927     -0.102331      1.071466
  H      0.319946     -2.570625     -1.200192
  H     -2.120756     -2.420808     -1.239905
  H     -1.977470      0.689073      1.694366
  H      0.469795      0.547363      1.717324
  N     -3.573425     -0.742957      0.172455
  H     -4.096315     -1.484718     -0.255712
  H     -4.036608     -0.244730      0.910205
  C      2.024876     -1.057426      0.284910
  O      2.734581     -0.253313      0.869914
  O      2.560435     -2.068948     -0.409020
  H      3.523080     -1.994091     -0.354277
 ---------------------------------------------------
```

Cartesian coordinates of :   ABW3_ 8

```
 ---------------------------------------------------
 Atomic number (AN) and Cartesian coordinates
  AN          X             Y             Z
 ---------------------------------------------------
  O      3.444153     -0.252238      0.962555
  H      3.166505      0.543523      0.480310
  H      2.633843     -0.563974      1.392242
  O      0.963471     -1.700138      1.590956
  H      1.241817     -2.030827      0.723032
  H      0.228840     -1.108686      1.405062
  O      2.484650     -2.319754     -0.826203
  H      2.958177     -3.134120     -0.638003
```

```
  H      2.991099     -1.627201     -0.371362
  C     -1.572192      0.318811     -0.138330
  C     -0.752318     -0.060155     -1.200395
  C      0.490223      0.504451     -1.362305
  C      0.952804      1.466670     -0.461589
  C      0.134195      1.844111      0.606409
  C     -1.108139      1.278971      0.759487
  H     -1.093089     -0.800777     -1.904878
  H      1.120485      0.207903     -2.186540
  H      0.488256      2.585846      1.305973
  H     -1.736662      1.577411      1.583222
  N      2.219237      1.985188     -0.585048
  H      2.627625      1.932668     -1.503154
  H      2.380694      2.864249     -0.123141
  C     -2.896393     -0.262096      0.070770
  O     -3.651167      0.040182      0.974273
  O     -3.230742     -1.189222     -0.837896
  H     -4.114262     -1.517552     -0.621191
 ---------------------------------------------------

Cartesian coordinates of :    ABW3_ 9

 ---------------------------------------------------
 Atomic number (AN) and Cartesian coordinates
  AN          X             Y             Z
 ---------------------------------------------------
  O      3.770752      0.563616     -1.775218
  H      2.909861      0.949170     -1.960875
  H      3.577136     -0.173541     -1.174111
  O     -5.522689      1.330596     -0.207291
  H     -5.944443      1.506866      0.638977
  H     -5.778380      0.428820     -0.425372
  O      4.724838      2.091583      0.471905
  H      4.415634      1.605661     -0.310346
  H      4.820160      1.418296      1.149848
  C     -1.055035     -0.409880      0.113651
  C     -0.170726      0.601596      0.482260
  C      1.170373      0.340160      0.636866
  C      1.667496     -0.948277      0.426360
  C      0.782753     -1.963167      0.055179
  C     -0.555702     -1.692787     -0.096158
  H     -0.539324      1.600316      0.649757
  H      1.852695      1.128550      0.917137
  H      1.161749     -2.960630     -0.107764
  H     -1.234547     -2.481509     -0.378931
  N      3.017990     -1.194369      0.519519
  H      3.531308     -0.572803      1.122872
  H      3.280409     -2.159757      0.625106
  C     -2.489901     -0.167765     -0.056204
  O     -3.291659     -1.034953     -0.362323
  O     -2.860279      1.091639      0.150616
  H     -3.841435      1.172005      0.023841
 ---------------------------------------------------
```

Cartesian coordinates of : ABW3_10

Atomic number (AN) and Cartesian coordinates

AN	X	Y	Z
O	3.035920	1.007399	-1.448844
H	3.616035	1.726059	-1.713632
H	2.216049	1.443369	-1.169471
O	3.078858	0.861189	1.475262
H	3.289604	0.795798	0.530961
H	3.628432	1.579950	1.798685
O	0.880333	2.209687	0.119585
H	1.433094	1.764934	0.780620
H	0.084367	1.672586	0.063680
C	-1.607992	-0.318113	0.023039
C	-0.968309	-0.538125	-1.198187
C	0.295491	-1.072432	-1.243104
C	0.968996	-1.408653	-0.061613
C	0.328678	-1.181433	1.164274
C	-0.934715	-0.649285	1.199980
H	-1.467753	-0.282597	-2.118294
H	0.786147	-1.233587	-2.190885
H	0.845168	-1.427446	2.079463
H	-1.418049	-0.475478	2.148260
N	2.200060	-1.987396	-0.103408
H	2.725283	-1.871929	-0.952142
H	2.750399	-1.940297	0.736122
C	-2.945838	0.254151	0.110107
O	-3.549085	0.460871	1.146228
O	-3.488618	0.550681	-1.081197
H	-4.366332	0.925139	-0.924763

Cartesian coordinates of : ABW3_11

Atomic number (AN) and Cartesian coordinates

AN	X	Y	Z
O	-2.028832	2.329194	0.842160
H	-2.504079	2.929271	1.422259
H	-2.664309	2.105511	0.144253
O	-3.182649	-0.358023	1.481526
H	-2.611849	0.423011	1.483786
H	-2.713525	-1.005900	0.931401
O	-3.962759	1.063355	-0.918788
H	-3.954367	0.476965	-0.146193
H	-4.825604	1.485100	-0.903198
C	1.892728	-0.122437	-0.107490
C	0.852094	0.476808	-0.814690
C	-0.349242	-0.170576	-0.976289
C	-0.546835	-1.440493	-0.429272
C	0.496822	-2.044255	0.277403

C	1.694988	-1.391931	0.432006
H	0.986064	1.457595	-1.240333
H	-1.153316	0.300179	-1.521019
H	0.349364	-3.026546	0.699706
H	2.497458	-1.863419	0.976636
N	-1.771337	-2.055047	-0.527016
H	-2.357202	-1.750935	-1.286911
H	-1.780471	-3.050252	-0.381197
C	3.182324	0.534877	0.086000
O	4.122465	0.055539	0.689668
O	3.258592	1.752825	-0.470117
H	4.139389	2.109669	-0.291674

Cartesian coordinates of : ABW4_ 1

Atomic number (AN) and Cartesian coordinates

AN	X	Y	Z
O	-3.687503	1.346967	-0.736042
H	-2.911828	1.637574	-0.217705
H	-4.372244	1.992604	-0.544464
O	-4.228515	-1.076002	0.499523
H	-4.138794	-0.223809	0.024522
H	-4.420257	-1.724710	-0.183262
O	0.907341	2.562048	-0.597105
H	1.550401	2.133885	-0.022109
H	0.863635	1.983685	-1.364770
O	-1.482702	2.060777	0.833391
H	-0.661006	2.202227	0.328267
H	-1.344907	1.233115	1.303758
C	0.494959	-0.842559	-0.047635
C	1.080034	-0.506576	1.172719
C	2.417100	-0.200592	1.254030
C	3.224169	-0.223217	0.108290
C	2.634942	-0.561597	-1.117904
C	1.297868	-0.862510	-1.187191
H	0.475905	-0.478853	2.065248
H	2.860176	0.060151	2.203258
H	3.247338	-0.584096	-2.006526
H	0.855158	-1.119835	-2.136488
N	4.537880	0.118539	0.176155
H	4.976577	0.117501	1.078948
H	5.128329	-0.136330	-0.594411
C	-0.931802	-1.131235	-0.173090
O	-1.495258	-1.331477	-1.237360
O	-1.589880	-1.152093	0.983917
H	-2.565126	-1.234923	0.806741

Cartesian coordinates of : ABW4_ 2

```
 ---------------------------------------------------
 Atomic number (AN) and Cartesian coordinates
  AN         X             Y            Z
 ---------------------------------------------------
  O      0.477986      1.896329      1.670602
  H      0.395044      2.286269      0.779068
  H      1.067985      1.144526      1.564502
  O     -2.107903      0.858682      1.853868
  H     -2.595053      1.426930      2.455648
  H     -1.191296      1.197102      1.868417
  O     -0.026720      3.023135     -0.854409
  H     -0.885824      2.562617     -0.931135
  H     -0.250414      3.946436     -0.712394
  O     -2.444405      1.627504     -0.840907
  H     -2.416915      1.391062      0.105244
  H     -2.350154      0.776769     -1.287584
  C     -0.089643     -1.209063     -0.310323
  C      0.720790     -1.625253      0.747003
  C      2.032547     -1.228171      0.827175
  C      2.586102     -0.392428     -0.154636
  C      1.767726      0.030991     -1.211985
  C      0.459229     -0.371461     -1.282077
  H      0.316982     -2.269771      1.510500
  H      2.653319     -1.557379      1.646457
  H      2.181350      0.680463     -1.968265
  H     -0.159239     -0.039588     -2.101202
  N      3.869790      0.030987     -0.061173
  H      4.486298     -0.442390      0.573113
  H      4.295623      0.448911     -0.867810
  C     -1.477732     -1.625606     -0.433845
  O     -2.231791     -1.285896     -1.330941
  O     -1.893090     -2.443282      0.542260
  H     -2.822962     -2.654694      0.382004
 ---------------------------------------------------

Cartesian coordinates of :    ABW4_ 3

 ---------------------------------------------------
 Atomic number (AN) and Cartesian coordinates
  AN         X             Y            Z
 ---------------------------------------------------
  O     -3.117438     -2.002450      0.132454
  H     -3.356687     -1.285523      0.752581
  H     -3.235324     -2.822038      0.620340
  O     -3.377493      0.297417      1.689245
  H     -4.005392      0.825294      1.166082
  H     -2.515805      0.492610      1.292788
  O     -5.013582      1.722871     -0.165809
  H     -4.413790      1.308308     -0.813804
  H     -5.840357      1.241045     -0.248052
  O     -3.073768      0.425050     -1.740777
  H     -2.341584      0.489531     -1.105876
  H     -3.366581     -0.487631     -1.656625
```

C	1.343518	-0.140880	0.012685
C	2.244115	-1.197028	-0.132657
C	3.597734	-0.973015	-0.137262
C	4.105461	0.325798	0.006567
C	3.200272	1.387128	0.149857
C	1.849463	1.151960	0.151628
H	1.873851	-2.203010	-0.244737
H	4.286846	-1.795849	-0.252147
H	3.580946	2.391448	0.257361
H	1.162014	1.975753	0.260603
N	5.443504	0.546632	0.045309
H	6.054022	-0.179276	-0.282065
H	5.773214	1.486757	-0.074455
C	-0.097003	-0.349461	0.024344
O	-0.908841	0.565023	0.153973
O	-0.478007	-1.607377	-0.117863
H	-1.468241	-1.695139	-0.049922

Cartesian coordinates of : ABW4_ 4

Atomic number (AN) and Cartesian coordinates

AN	X	Y	Z
O	-0.720125	0.965606	2.137478
H	-0.257887	1.603889	1.561800
H	-0.856085	1.428587	2.967927
O	0.438478	2.787550	0.325937
H	-0.322176	2.674072	-0.274998
H	1.172626	2.338380	-0.105017
O	-1.935146	2.435031	-1.155274
H	-2.396751	1.812212	-0.563570
H	-2.419258	3.260768	-1.074170
O	-3.123352	0.677556	0.743726
H	-3.150259	-0.237327	0.439556
H	-2.312009	0.731446	1.285464
C	1.387408	-0.727068	-0.254729
C	0.460378	-0.226408	-1.168997
C	-0.799285	-0.762722	-1.254175
C	-1.184105	-1.808830	-0.409272
C	-0.247452	-2.329270	0.490109
C	1.015975	-1.796203	0.558539
H	0.729652	0.593378	-1.815014
H	-1.514666	-0.366944	-1.958395
H	-0.533155	-3.145580	1.135843
H	1.729353	-2.195397	1.261970
N	-2.473989	-2.261442	-0.423131
H	-3.001490	-2.100025	-1.263507
H	-2.651080	-3.146982	0.017519
C	2.714255	-0.144185	-0.098583
O	3.595777	-0.590989	0.609289
O	2.896958	0.981705	-0.809089
H	3.797338	1.296054	-0.647372

Cartesian coordinates of : ABW4_ 5

Atomic number (AN) and Cartesian coordinates

AN	X	Y	Z
O	-2.570056	-1.944099	-1.874568
H	-1.885659	-1.914081	-1.193357
H	-3.102573	-1.166141	-1.673855
O	-3.501326	0.384008	-0.313614
H	-3.625072	-0.283394	0.382955
H	-3.992354	1.179317	-0.032308
O	-4.871672	2.682532	0.474697
H	-4.605472	3.394276	-0.114052
H	-5.806236	2.552408	0.291301
O	-3.213822	-1.775869	1.473469
H	-3.601089	-2.509046	0.987695
H	-2.314006	-1.694519	1.116322
C	1.296470	-0.033667	0.031810
C	1.845162	1.215934	-0.259467
C	3.204031	1.407982	-0.253409
C	4.071794	0.348285	0.044486
C	3.520257	-0.906328	0.340100
C	2.160840	-1.086404	0.332697
H	1.195129	2.044009	-0.490458
H	3.619056	2.378827	-0.478036
H	4.179695	-1.728112	0.574464
H	1.747813	-2.055632	0.563014
N	5.417254	0.522347	0.009172
H	5.774882	1.459816	0.022568
H	5.996819	-0.175300	0.438442
C	-0.141846	-0.266907	0.032092
O	-0.643950	-1.356853	0.299976
O	-0.873048	0.786268	-0.284801
H	-1.850962	0.579866	-0.270021

Cartesian coordinates of : ABW4_ 6

Atomic number (AN) and Cartesian coordinates

AN	X	Y	Z
O	-3.238205	-1.806749	-1.400375
H	-2.574189	-1.655202	-2.078248
H	-2.732425	-2.087968	-0.612760
O	-1.865600	-2.422546	0.957679
H	-2.374451	-3.064098	1.459780
H	-2.031214	-1.566539	1.396857
O	-2.462039	0.114954	2.029107
H	-1.637435	0.608506	2.017457
H	-2.939619	0.409569	1.230382

O	-3.793996	0.732233	-0.366042
H	-3.664533	-0.141266	-0.784918
H	-4.742111	0.825315	-0.244940
C	2.121503	0.051168	-0.140265
C	2.201941	1.413412	0.152320
C	1.092164	2.217299	0.075944
C	-0.149338	1.686107	-0.301335
C	-0.227677	0.316804	-0.599299
C	0.885967	-0.477953	-0.515531
H	3.145255	1.842741	0.448001
H	1.163683	3.268648	0.310179
H	-1.178688	-0.103869	-0.888439
H	0.810735	-1.530557	-0.739226
N	-1.245190	2.477903	-0.409202
H	-1.207661	3.389872	0.007911
H	-2.149096	2.033373	-0.434362
C	3.276228	-0.831180	-0.062184
O	3.259804	-2.027080	-0.290157
O	4.413215	-0.210314	0.294520
H	5.117928	-0.871772	0.320975

Cartesian coordinates of : ABW4_ 7

Atomic number (AN) and Cartesian coordinates

AN	X	Y	Z
O	2.661372	1.929736	0.859890
H	3.353557	1.307802	0.564491
H	3.059549	2.428732	1.577501
O	2.624375	-1.976449	0.811606
H	2.009612	-1.360879	1.255355
H	2.979458	-2.525888	1.514856
O	1.010221	-0.020197	2.031847
H	1.506238	0.715226	1.623039
H	0.161308	-0.027494	1.580319
O	4.496843	-0.042109	0.058986
H	4.533693	-0.040539	-0.901022
H	3.891803	-0.774720	0.287255
C	-2.130590	-0.028949	-0.263156
C	-1.503748	1.194298	-0.504801
C	-0.244654	1.243092	-1.050126
C	0.438525	0.062963	-1.370767
C	-0.196482	-1.164612	-1.140197
C	-1.453934	-1.203533	-0.593779
H	-2.007978	2.113591	-0.255728
H	0.239645	2.192454	-1.221122
H	0.325489	-2.078687	-1.378726
H	-1.929625	-2.153602	-0.408607
N	1.680895	0.107772	-1.926486
H	2.204343	0.956395	-1.799548
H	2.234330	-0.729220	-1.867260
C	-3.459765	-0.120346	0.327163

O	-4.053489	-1.158689	0.551113
O	-4.007424	1.069645	0.621562
H	-4.878974	0.909446	1.008539

Cartesian coordinates of : ABW4_ 8

Atomic number (AN) and Cartesian coordinates

AN	X	Y	Z
O	1.413918	2.261430	-1.098171
H	0.509150	2.261111	-0.733139
H	1.595748	3.180355	-1.310355
O	-0.965060	2.236027	0.342345
H	-1.854022	2.011753	0.009952
H	-0.732290	1.514588	0.934517
O	-3.576504	1.629922	-0.529052
H	-3.637608	0.737847	-0.149947
H	-3.519522	1.483765	-1.477455
O	2.219551	1.836358	1.650640
H	2.003924	1.997780	0.717446
H	2.520312	0.921427	1.658717
C	0.823474	-1.040339	-0.097066
C	-0.028464	-1.049998	-1.200259
C	-1.389931	-1.121899	-1.032731
C	-1.942363	-1.187955	0.249197
C	-1.089969	-1.171818	1.356835
C	0.270926	-1.098029	1.180820
H	0.380938	-0.999105	-2.195610
H	-2.045961	-1.124122	-1.889912
H	-1.511480	-1.222066	2.349101
H	0.924469	-1.090603	2.038655
N	-3.306838	-1.197198	0.412815
H	-3.836297	-1.580309	-0.352845
H	-3.639806	-1.491472	1.315397
C	2.274554	-0.971357	-0.239137
O	3.059474	-0.903703	0.689767
O	2.700532	-0.990755	-1.506837
H	3.665665	-0.926416	-1.499715

Cartesian coordinates of : ABW4_ 9

Atomic number (AN) and Cartesian coordinates

AN	X	Y	Z
O	4.471127	-0.223786	-1.533803
H	3.526402	-0.376568	-1.376830
H	4.881633	-1.079955	-1.384037
O	3.539897	-2.116585	1.057392
H	4.310545	-1.552629	1.159746
H	2.939095	-1.592636	0.509715

O	-4.760511	-1.838675	1.325188
H	-4.816032	-1.045233	0.771597
H	-3.835180	-2.091109	1.273952
O	4.279603	1.076157	0.937077
H	4.520997	0.659351	0.088113
H	4.576558	1.987687	0.866417
C	-0.379123	0.367438	-0.168274
C	-1.117383	1.320807	0.530875
C	-2.483182	1.388712	0.395831
C	-3.154647	0.501047	-0.448689
C	-2.415696	-0.455439	-1.151274
C	-1.051302	-0.516067	-1.010438
H	-0.614016	2.014566	1.183977
H	-3.049266	2.129519	0.939711
H	-2.929651	-1.143623	-1.804921
H	-0.487357	-1.255075	-1.556705
N	-4.524905	0.519890	-0.536787
H	-4.976101	1.370449	-0.245654
H	-4.919207	0.117747	-1.370663
C	1.073427	0.271803	-0.045443
O	1.742291	-0.557341	-0.655968
O	1.615606	1.146582	0.779870
H	2.609234	1.057649	0.809520

Cartesian coordinates of :   ABW4_10

Atomic number (AN) and Cartesian coordinates

AN	X	Y	Z
O	-6.246870	-1.172462	-0.314876
H	-7.061757	-0.692017	-0.480786
H	-5.883067	-0.783497	0.503645
O	-4.995041	-0.002076	1.903374
H	-4.218741	0.307040	1.403711
H	-4.662951	-0.701490	2.471899
O	-4.254234	-0.154445	-1.975761
H	-4.986246	-0.556450	-1.467214
H	-3.772740	-0.894300	-2.354403
O	-2.878621	0.788312	0.195937
H	-2.779409	1.742078	0.121416
H	-3.323811	0.498387	-0.631423
C	1.901303	0.294530	0.036022
C	2.239135	-1.050107	0.187312
C	3.551637	-1.454499	0.168814
C	4.582018	-0.521045	-0.001413
C	4.242248	0.829490	-0.157086
C	2.927648	1.222034	-0.137470
H	1.461524	-1.784982	0.317876
H	3.802473	-2.498065	0.284635
H	5.028344	1.556731	-0.293488
H	2.678540	2.264231	-0.260206
N	5.885031	-0.910537	0.025915

H	6.084264	-1.884946	-0.110059
H	6.569717	-0.274921	-0.341118
C	0.515079	0.757243	0.048913
O	0.185237	1.925303	-0.089313
O	-0.384145	-0.206689	0.226987
H	-1.296371	0.186551	0.212768

Cartesian coordinates of : ABW4_11

Atomic number (AN) and Cartesian coordinates

AN	X	Y	Z
O	3.865492	-1.062108	0.639390
H	3.417187	-1.653869	0.018289
H	3.781761	-0.179877	0.244206
O	1.254820	-1.409404	1.829034
H	0.699284	-0.646268	1.645244
H	2.153879	-1.122071	1.600023
O	-5.401247	-0.916360	-0.207402
H	-5.474308	-1.760647	0.247466
H	-5.611463	-0.257987	0.462140
O	1.861260	-2.862856	-0.628539
H	2.094470	-3.746908	-0.333943
H	1.431467	-2.454447	0.139050
C	-0.874009	0.729351	-0.132961
C	-0.143849	0.117459	-1.149870
C	1.185983	0.412728	-1.334638
C	1.829902	1.329135	-0.500430
C	1.099691	1.945200	0.519188
C	-0.231229	1.648314	0.693973
H	-0.623704	-0.596667	-1.798584
H	1.746728	-0.068301	-2.121513
H	1.591059	2.655615	1.166312
H	-0.790370	2.128661	1.481032
N	3.178024	1.563883	-0.639237
H	3.557010	1.402640	-1.557543
H	3.523406	2.404529	-0.207675
C	-2.290954	0.431156	0.094639
O	-2.958683	0.944154	0.977085
O	-2.801568	-0.457917	-0.749581
H	-3.756368	-0.615721	-0.529155

Cartesian coordinates of : ABW4_12

Atomic number (AN) and Cartesian coordinates

AN	X	Y	Z
O	-5.014387	2.044599	-0.116594
H	-5.255879	2.210776	-1.031305
H	-4.081428	1.768313	-0.146521

```
  O      4.262058      0.550386      1.847523
  H      4.081838     -0.209971      1.274447
  H      3.394472      0.926760      2.020574
  O      5.264080      2.003121     -0.431988
  H      4.897012      1.544763      0.341785
  H      4.647093      1.811504     -1.142132
  O     -5.337315     -0.659859      0.424074
  H     -5.405021      0.301675      0.266927
  H     -5.567081     -0.783964      1.348933
  C     -0.528517     -0.390836     -0.169117
  C     -0.050444     -1.697273     -0.089718
  C      1.291511     -1.964146     -0.220365
  C      2.200913     -0.926396     -0.437855
  C      1.722544      0.385000     -0.522038
  C      0.380200      0.642324     -0.387874
  H     -0.738750     -2.509817      0.075020
  H      1.654674     -2.978610     -0.157275
  H      2.421476      1.191484     -0.685900
  H      0.018778      1.656190     -0.453456
  N      3.548687     -1.179273     -0.503999
  H      3.812716     -2.130388     -0.696005
  H      4.103998     -0.496179     -0.992315
  C     -1.951011     -0.075987     -0.029341
  O     -2.387875      1.066982     -0.068702
  O     -2.727798     -1.131338      0.144644
  H     -3.685983     -0.874869      0.251420
 ---------------------------------------------------

Cartesian coordinates of :    ABW4_13

 ---------------------------------------------------
 Atomic number (AN) and Cartesian coordinates
  AN          X             Y             Z
 ---------------------------------------------------
  O     -4.354419     -0.682581     -1.733930
  H     -3.600699     -1.012931     -2.229304
  H     -4.293888     -1.130080     -0.868999
  O     -3.311688      0.403375      2.203607
  H     -2.467838      0.191549      2.611883
  H     -3.077428      0.900647      1.399823
  O     -4.165959     -1.874972      0.806391
  H     -3.887652     -1.113789      1.350245
  H     -5.060306     -2.074640      1.094049
  O      6.158140     -0.522007      0.256049
  H      6.453822     -0.524183      1.171202
  H      6.021629     -1.449790      0.040421
  C      1.406123      0.140307     -0.187509
  C      1.114850      1.497205     -0.065106
  C     -0.180007      1.949632     -0.161478
  C     -1.228253      1.053932     -0.384484
  C     -0.937448     -0.308431     -0.507817
  C      0.359415     -0.750929     -0.411407
  H      1.911807      2.202560      0.104253
  H     -0.398550      3.002560     -0.067368
```

```
  H     -1.741964    -1.006792    -0.680316
  H      0.576174    -1.802754    -0.509857
  N     -2.531651     1.490363    -0.417797
  H     -2.658493     2.472055    -0.597446
  H     -3.181535     0.893972    -0.913591
  C      2.774194    -0.374016    -0.090985
  O      3.065857    -1.553714    -0.201703
  O      3.693531     0.559477     0.130621
  H      4.589559     0.136229     0.184023
 ---------------------------------------------------
```

Cartesian coordinates of :   ABW4_14

```
 ---------------------------------------------------
 Atomic number (AN) and Cartesian coordinates
  AN          X             Y             Z
 ---------------------------------------------------
  O     -1.254572     1.606909    -1.628777
  H     -2.107322     1.144693    -1.586841
  H     -0.604325     0.942690    -1.382597
  O     -2.532174     2.654000     0.767372
  H     -2.857651     3.535159     0.566313
  H     -1.916691     2.447551     0.046797
  O     -3.929585     0.719856    -0.959661
  H     -3.759929    -0.157049    -0.579643
  H     -3.701619     1.340673    -0.252105
  O      5.563623     0.516108    -0.485725
  H      5.574156     0.986260     0.353812
  H      5.656036     1.203606    -1.152035
  C      0.934315    -0.537510     0.328301
  C      0.487672    -1.431752    -0.643276
  C     -0.813254    -1.879044    -0.642136
  C     -1.707696    -1.446982     0.339357
  C     -1.260154    -0.554337     1.316591
  C      0.039114    -0.108520     1.305730
  H      1.166020    -1.777020    -1.405915
  H     -1.153512    -2.569252    -1.399058
  H     -1.948651    -0.213080     2.074485
  H      0.376277     0.583094     2.061212
  N     -3.026486    -1.837585     0.308864
  H     -3.223048    -2.673267    -0.215777
  H     -3.512819    -1.796134     1.189073
  C      2.311038    -0.034174     0.348295
  O      2.734797     0.748629     1.182204
  O      3.076247    -0.504069    -0.630264
  H      3.985585    -0.112393    -0.558139
 ---------------------------------------------------
```

Cartesian coordinates of :   ABW4_15

```
 ---------------------------------------------------
 Atomic number (AN) and Cartesian coordinates
  AN          X             Y             Z
 ---------------------------------------------------
```

O	2.241777	1.790101	1.210588
H	2.412071	0.868729	1.483484
H	1.288245	1.840989	1.102101
O	2.886295	-0.923037	1.628658
H	3.772396	-0.808216	1.242580
H	2.332933	-1.150578	0.862806
O	5.291638	-0.386574	0.184167
H	4.754152	0.203073	-0.376400
H	5.845999	0.200835	0.703755
O	3.450142	1.175332	-1.264695
H	3.811361	1.960490	-1.683756
H	2.985100	1.497475	-0.469724
C	-2.547451	-0.016866	-0.095197
C	-2.200854	-1.362533	0.017356
C	-0.923808	-1.783861	-0.263879
C	0.050519	-0.868281	-0.670670
C	-0.298702	0.480542	-0.793284
C	-1.576322	0.893841	-0.506046
H	-2.939522	-2.082803	0.328279
H	-0.660936	-2.826731	-0.173015
H	0.444578	1.190360	-1.122424
H	-1.838543	1.935156	-0.605265
N	1.339394	-1.279301	-0.897730
H	1.461640	-2.249509	-1.133550
H	1.922574	-0.648686	-1.423920
C	-3.894419	0.466250	0.194358
O	-4.248053	1.627109	0.119016
O	-4.748362	-0.500143	0.562724
H	-5.609087	-0.092331	0.729671

Cartesian coordinates of : ABW4_16

Atomic number (AN) and Cartesian coordinates

AN	X	Y	Z
O	-2.219593	-3.380822	-0.270205
H	-1.772085	-2.716085	-0.817883
H	-2.985889	-3.649296	-0.783291
O	-3.145331	0.748529	-0.916857
H	-2.416078	0.201308	-1.249367
H	-2.706923	1.527559	-0.542114
O	-1.009949	-1.088126	-1.421136
H	-0.562218	-0.829229	-0.607448
H	-0.348338	-0.973905	-2.109793
O	-3.145303	-1.126698	1.317999
H	-2.867039	-1.928837	0.849811
H	-3.202312	-0.453918	0.622030
C	1.824727	0.235573	0.152080
C	0.942873	0.192179	1.231751
C	-0.111669	1.071262	1.311202
C	-0.314401	2.025518	0.309833
C	0.568980	2.068599	-0.772642

C	1.619371	1.187189	-0.845063
H	1.085592	-0.535359	2.013578
H	-0.791270	1.032748	2.148444
H	0.415778	2.801890	-1.549511
H	2.297679	1.226674	-1.682427
N	-1.402510	2.859564	0.355770
H	-1.811823	3.003686	1.263366
H	-1.346027	3.694718	-0.201885
C	2.950148	-0.687348	0.027066
O	3.752960	-0.681604	-0.885537
O	3.035612	-1.575581	1.027106
H	3.801194	-2.142317	0.859770

Cartesian coordinates of : ABW4_17

Atomic number (AN) and Cartesian coordinates

AN	X	Y	Z
O	3.262868	-1.195206	-1.814388
H	2.919728	-1.771818	-1.105389
H	4.088644	-1.601413	-2.089743
O	3.903889	1.216734	-0.576376
H	4.435883	0.992171	0.191238
H	3.683686	0.360544	-0.993998
O	2.275441	-2.727184	0.322325
H	2.085332	-1.966612	0.897483
H	1.410632	-3.058055	0.064600
O	1.927243	3.096598	0.171430
H	1.058736	2.690198	0.125150
H	2.545211	2.394224	-0.095804
C	-2.210241	-0.011883	0.008638
C	-1.095700	0.141420	-0.813691
C	0.173027	0.117308	-0.285318
C	0.363691	-0.054316	1.088725
C	-0.753174	-0.208886	1.913383
C	-2.017257	-0.187653	1.377125
H	-1.224533	0.279637	-1.874501
H	1.033748	0.233453	-0.926224
H	-0.611171	-0.341765	2.975067
H	-2.875971	-0.303331	2.019092
N	1.635940	-0.136830	1.605000
H	2.347299	0.343727	1.075844
H	1.712827	0.006864	2.597956
C	-3.573760	0.008490	-0.515062
O	-4.577175	-0.129576	0.156984
O	-3.642899	0.198049	-1.840572
H	-4.576090	0.197790	-2.093794

Cartesian coordinates of : ABW6_ 1

```
 ---------------------------------------------------
 Atomic number (AN) and Cartesian coordinates
  AN         X            Y            Z
 ---------------------------------------------------
  O     -0.514224     2.790402    -0.845552
  H     -0.702326     3.728886    -0.766868
  H     -1.384658     2.362319    -0.960031
  O     -3.661502     0.481611     1.336574
  H     -4.301644     0.774257     1.989077
  H     -2.805203     0.393087     1.802467
  O     -1.149392     0.017223     2.401535
  H     -1.007423    -0.754505     1.842221
  H     -0.501362     0.676805     2.084036
  O     -2.922218     1.411357    -1.237496
  H     -2.433303     0.622174    -1.514377
  H     -3.251175     1.191145    -0.350510
  O     -3.750642    -1.787228    -0.291300
  H     -3.850237    -1.024577     0.309615
  H     -3.915667    -1.429694    -1.168914
  O      0.675665     1.950939     1.515810
  H      1.467213     1.465939     1.263888
  H      0.277357     2.238745     0.669896
  C      0.928788    -0.855354    -0.575897
  C      1.550331    -1.386143     0.555476
  C      2.851412    -1.071317     0.858258
  C      3.584779    -0.204632     0.035011
  C      2.961469     0.323462    -1.105122
  C      1.661686    -0.000422    -1.398880
  H      1.002225    -2.048088     1.206411
  H      3.323211    -1.486811     1.735628
  H      3.517750     0.989183    -1.747259
  H      1.188983     0.417184    -2.273675
  N      4.855157     0.146968     0.355594
  H      5.342854    -0.405014     1.037019
  H      5.422561     0.574897    -0.352782
  C     -0.469074    -1.118706    -0.888684
  O     -1.046246    -0.664868    -1.868814
  O     -1.096479    -1.889105    -0.006355
  H     -2.071284    -1.918690    -0.211199
 ---------------------------------------------------

Cartesian coordinates of :    ABW6_ 2

 ---------------------------------------------------
 Atomic number (AN) and Cartesian coordinates
  AN         X            Y            Z
 ---------------------------------------------------
  O      3.562346     2.206520     0.004344
  H      3.808937     2.675876     0.805528
  H      3.918999     1.311057     0.120245
  O     -3.217734     1.692115    -0.088667
  H     -3.693210     2.525297    -0.035888
  H     -2.589647     1.694889     0.663248
```

O	-1.286996	1.471492	1.893632
H	-1.327131	0.512201	1.955217
H	-0.495714	1.660941	1.354059
O	-1.082114	1.629808	-1.988633
H	-1.048841	0.700543	-2.235040
H	-1.854139	1.687555	-1.398856
O	-4.536661	-0.723428	0.461267
H	-4.824664	-1.117191	-0.367019
H	-4.215547	0.166566	0.220628
O	0.713149	2.275798	0.137667
H	1.669202	2.137059	0.033804
H	0.285561	1.986759	-0.684878
C	0.121361	-1.291158	-0.124009
C	0.744338	-1.273021	1.122046
C	2.105671	-1.114493	1.225735
C	2.887801	-0.966302	0.078496
C	2.268473	-1.004972	-1.173443
C	0.908221	-1.164706	-1.266592
H	0.153842	-1.374557	2.018129
H	2.580046	-1.091974	2.194943
H	2.869698	-0.897805	-2.063348
H	0.435951	-1.183679	-2.236334
N	4.233304	-0.702738	0.178360
H	4.671935	-0.969986	1.043445
H	4.784754	-0.918301	-0.635230
C	-1.332932	-1.376156	-0.266700
O	-1.911661	-1.353032	-1.340764
O	-1.992373	-1.462143	0.884851
H	-2.964391	-1.338261	0.716061

Cartesian coordinates of : ABW6_ 3

Atomic number (AN) and Cartesian coordinates

AN	X	Y	Z
O	-2.242061	-1.602807	-1.153952
H	-2.775524	-1.675380	-0.342473
H	-2.428318	-0.714562	-1.508351
O	-6.004297	-0.163255	0.567856
H	-6.357489	-0.494245	-0.261740
H	-5.476004	0.620082	0.326376
O	-2.210096	0.725904	1.524488
H	-1.482798	0.667887	0.886668
H	-2.636618	-0.146832	1.510295
O	-2.328131	1.089683	-2.012414
H	-1.510347	1.104295	-1.493777
H	-2.996426	1.473893	-1.422168
O	-4.193304	1.898229	0.002361
H	-3.499755	1.602467	0.628346
H	-4.373567	2.816406	0.218739
O	-3.745266	-1.633576	1.213739
H	-3.908714	-2.375671	1.799961

H	-4.612997	-1.218978	1.034949
C	2.207513	-0.103669	-0.129871
C	3.046201	-1.216992	-0.194899
C	4.397885	-1.093818	0.007680
C	4.965598	0.157000	0.286251
C	4.122967	1.275759	0.350494
C	2.773614	1.141138	0.145479
H	2.629839	-2.187431	-0.410657
H	5.039176	-1.960377	-0.047241
H	4.550663	2.244221	0.561266
H	2.134980	2.008838	0.194172
N	6.296581	0.275581	0.527271
H	6.895346	-0.479921	0.248352
H	6.703009	1.192504	0.491637
C	0.769979	-0.203240	-0.347455
O	0.019192	0.770288	-0.313515
O	0.327454	-1.423501	-0.585454
H	-0.661006	-1.431047	-0.756187

Cartesian coordinates of : ABW6_ 4

Atomic number (AN) and Cartesian coordinates

AN	X	Y	Z
O	0.579939	-0.896523	2.287510
H	0.651192	-1.394863	3.105496
H	0.697276	-1.553686	1.577396
O	3.177357	0.131030	1.753666
H	3.500510	-0.567144	1.159375
H	2.266267	-0.137387	1.968816
O	3.415097	0.279039	-2.019381
H	2.473611	0.283298	-1.774819
H	3.762290	1.041212	-1.542997
O	1.095003	-2.502639	-0.001871
H	0.806884	-1.743455	-0.529635
H	2.062977	-2.435499	-0.033362
O	3.859062	-1.795405	-0.202887
H	4.688914	-2.266340	-0.308098
H	3.826142	-1.130281	-0.918023
O	2.978984	2.297831	0.111580
H	3.184800	3.103367	0.593281
H	3.113539	1.560646	0.749482
C	-1.446718	0.507492	-0.361303
C	-2.317093	1.318089	0.368325
C	-3.637711	0.977427	0.519214
C	-4.141138	-0.196976	-0.057107
C	-3.267207	-1.009774	-0.792802
C	-1.949085	-0.659835	-0.936937
H	-1.949408	2.224121	0.821593
H	-4.303382	1.611042	1.085446
H	-3.645893	-1.913659	-1.245140
H	-1.288743	-1.292421	-1.509167

N	-5.436274	-0.559506	0.125551
H	-6.081289	0.135860	0.453116
H	-5.819008	-1.266567	-0.474832
C	-0.037553	0.838637	-0.522778
O	0.759140	0.115914	-1.116766
O	0.331438	1.982996	0.023782
H	1.318096	2.110000	-0.041526

Cartesian coordinates of : ABW6_ 5

Atomic number (AN) and Cartesian coordinates

AN	X	Y	Z
O	-1.802756	2.550219	-0.863761
H	-1.953867	2.335280	0.074909
H	-2.140067	1.774079	-1.327256
O	0.931871	2.713636	-0.229363
H	0.074181	2.587699	-0.669522
H	1.619728	2.329974	-0.798085
O	3.508630	0.360253	1.144482
H	2.658594	0.611908	1.533975
H	3.370444	-0.552711	0.844595
O	-2.099560	1.727202	1.836990
H	-2.274383	2.475837	2.412897
H	-1.168512	1.498668	1.995521
O	0.695794	1.010497	1.898845
H	0.780901	1.575110	1.098441
H	0.493016	0.129320	1.569687
O	3.282592	1.616750	-1.380936
H	3.872633	2.374648	-1.393006
H	3.429896	1.194860	-0.513249
C	-1.273343	-1.153720	-0.386203
C	-0.842714	-2.024753	0.614168
C	0.477848	-2.398892	0.698509
C	1.410657	-1.914292	-0.223436
C	0.981315	-1.031979	-1.219697
C	-0.339023	-0.662429	-1.296623
H	-1.549509	-2.414473	1.327876
H	0.804439	-3.076266	1.472639
H	1.697192	-0.651948	-1.931460
H	-0.662751	0.010751	-2.074394
N	2.738148	-2.245278	-0.114489
H	2.945321	-3.075709	0.414043
H	3.285280	-2.152937	-0.954004
C	-2.672409	-0.759861	-0.519373
O	-3.110138	-0.036952	-1.396597
O	-3.472829	-1.269880	0.422447
H	-4.372397	-0.955381	0.256903

Cartesian coordinates of : ABW6_ 6

```
 ---------------------------------------------------
 Atomic number (AN) and Cartesian coordinates
  AN          X             Y             Z
 ---------------------------------------------------
  O     -5.412391     -1.222501      0.045664
  H     -5.219715     -1.339069     -0.888682
  H     -4.653736     -1.628467      0.508717
  O     -4.682583      1.394495      0.573436
  H     -5.050933      0.507121      0.386718
  H     -5.355962      1.877251      1.057776
  O     -2.244946      0.452174      1.697342
  H     -3.043105      0.876389      1.341480
  H     -1.539411      0.636967      1.057402
  O     -2.338422     -0.650128     -1.584401
  H     -2.539017      0.308236     -1.559985
  H     -2.819567     -1.024414     -0.839891
  O     -3.169837     -2.153230      1.441752
  H     -2.752043     -1.272867      1.536665
  H     -2.577633     -2.654084      0.874850
  O     -2.894931      2.070612     -1.432593
  H     -3.342173      2.347630     -2.236244
  H     -3.599582      1.973137     -0.766843
  C      2.213929      0.035464     -0.172886
  C      2.994640     -0.957260     -0.765322
  C      4.353690     -0.993716     -0.574225
  C      4.987019     -0.031899      0.223929
  C      4.202791      0.966704      0.817357
  C      2.845690      0.994071      0.618824
  H      2.527881     -1.705202     -1.385274
  H      4.949244     -1.764727     -1.039304
  H      4.680823      1.714659      1.431589
  H      2.252986      1.768171      1.079907
  N      6.325649     -0.090002      0.450671
  H      6.882280     -0.654421     -0.165029
  H      6.776383      0.728476      0.817210
  C      0.767975      0.101277     -0.359190
  O      0.065044      0.981505      0.123953
  O      0.261903     -0.873330     -1.099432
  H     -0.721348     -0.749392     -1.222454
 ---------------------------------------------------

Cartesian coordinates of :    ABW6_ 7

 ---------------------------------------------------
 Atomic number (AN) and Cartesian coordinates
  AN          X             Y             Z
 ---------------------------------------------------
  O      3.088078      3.210692     -0.448534
  H      3.507073      3.853826      0.128700
  H      2.376304      2.811836      0.088459
  O      4.446123      0.737907     -0.140301
  H      4.029139      0.455104      0.690863
  H      4.073099      1.622084     -0.305027
  O      2.478574     -2.657684      0.762979
```

H	1.544089	-2.617005	0.503193
H	2.922701	-2.345366	-0.038119
O	1.141082	1.802370	0.993989
H	0.742443	1.325449	0.256795
H	1.692182	1.130322	1.431827
O	2.855811	-0.200221	2.027574
H	3.109742	-0.306351	2.947330
H	2.692686	-1.101019	1.682988
O	2.970732	-1.086400	-1.570570
H	3.209014	-1.136061	-2.499564
H	3.543463	-0.396347	-1.170651
C	-1.731625	-0.521925	-0.346497
C	-2.029512	0.807172	-0.650264
C	-3.278321	1.323103	-0.409591
C	-4.284581	0.522295	0.147198
C	-3.986490	-0.813880	0.449087
C	-2.734644	-1.318471	0.205984
H	-1.271062	1.440748	-1.081157
H	-3.498447	2.352563	-0.648452
H	-4.755571	-1.440407	0.874671
H	-2.517444	-2.348271	0.441422
N	-5.508852	1.039687	0.425242
H	-5.763175	1.907917	-0.009123
H	-6.262855	0.400353	0.598353
C	-0.413118	-1.097334	-0.583927
O	-0.120928	-2.256407	-0.320432
O	0.463724	-0.252746	-1.114341
H	1.352751	-0.673029	-1.264067

Cartesian coordinates of : ABW6_ 8

Atomic number (AN) and Cartesian coordinates

AN	X	Y	Z
O	-3.900352	-1.372968	-1.232717
H	-4.202158	-1.373014	-0.307170
H	-3.675189	-0.446374	-1.410180
O	-1.217511	-2.064953	-0.663265
H	-2.144854	-1.911660	-0.927786
H	-0.738587	-1.323428	-1.052062
O	-1.749360	-0.877638	1.800332
H	-1.442580	-1.333835	0.991553
H	-1.155105	-1.141067	2.506659
O	-4.519070	-1.325655	1.548106
H	-3.581084	-1.191373	1.769613
H	-4.934020	-0.475270	1.714528
O	-2.927121	1.320418	-1.448750
H	-2.975873	1.543753	-0.496696
H	-3.285826	2.076018	-1.921458
O	-2.554576	1.779670	1.256833
H	-2.321603	0.872155	1.518448
H	-1.706437	2.147119	0.967519

```
  C      1.716413      0.624366     -0.300782
  C      2.128418     -0.505614     -1.008970
  C      3.347700     -1.085666     -0.763366
  C      4.207329     -0.553912      0.207357
  C      3.795377      0.583779      0.915661
  C      2.574866      1.155310      0.662291
  H      1.485144     -0.931221     -1.762097
  H      3.657206     -1.959083     -1.316964
  H      4.452375      1.003795      1.662119
  H      2.268355      2.030511      1.213038
  N      5.395011     -1.150263      0.483383
  H      5.759459     -1.808476     -0.180643
  H      6.076722     -0.628885      1.003573
  C      0.422814      1.256720     -0.526245
  O      0.029941      2.241674      0.083535
  O     -0.316276      0.672392     -1.465100
  H     -1.223913      1.068755     -1.503140
 ---------------------------------------------------

Cartesian coordinates of :    ABW6_ 9

 ---------------------------------------------------
 Atomic number (AN) and Cartesian coordinates
  AN          X            Y            Z
 ---------------------------------------------------
  O     -3.610426      1.941438     -0.712794
  H     -3.198805      1.461816     -1.463459
  H     -3.495252      2.878467     -0.885994
  O     -5.794027      0.582717      0.386832
  H     -5.096170      1.138717     -0.002993
  H     -5.949547      0.952230      1.259715
  O     -2.395684      0.714023      1.472675
  H     -2.820448      1.193924      0.735255
  H     -2.696178     -0.195182      1.375517
  O     -2.418196      0.375255     -2.609492
  H     -3.041926      0.133104     -3.298344
  H     -2.332635     -0.422573     -2.045309
  O     -2.161397     -1.781071     -0.917365
  H     -2.946321     -1.798357     -0.339476
  H     -1.415445     -1.560851     -0.337729
  O     -4.474248     -1.859129      0.671510
  H     -4.945825     -1.004569      0.619305
  H     -4.245915     -1.965233      1.598531
  C      2.231915     -0.075681      0.358541
  C      2.895657      1.146798      0.464440
  C      4.239021      1.245605      0.199144
  C      4.973385      0.116712     -0.187650
  C      4.307130     -1.112177     -0.290669
  C      2.964886     -1.199568     -0.021400
  H      2.349111      2.027135      0.761056
  H      4.743997      2.195711      0.286518
  H      4.864457     -1.988854     -0.584150
  H      2.463452     -2.150803     -0.103859
  N      6.292838      0.218318     -0.496281
```

H	6.786555	1.035786	-0.187562
H	6.832530	-0.627050	-0.534804
C	0.804746	-0.209773	0.631983
O	0.203156	-1.276429	0.572131
O	0.196618	0.920139	0.955987
H	-0.772305	0.761323	1.139554

Cartesian coordinates of : ABW6_10

Atomic number (AN) and Cartesian coordinates

AN	X	Y	Z
O	3.362092	-1.462199	1.332500
H	4.324591	-1.413399	1.153414
H	3.233599	-2.199542	1.933446
O	5.994260	1.210981	-0.717661
H	5.047072	1.382592	-0.876260
H	6.278893	1.910667	-0.124520
O	2.151867	1.101321	1.419922
H	1.280944	0.949064	1.018531
H	2.545559	0.216981	1.505614
O	3.246482	1.504030	-1.108920
H	2.892773	2.279303	-1.552016
H	2.881451	1.521999	-0.201389
O	6.025864	-1.176078	0.709468
H	6.037639	-0.340231	0.201169
H	6.252225	-1.858648	0.072822
O	2.088004	-1.124462	-1.139940
H	2.413329	-0.228030	-1.322338
H	2.506110	-1.354470	-0.289534
C	-2.491240	-0.011168	-0.137349
C	-3.269102	-1.088038	-0.562903
C	-4.635614	-1.067435	-0.430714
C	-5.279799	0.040201	0.135953
C	-4.497968	1.120954	0.566088
C	-3.133300	1.089622	0.428911
H	-2.793685	-1.950563	-1.000663
H	-5.228058	-1.906991	-0.761645
H	-4.983609	1.977682	1.008222
H	-2.542316	1.926490	0.765908
N	-6.635217	0.083632	0.229202
H	-7.139124	-0.780053	0.142378
H	-7.040800	0.763228	0.846430
C	-1.036602	-0.006047	-0.262963
O	-0.335321	0.937135	0.086603
O	-0.524438	-1.105104	-0.790462
H	0.471208	-1.035036	-0.878221

Cartesian coordinates of : ABW6_11

Atomic number (AN) and Cartesian coordinates

AN	X	Y	Z
O	4.777349	2.579525	-0.184244
H	4.874485	3.079007	0.630510
H	3.884795	2.199811	-0.139822
O	5.691479	-0.107122	0.203358
H	5.065985	-0.383876	0.890821
H	5.514475	0.843169	0.096671
O	2.174489	-2.438800	0.022949
H	1.333108	-1.957451	-0.028043
H	2.718481	-2.042998	-0.676372
O	2.369878	0.989241	-0.089814
H	2.713404	0.429254	-0.808149
H	2.659482	0.524332	0.715294
O	3.443326	-0.778743	1.858216
H	3.211804	-0.909689	2.780551
H	2.986636	-1.482251	1.355793
O	3.786675	-0.867440	-1.709764
H	4.066431	-0.846073	-2.627659
H	4.572387	-0.646590	-1.173540
C	-2.392304	0.025391	-0.042972
C	-3.034173	1.261545	0.033191
C	-4.404073	1.344089	0.071265
C	-5.188940	0.184167	0.032764
C	-4.544049	-1.057692	-0.048568
C	-3.174399	-1.128420	-0.083945
H	-2.449027	2.166174	0.061983
H	-4.891113	2.305676	0.129915
H	-5.139481	-1.957394	-0.083671
H	-2.689887	-2.089835	-0.147771
N	-6.543468	0.259405	0.113867
H	-6.975961	1.146692	-0.067627
H	-7.075885	-0.547360	-0.156338
C	-0.937563	-0.092468	-0.082404
O	-0.351345	-1.166322	-0.134006
O	-0.293777	1.064769	-0.059116
H	0.692567	0.933033	-0.086241

Cartesian coordinates of : ABW6_12

Atomic number (AN) and Cartesian coordinates

AN	X	Y	Z
O	1.753911	-2.700220	-0.541535
H	2.134126	-2.033421	-1.124351
H	1.774313	-2.280676	0.338614
O	-1.053572	-2.687121	-0.253360
H	-1.082479	-2.145612	0.552571
H	-0.115941	-2.685683	-0.512513
O	-3.583876	-0.056006	0.832811
H	-3.342144	0.848000	0.573912

H	-3.535186	-0.567864	0.003860
O	1.552567	-1.469113	2.004458
H	2.064497	-0.655603	2.014461
H	0.627980	-1.179525	1.949136
O	-1.251821	-0.937882	2.021756
H	-1.544339	-1.458702	2.774318
H	-2.071415	-0.570509	1.627004
O	-3.235718	-1.567437	-1.529987
H	-3.890059	-2.269417	-1.569473
H	-2.426585	-1.993670	-1.184361
C	1.431146	1.011419	-0.397869
C	1.060088	1.895614	0.614262
C	-0.219863	2.390625	0.677325
C	-1.170703	2.012360	-0.273709
C	-0.808910	1.100480	-1.269401
C	0.473975	0.613667	-1.328760
H	1.785390	2.207050	1.347798
H	-0.498797	3.084669	1.455312
H	-1.544346	0.793569	-1.996757
H	0.750823	-0.079513	-2.107261
N	-2.459934	2.482718	-0.198227
H	-2.589736	3.319292	0.345320
H	-2.974870	2.484644	-1.063151
C	2.793478	0.503124	-0.518986
O	3.188777	-0.222346	-1.413841
O	3.611860	0.904621	0.460729
H	4.486030	0.524232	0.297724

Cartesian coordinates of : ABW6_13

Atomic number (AN) and Cartesian coordinates

AN	X	Y	Z
O	-1.923783	1.873763	0.855788
H	-2.061815	0.992240	1.248196
H	-1.235105	1.738121	0.187242
O	-4.458012	2.132325	-0.255712
H	-3.540727	2.127216	0.085686
H	-4.367808	2.070364	-1.210457
O	-4.069572	-2.522413	-0.652841
H	-3.524679	-3.005644	-0.025766
H	-4.543349	-1.865570	-0.113697
O	-2.529364	-0.685936	1.896277
H	-3.466103	-0.621015	1.637433
H	-2.527931	-0.642010	2.855734
O	-2.394509	-0.473091	-1.570518
H	-2.749366	0.282233	-1.091217
H	-2.951222	-1.227172	-1.293049
O	-5.105731	-0.356126	0.796138
H	-5.877321	-0.280052	1.362141
H	-4.967514	0.526856	0.396993
C	2.192772	0.090267	-0.326690

C	2.742111	-1.167212	-0.075716
C	4.072661	-1.306815	0.232088
C	4.909137	-0.184818	0.301510
C	4.357350	1.078438	0.047397
C	3.026543	1.206186	-0.259764
H	2.116397	-2.043270	-0.127419
H	4.489223	-2.284581	0.421031
H	4.994060	1.948847	0.094784
H	2.612940	2.182892	-0.455300
N	6.217166	-0.313400	0.647177
H	6.631634	-1.225469	0.585942
H	6.830720	0.457174	0.454370
C	0.784047	0.268086	-0.660643
O	0.281380	1.356758	-0.918937
O	0.071098	-0.847598	-0.665259
H	-0.861969	-0.663603	-0.971464

Cartesian coordinates of : ABW6_14

Atomic number (AN) and Cartesian coordinates

AN	X	Y	Z
O	1.778086	2.983643	-0.845097
H	1.019938	2.843257	-1.419123
H	2.433992	2.321282	-1.135828
O	-0.022660	-0.637953	2.429285
H	0.329005	0.211607	2.116229
H	-0.818747	-0.780281	1.909099
O	1.200906	1.830546	1.642639
H	1.441795	2.478719	2.309092
H	1.317536	2.276615	0.780387
O	3.309245	-0.000334	1.078976
H	2.927481	-0.884890	1.211285
H	2.607594	0.613632	1.355586
O	3.610513	0.938898	-1.507557
H	3.553715	0.532606	-0.618850
H	4.506854	1.277621	-1.572935
O	1.919870	-2.461308	1.471361
H	1.747120	-2.537888	0.520270
H	1.192930	-1.903803	1.804605
C	-2.160837	-0.002642	-0.403678
C	-2.218190	-1.394889	-0.464365
C	-1.112342	-2.127394	-0.824723
C	0.088731	-1.485581	-1.138413
C	0.147639	-0.091121	-1.075103
C	-0.961146	0.633817	-0.714637
H	-3.136995	-1.905366	-0.226992
H	-1.162584	-3.204598	-0.868594
H	1.073523	0.407862	-1.310546
H	-0.907766	1.710070	-0.664491
N	1.213176	-2.215749	-1.433269
H	1.061613	-3.142025	-1.795000

```
  H      1.958171     -1.715437     -1.888718
  C     -3.310620      0.809202     -0.014354
  O     -3.313406      2.023171      0.046479
  O     -4.403634      0.092609      0.285243
  H     -5.108181      0.708737      0.528423
 ---------------------------------------------------

Cartesian coordinates of :    ABW6_15

 ---------------------------------------------------
 Atomic number (AN) and Cartesian coordinates
  AN          X             Y             Z
 ---------------------------------------------------
  O     -2.041023     -2.461354     -0.723606
  H     -2.584133     -1.789220     -1.162988
  H     -1.219621     -1.998333     -0.496954
  O     -3.510220     -0.258993     -1.827366
  H     -4.421890      0.061201     -1.666649
  H     -3.196316      0.209181     -2.604840
  O     -4.899134      2.029612      1.085491
  H     -4.016390      1.628804      0.997750
  H     -4.776077      2.954840      0.858391
  O     -2.456610      0.647898      0.612951
  H     -2.546868     -0.144733      1.176220
  H     -2.767441      0.359584     -0.267641
  O     -2.696815     -1.823219      1.967977
  H     -1.858063     -1.939377      2.422964
  H     -2.548570     -2.204818      1.083856
  O     -5.994195      0.742986     -1.149858
  H     -5.690026      1.209212     -0.347099
  H     -6.542964      0.022466     -0.830039
  C      2.307579      0.056074      0.080807
  C      2.889934      1.322267      0.021721
  C      4.248040      1.468409     -0.116286
  C      5.080496      0.344352     -0.199701
  C      4.495484     -0.927871     -0.136414
  C      3.137103     -1.062098     -0.000074
  H      2.267792      2.199995      0.086041
  H      4.688943      2.452752     -0.160255
  H      5.128086     -1.800584     -0.194775
  H      2.698190     -2.046105      0.049094
  N      6.420208      0.483023     -0.378747
  H      6.831129      1.378179     -0.186384
  H      7.003537     -0.312887     -0.195658
  C      0.867701     -0.129645      0.227592
  O      0.331327     -1.229851      0.281196
  O      0.172868      0.995980      0.303807
  H     -0.798372      0.805284      0.419582
 ---------------------------------------------------

Cartesian coordinates of :    ABW6_16

 ---------------------------------------------------
 Atomic number (AN) and Cartesian coordinates
```

AN	X	Y	Z
O	3.738536	1.892798	-2.091545
H	3.110323	2.325843	-2.674944
H	3.323874	1.043715	-1.856576
O	2.532803	-0.430729	-1.044661
H	2.710536	-0.125612	-0.137386
H	2.974495	-1.301119	-1.098743
O	4.248838	-1.761962	1.776609
H	3.832866	-2.315336	2.442264
H	3.714078	-0.949506	1.752705
O	3.498752	3.003887	0.477730
H	4.384648	3.192528	0.797101
H	3.619826	2.682508	-0.436224
O	3.885443	-2.849528	-0.772188
H	4.065986	-2.549042	0.139963
H	4.729974	-2.791641	-1.226082
O	2.682264	0.590593	1.621135
H	1.721441	0.528250	1.495236
H	2.946959	1.470077	1.288276
C	-2.171522	-0.077030	0.022084
C	-2.885797	-0.452359	-1.115857
C	-4.258295	-0.419388	-1.130291
C	-4.972681	-0.005815	0.001804
C	-4.255064	0.367335	1.146448
C	-2.883714	0.330441	1.149664
H	-2.355549	-0.774570	-1.997105
H	-4.801769	-0.713035	-2.015635
H	-4.795563	0.683086	2.025981
H	-2.343130	0.619324	2.037041
N	-6.329190	0.071298	-0.022209
H	-6.815180	-0.418120	-0.751342
H	-6.811618	0.135762	0.855547
C	-0.712348	-0.098429	0.064587
O	-0.064069	0.224871	1.052480
O	-0.138281	-0.497818	-1.060642
H	0.854361	-0.487628	-0.986596

Cartesian coordinates of :   ABW6_17

Atomic number (AN) and Cartesian coordinates

AN	X	Y	Z
O	-2.336620	0.227074	-2.043353
H	-2.763448	-0.525471	-1.606734
H	-2.288851	0.911881	-1.358981
O	-3.446007	-1.971041	-0.583725
H	-3.701917	-1.532185	0.250633
H	-4.161359	-2.569919	-0.809060
O	-1.030445	-0.159925	1.460893
H	-0.222085	0.090569	1.915680
H	-0.793790	-0.907823	0.876999

O	-2.194045	2.086943	0.200768
H	-1.484673	2.734368	0.090551
H	-1.766179	1.342299	0.659170
O	-0.711973	-2.408903	-0.151975
H	-0.224357	-2.187124	-0.950135
H	-1.652455	-2.375617	-0.401897
O	-3.802191	-0.562725	1.798070
H	-2.848373	-0.386319	1.852540
H	-4.192170	0.285327	1.569121
C	2.128328	-0.250565	-0.106338
C	2.338708	0.637367	0.947326
C	1.826225	1.912797	0.902919
C	1.088361	2.340379	-0.204553
C	0.894515	1.455468	-1.270105
C	1.403229	0.183196	-1.214944
H	2.903104	0.323691	1.810128
H	1.986608	2.593898	1.724635
H	0.324340	1.780029	-2.126974
H	1.236030	-0.498880	-2.034000
N	0.499405	3.577788	-0.223224
H	0.857578	4.254132	0.428722
H	0.289480	3.956774	-1.131004
C	2.616722	-1.625422	-0.080920
O	2.457373	-2.435034	-0.974221
O	3.273939	-1.948955	1.042606
H	3.549912	-2.872938	0.970360

Cartesian coordinates of : ABW6_18

Atomic number (AN) and Cartesian coordinates

AN	X	Y	Z
O	3.799107	-0.551702	1.825289
H	4.182256	0.303695	1.612970
H	2.840213	-0.391065	1.851928
O	3.496299	-1.947610	-0.568788
H	3.735397	-1.509746	0.271192
H	4.215031	-2.547703	-0.779581
O	0.747775	-2.395784	-0.231413
H	0.312572	-2.116065	-1.041575
H	1.700948	-2.363400	-0.428708
O	2.188000	2.084684	0.186830
H	1.757094	1.346561	0.652157
H	1.477106	2.726378	0.051766
O	1.042944	-0.175645	1.439224
H	0.807438	-0.916808	0.847085
H	0.263901	-0.000483	1.973181
O	2.334007	0.221990	-2.046079
H	2.780270	-0.519221	-1.610172
H	2.283861	0.910043	-1.364647
C	-2.163232	-0.241027	-0.029841
C	-1.468192	0.149635	-1.173545

C	-0.938764	1.412251	-1.275225
C	-1.085935	2.328275	-0.229885
C	-1.793460	1.942670	0.912808
C	-2.321257	0.677780	1.005788
H	-1.336305	-0.546326	-1.986124
H	-0.390773	1.704330	-2.157912
H	-1.916213	2.647896	1.720580
H	-2.863138	0.384960	1.891064
N	-0.481231	3.556792	-0.301186
H	-0.293108	3.905250	-1.225907
H	-0.817083	4.257219	0.337237
C	-2.709102	-1.585653	0.125184
O	-3.288050	-1.990552	1.115024
O	-2.518505	-2.374930	-0.941475
H	-2.903812	-3.240232	-0.746820

Cartesian coordinates of :   ABW6_19

Atomic number (AN) and Cartesian coordinates

AN	X	Y	Z
O	-0.708626	3.123253	-1.449817
H	-0.905528	4.051619	-1.596560
H	-1.506753	2.755834	-1.017070
O	-1.019972	1.084578	2.018683
H	-0.299545	1.638152	1.654852
H	-1.223499	1.459782	2.879157
O	-2.771946	-1.171707	1.516953
H	-2.315130	-1.881545	1.042384
H	-2.108619	-0.473733	1.629728
O	0.847485	2.809951	0.864314
H	1.643171	2.349807	0.574844
H	0.350410	2.971684	0.039181
O	-2.807759	2.068808	-0.015453
H	-2.289124	1.721177	0.728773
H	-3.246641	1.290573	-0.398745
O	-4.025297	-0.357821	-0.869553
H	-3.691762	-0.689614	-0.014964
H	-4.977052	-0.290315	-0.759302
C	2.033591	-0.695680	-0.231284
C	0.908341	-0.161939	-0.858293
C	-0.240793	-0.904010	-0.988160
C	-0.297206	-2.210449	-0.492867
C	0.835089	-2.750624	0.125021
C	1.978103	-2.002296	0.250708
H	0.935737	0.840889	-1.250445
H	-1.110189	-0.483380	-1.470796
H	0.797093	-3.760238	0.504595
H	2.846982	-2.423510	0.730853
N	-1.464519	-2.926871	-0.548677
H	-2.136457	-2.635657	-1.238598
H	-1.384718	-3.923632	-0.443842

```
  C      3.266732      0.063811     -0.058034
  O      4.293935     -0.373690      0.420953
  O      3.183414      1.338547     -0.474920
  H      4.040239      1.761981     -0.325134
 ---------------------------------------------------

Cartesian coordinates of :    ABW6_20

 ---------------------------------------------------
 Atomic number (AN) and Cartesian coordinates
  AN          X            Y            Z
 ---------------------------------------------------
  O     -0.061713      2.938893     -0.137663
  H     -0.229887      3.832743      0.171472
  H     -0.941884      2.579765     -0.362896
  O     -4.927957     -0.439442      0.518775
  H     -5.474379      0.348357      0.453730
  H     -4.176948     -0.170555      1.065355
  O     -2.595608      1.833082     -0.467559
  H     -2.555422      1.295278      0.339566
  H     -2.814535      1.186231     -1.155900
  O      0.163685      1.288948      2.136934
  H      0.778405      0.581879      1.920050
  H      0.157743      1.865938      1.349253
  O     -3.502403     -0.489293     -1.929128
  H     -4.085930     -0.535545     -1.149487
  H     -4.089051     -0.413286     -2.685458
  O     -2.313600     -0.002783      1.791968
  H     -2.054298     -0.742948      1.217793
  H     -1.472975      0.452874      1.990726
  C      2.486392     -0.473829     -0.240419
  C      1.500304      0.084428     -1.051536
  C      0.225731     -0.429862     -1.063949
  C     -0.103168     -1.524010     -0.258822
  C      0.886309     -2.086440      0.554703
  C      2.157303     -1.567821      0.559268
  H      1.733660      0.931092     -1.675640
  H     -0.531938      0.009448     -1.694179
  H      0.637708     -2.931256      1.178787
  H      2.914649     -2.006845      1.188985
  N     -1.389935     -1.994414     -0.212869
  H     -1.996664     -1.735276     -0.975608
  H     -1.511021     -2.935166      0.120652
  C      3.848576      0.049265     -0.195781
  O      4.743410     -0.403249      0.491995
  O      4.051717      1.105816     -0.996515
  H      4.973378      1.382004     -0.898364
 ---------------------------------------------------

Cartesian coordinates of :    ABW6_21

 ---------------------------------------------------
 Atomic number (AN) and Cartesian coordinates
  AN          X            Y            Z
```

```
 ---------------------------------------------------
  O      3.803551     -0.013511      0.076696
  H      3.367363      0.142237     -0.779965
  H      3.304499      0.511181      0.722382
  O      2.509147      0.180054     -2.447004
  H      2.072283     -0.687271     -2.346715
  H      3.192633      0.041506     -3.107550
  O      2.300152     -2.368550      0.635594
  H      1.724966     -2.014162      1.335893
  H      2.902850     -1.626701      0.445814
  O      2.219298      1.389000      2.052981
  H      1.824210      1.997389      1.408211
  H      2.668356      1.943537      2.696175
  O      0.871452     -1.042375      2.728653
  H      1.305808     -1.339104      3.532558
  H      1.275345     -0.179910      2.532960
  O      1.289614     -2.328318     -1.971787
  H      0.352302     -2.116770     -1.969525
  H      1.538678     -2.375473     -1.027361
  C     -2.378053      0.189050     -0.093098
  C     -1.121385     -0.414360     -0.089221
  C      0.016172      0.342462     -0.226244
  C     -0.066094      1.728187     -0.374388
  C     -1.323453      2.336107     -0.370058
  C     -2.457872      1.573174     -0.231860
  H     -1.032856     -1.482763      0.028255
  H      0.987849     -0.124513     -0.205662
  H     -1.394778      3.407102     -0.483152
  H     -3.426761      2.046533     -0.236169
  N      1.087867      2.473011     -0.457145
  H      1.869739      1.995135     -0.879064
  H      0.975145      3.416092     -0.788809
  C     -3.611235     -0.582223      0.038931
  O     -4.730577     -0.107852      0.052439
  O     -3.412169     -1.904138      0.144486
  H     -4.274920     -2.333978      0.220914
 ---------------------------------------------------
```

Cartesian coordinates of :   ABW6_22

```
 ---------------------------------------------------
 Atomic number (AN) and Cartesian coordinates
  AN          X             Y             Z
 ---------------------------------------------------
  O      4.489537      2.594857     -0.550746
  H      4.682160      3.171776      0.192329
  H      3.594248      2.238744     -0.375909
  O      6.055697      0.316054     -0.350474
  H      5.537384      1.141520     -0.434905
  H      6.431654      0.166072     -1.221271
  O      1.808653     -0.891186     -1.670398
  H      1.531314     -1.148217     -2.553116
  H      1.177038     -1.310714     -1.057159
  O      1.990775      1.491595     -0.101904
```

```
  H      1.882572      0.818610     -0.791977
  H      2.099272      0.965510      0.709306
  O      2.430521     -0.392049      1.992078
  H      2.949811     -0.115658      2.750995
  H      3.060119     -0.839561      1.390841
  O      4.019308     -1.567984      0.028789
  H      4.739338     -0.926975     -0.123950
  H      3.371725     -1.391146     -0.670709
  C     -3.477955      0.105970      0.000036
  C     -2.415882      0.709373      0.671122
  C     -1.230780      0.038434      0.855912
  C     -1.071691     -1.261765      0.369789
  C     -2.138275     -1.870259     -0.297747
  C     -3.319358     -1.193469     -0.477232
  H     -2.521695      1.711291      1.053340
  H     -0.413379      0.509719      1.378941
  H     -2.021784     -2.876570     -0.670415
  H     -4.138774     -1.669104     -0.992222
  N      0.132733     -1.911036      0.496838
  H      0.751667     -1.563982      1.214456
  H      0.098013     -2.914692      0.436115
  C     -4.751248      0.787910     -0.215275
  O     -5.707257      0.307734     -0.792835
  O     -4.792629      2.029974      0.288769
  H     -5.665529      2.401494      0.101592
 ---------------------------------------------------

Cartesian coordinates of :    ABW6_23

 ---------------------------------------------------
 Atomic number (AN) and Cartesian coordinates
  AN         X             Y             Z
 ---------------------------------------------------
  O     -2.250682      2.223032      1.225579
  H     -2.954532      2.024258      0.576999
  H     -2.580608      2.954511      1.752802
  O     -2.509163     -0.504565      2.044474
  H     -3.428990     -0.665999      1.783701
  H     -2.386749      0.450834      1.916519
  O     -4.062354     -2.264544     -1.450037
  H     -4.006446     -3.220961     -1.385113
  H     -3.171285     -1.938422     -1.211695
  O     -4.089042      1.327008     -0.652090
  H     -3.502910      0.909970     -1.290331
  H     -4.483966      0.584450     -0.160461
  O     -5.040098     -0.929668      0.770172
  H     -5.973578     -1.038258      0.965774
  H     -4.842002     -1.525904      0.021729
  O     -1.641816     -1.261088     -0.553442
  H     -1.330445     -0.430654     -0.942295
  H     -1.877947     -1.023491      0.362431
  C      3.355509     -0.052740     -0.023537
  C      2.344968      0.127369      0.920208
  C      1.122084      0.632672      0.551217
```

```
  C      0.871245      0.978583     -0.780893
  C      1.885444      0.800488     -1.727082
  C      3.103887      0.292935     -1.349915
  H      2.520361     -0.133688      1.950890
  H      0.341035      0.760868      1.285141
  H      1.698425      1.067591     -2.756134
  H      3.881595      0.159805     -2.085084
  N     -0.368353      1.425485     -1.157789
  H     -0.928573      1.851060     -0.434414
  H     -0.422714      1.886151     -2.049824
  C      4.664652     -0.591449      0.330257
  O      5.576278     -0.775166     -0.453558
  O      4.798380     -0.883008      1.632941
  H      5.689054     -1.233682      1.770031
 ---------------------------------------------------


Cartesian coordinates of :    ABW8_ 1

 ---------------------------------------------------
 Atomic number (AN) and Cartesian coordinates
  AN          X             Y             Z
 ---------------------------------------------------
  O      0.375807      2.299024      1.393682
  H      0.197123      2.251943      0.447549
  H      1.345499      2.214024      1.445616
  O      3.661811     -1.267071     -1.156186
  H      4.344809     -1.763104     -1.613745
  H      2.911513     -1.882282     -1.039359
  O      2.956773      1.468227     -1.590352
  H      3.096117      1.754929     -0.670497
  H      3.183956      0.522513     -1.576165
  O     -0.846847      0.008216      2.416569
  H     -1.595695     -0.193217      1.847775
  H     -0.449067      0.818343      2.039010
  O      1.356954     -2.772605     -0.609629
  H      0.844664     -2.125049     -1.116702
  H      1.234604     -2.509666      0.319826
  O      1.131396     -1.876100      2.058570
  H      1.038723     -2.539480      2.746596
  H      0.405083     -1.234041      2.203152
  O      3.143117      1.970330      1.203686
  H      3.368235      1.044914      1.442353
  H      3.790554      2.528006      1.641312
  O      3.662015     -0.686077      1.633903
  H      3.800011     -0.916903      0.700079
  H      2.805015     -1.090639      1.856072
  C     -1.538261      0.213754     -1.074850
  C     -2.277612      1.305045     -0.615805
  C     -3.509052      1.129923     -0.036061
  C     -4.047124     -0.155513      0.118271
  C     -3.306746     -1.251599     -0.348130
  C     -2.081886     -1.063609     -0.936494
  H     -1.879284      2.302134     -0.716283
```

H	-4.073871	1.981251	0.312082
H	-3.713060	-2.245370	-0.236708
H	-1.520152	-1.916263	-1.284387
N	-5.239084	-0.338672	0.739768
H	-5.840076	0.456119	0.857966
H	-5.696406	-1.225884	0.636910
C	-0.199335	0.360163	-1.630656
O	0.447130	-0.561138	-2.111176
O	0.304875	1.587705	-1.558868
H	1.291133	1.545360	-1.720075

Cartesian coordinates of : ABW8_ 2

Atomic number (AN) and Cartesian coordinates

AN	X	Y	Z
O	3.039982	-1.377640	1.995309
H	2.565850	-0.519741	2.046374
H	3.070286	-1.729573	2.887685
O	1.613825	1.351942	-1.709609
H	0.886217	1.019015	-1.165006
H	2.185886	1.837175	-1.088681
O	3.271947	2.576017	0.202333
H	3.342455	3.527856	0.304453
H	4.189291	2.234392	0.144183
O	2.918346	-1.026780	-2.093751
H	2.937391	-1.274126	-3.020952
H	2.471579	-0.154555	-2.044773
O	5.767223	1.440532	0.041212
H	5.510529	0.497211	-0.029123
H	6.148312	1.661109	-0.812328
O	1.654874	0.974879	1.863600
H	0.939358	0.718466	1.262808
H	2.217216	1.572214	1.339903
O	1.510314	-2.428314	-0.114574
H	1.965154	-1.998250	-0.861545
H	2.006476	-2.145789	0.674586
O	4.928384	-1.190571	-0.110350
H	4.301557	-1.165384	-0.852760
H	4.366416	-1.291833	0.675525
C	-2.754083	-0.213106	-0.002039
C	-3.720300	-1.219121	-0.039024
C	-5.057996	-0.911841	-0.038304
C	-5.482299	0.423495	-0.001218
C	-4.511249	1.433895	0.038585
C	-3.177381	1.115293	0.037857
H	-3.414621	-2.252345	-0.066568
H	-5.798406	-1.696990	-0.065667
H	-4.827407	2.465424	0.071443
H	-2.439023	1.900705	0.070794
N	-6.803525	0.733211	-0.042720
H	-7.466298	0.008035	0.161996

```
  H     -7.078883      1.664122      0.211569
  C     -1.326612     -0.507439     -0.001499
  O     -0.464624      0.368921      0.027838
  O     -1.021391     -1.789100     -0.034579
  H     -0.026166     -1.935748     -0.050451
 ---------------------------------------------------

Cartesian coordinates of :    ABW8_ 3

 ---------------------------------------------------
 Atomic number (AN) and Cartesian coordinates
  AN          X            Y            Z
 ---------------------------------------------------
  O      1.865677      1.615155      1.834647
  H      0.913171      1.418699      1.859846
  H      2.288701      0.766478      1.672128
  O     -3.687622      1.690934     -0.691103
  H     -2.779849      1.999565     -0.816600
  H     -3.717466      0.832576     -1.142630
  O     -0.832335      2.170888     -1.200203
  H     -0.713492      1.260750     -1.496381
  H      0.068843      2.513811     -1.039248
  O     -3.730331     -2.056764      0.945326
  H     -3.792084     -1.186210      1.375212
  H     -2.778082     -2.177612      0.802320
  O      1.733710      3.084418     -0.570620
  H      1.852705      2.576386      0.254664
  H      1.646851      3.998873     -0.289423
  O     -3.629549      0.607224      1.872057
  H     -3.751390      1.054384      1.010070
  H     -4.223766      1.046683      2.485244
  O     -0.895592      1.396845      1.582921
  H     -1.777082      1.084461      1.837800
  H     -0.975690      1.658547      0.651873
  O     -3.587063     -1.009971     -1.603557
  H     -3.976379     -1.526144     -2.313913
  H     -3.818684     -1.460554     -0.765789
  C      1.098360     -1.148010     -0.354377
  C      1.786080     -0.328640     -1.250599
  C      3.133631     -0.106573     -1.113185
  C      3.852082     -0.703300     -0.068345
  C      3.160304     -1.524388      0.835280
  C      1.812250     -1.737745      0.688732
  H      1.257946      0.140012     -2.065101
  H      3.653414      0.534565     -1.808634
  H      3.701489     -1.985104      1.647619
  H      1.290799     -2.367606      1.391772
  N      5.191270     -0.519835      0.046877
  H      5.610232      0.239038     -0.458753
  H      5.630906     -0.765235      0.914920
  C     -0.333614     -1.402989     -0.467478
  O     -0.954729     -2.117724      0.306316
  O     -0.920499     -0.797249     -1.493653
  H     -1.897882     -0.977698     -1.512351
```

Cartesian coordinates of : ABW8_ 4

Atomic number (AN) and Cartesian coordinates

AN	X	Y	Z
O	-3.533540	-1.661758	1.652878
H	-2.693312	-1.170084	1.577761
H	-4.148270	-1.042912	2.054994
O	-2.717972	1.066096	-1.703453
H	-1.787550	0.898777	-1.880714
H	-3.091287	0.186425	-1.515280
O	-1.150583	-0.279240	1.122443
H	-1.464592	0.641532	1.065920
H	-1.026952	-0.585249	0.205629
O	0.631261	-3.565352	0.126739
H	0.756701	-2.986501	0.902357
H	1.475850	-3.547432	-0.331659
O	0.867936	-1.797567	2.315846
H	1.740877	-1.430225	2.151422
H	0.244700	-1.133292	1.971176
O	-2.388196	2.256428	0.840519
H	-1.689130	2.920919	0.753935
H	-2.560430	1.952144	-0.069122
O	-0.837120	-1.653597	-1.370398
H	-0.395378	-2.386800	-0.906953
H	-1.786018	-1.849082	-1.359906
O	-3.665278	-1.531769	-1.117499
H	-3.770860	-1.633144	-0.151668
H	-4.424563	-1.956343	-1.522832
C	2.084324	0.391535	-0.392084
C	2.235457	1.044983	0.830181
C	1.595805	2.236225	1.073623
C	0.785698	2.816234	0.094008
C	0.647737	2.171609	-1.139694
C	1.286761	0.979654	-1.372448
H	2.856981	0.612698	1.597550
H	1.711489	2.734070	2.024148
H	0.029761	2.619991	-1.903009
H	1.169276	0.483852	-2.322529
N	0.080868	3.961994	0.358429
H	0.407491	4.518973	1.129370
H	-0.210098	4.500065	-0.440187
C	2.717993	-0.891936	-0.675501
O	2.619836	-1.498672	-1.724761
O	3.442807	-1.377587	0.343062
H	3.813369	-2.228024	0.069092

Cartesian coordinates of : ABW8_ 5

Atomic number (AN) and Cartesian coordinates

AN	X	Y	Z
O	0.469579	1.895093	-0.644402
H	0.372993	1.325558	0.145425
H	-0.411047	2.248362	-0.820230
O	1.297760	-2.197605	0.124239
H	1.257357	-1.685507	-0.707391
H	0.559992	-2.819070	0.098242
O	4.121588	0.243708	-1.456807
H	3.888391	1.096023	-1.034849
H	4.837266	0.431688	-2.068395
O	3.974402	-1.418020	0.834054
H	3.109844	-1.828705	0.684318
H	4.146222	-0.922636	0.015158
O	3.214145	2.475502	-0.073776
H	3.245006	2.029801	0.789696
H	2.276925	2.448414	-0.321376
O	1.431162	-0.443926	-2.059314
H	2.379247	-0.242762	-1.985494
H	0.995794	0.339582	-1.685494
O	0.499655	0.165891	1.546996
H	1.355552	0.437075	1.918393
H	0.689068	-0.682249	1.110394
O	3.174041	0.839832	2.239701
H	3.532672	0.019127	1.844703
H	3.589120	0.930234	3.100453
C	-2.715026	0.439321	-0.101390
C	-3.054029	-0.385932	0.969422
C	-2.703299	-1.714730	0.968480
C	-2.010876	-2.262709	-0.115564
C	-1.711611	-1.444306	-1.210389
C	-2.047649	-0.115631	-1.192752
H	-3.575449	0.024276	1.818798
H	-2.947651	-2.344114	1.810498
H	-1.187359	-1.865055	-2.054814
H	-1.789074	0.514412	-2.028972
N	-1.565786	-3.556047	-0.085316
H	-1.988263	-4.165706	0.592896
H	-1.380976	-3.989324	-0.973698
C	-2.973692	1.872679	-0.090353
O	-2.483193	2.668817	-0.873362
O	-3.800949	2.273784	0.881736
H	-3.883549	3.236155	0.831716

Cartesian coordinates of : ABW8_ 6

Atomic number (AN) and Cartesian coordinates

AN	X	Y	Z
O	-0.469519	-1.895122	-0.644278
H	-0.372963	-1.325568	0.145537

H	0.411141	-2.248295	-0.820128
O	-1.297871	2.197522	0.124167
H	-1.257439	1.685403	-0.707449
H	-0.560100	2.818980	0.098191
O	-4.121623	-0.243902	-1.456901
H	-3.888388	-1.096157	-1.034838
H	-4.837183	-0.432022	-2.068585
O	-3.974565	1.417986	0.833871
H	-3.109988	1.828618	0.684100
H	-4.146395	0.922533	0.015020
O	-3.214116	-2.475472	-0.073568
H	-3.244966	-2.029696	0.789870
H	-2.276899	-2.448400	-0.321176
O	-1.431178	0.443758	-2.059312
H	-2.379265	0.242591	-1.985501
H	-0.995817	-0.339719	-1.685415
O	-0.499617	-0.165781	1.547054
H	-1.355465	-0.436960	1.918559
H	-0.689131	0.682284	1.110341
O	-3.174032	-0.839665	2.239797
H	-3.532723	-0.019039	1.844690
H	-3.589451	-0.930209	3.100370
C	2.715079	-0.439282	-0.101410
C	3.054112	0.385995	0.969374
C	2.703340	1.714781	0.968434
C	2.010843	2.262726	-0.115580
C	1.711558	1.444302	-1.210385
C	2.047637	0.115639	-1.192749
H	3.575585	-0.024188	1.818728
H	2.947716	2.344183	1.810432
H	1.187264	1.865031	-2.054793
H	1.789046	-0.514420	-2.028951
N	1.565716	3.556046	-0.085326
H	1.988177	4.165726	0.592875
H	1.380834	3.989312	-0.973698
C	2.973777	-1.872632	-0.090361
O	2.483229	-2.668799	-0.873313
O	3.801118	-2.273701	0.881670
H	3.883720	-3.236073	0.831671

Cartesian coordinates of :   ABW8_ 7

Atomic number (AN) and Cartesian coordinates

AN	X	Y	Z
O	2.860389	-1.207877	2.073626
H	3.758055	-1.371327	1.716095
H	2.733008	-1.834263	2.789780
O	3.940917	-1.805397	-1.698856
H	3.041525	-1.736762	-1.332828
H	3.914660	-2.518108	-2.341787
O	1.654449	1.776079	-0.604879

```
  H      2.035347      1.803136      0.293472
  H      0.706334      1.600203     -0.490751
  O      4.034181      1.044267     -2.039324
  H      4.040359      0.074056     -2.065446
  H      3.158855      1.274208     -1.690873
  O      3.014447      1.654778      1.857212
  H      3.886364      1.672457      1.428283
  H      2.885741      0.719977      2.081439
  O      1.548824     -1.100661     -0.370992
  H      1.700225     -0.158134     -0.557355
  H      1.912613     -1.227283      0.527678
  O      5.297453     -1.426638      0.783848
  H      5.475442     -0.475234      0.694414
  H      4.937189     -1.679265     -0.081707
  O      5.408026      1.381159      0.353717
  H      5.005364      1.339183     -0.537557
  H      6.173578      1.955318      0.276923
  C     -3.167956     -0.001566     -0.101478
  C     -3.868266     -1.204057     -0.003320
  C     -5.237697     -1.217049      0.093215
  C     -5.962947     -0.018437      0.095287
  C     -5.259122      1.189742     -0.000829
  C     -3.890626      1.191110     -0.097690
  H     -3.329367     -2.137379     -0.001061
  H     -5.770107     -2.152961      0.169949
  H     -5.808456      2.118979      0.003749
  H     -3.360339      2.127495     -0.169263
  N     -7.320817     -0.028252      0.148117
  H     -7.773175     -0.870272      0.454338
  H     -7.787710      0.827426      0.387100
  C     -1.712350      0.041996     -0.203036
  O     -1.075437      1.084117     -0.295807
  O     -1.127846     -1.147216     -0.187398
  H     -0.139213     -1.065007     -0.253482
 ---------------------------------------------------

Cartesian coordinates of :    ABW8_ 8

 ---------------------------------------------------
 Atomic number (AN) and Cartesian coordinates
  AN          X             Y             Z
 ---------------------------------------------------
  O     -2.318527      2.260881      0.377373
  H     -2.576135      3.131275      0.692872
  H     -1.430258      2.378775     -0.013504
  O     -4.797949     -1.037415      0.345581
  H     -4.722223     -0.272535     -0.257211
  H     -5.275944     -1.705619     -0.153127
  O      0.320596      2.569627     -0.340514
  H      0.717782      2.205100      0.467713
  H      0.972468      2.395475     -1.039213
  O     -1.342531      0.641002      2.482553
  H     -1.523949     -0.244998      2.151050
  H     -1.741710      1.225700      1.812827
```

```
  O     2.576184      2.134746     -2.040640
  H     3.030515      1.875593     -1.216433
  H     2.889330      3.023738     -2.225862
  O     3.690681      1.434104      0.462823
  H     4.009355      0.522650      0.396569
  H     2.884240      1.369987      0.996975
  O    -4.090462      1.019183     -1.425751
  H    -3.550990      1.557445     -0.819515
  H    -3.463434      0.341277     -1.716977
  O     1.301054      1.443892      2.106932
  H     1.374849      2.186606      2.711963
  H     0.411734      1.068198      2.267948
  C    -0.049791     -1.416382     -0.357812
  C     0.527158     -1.852345      0.833578
  C     1.892781     -1.939646      0.963321
  C     2.726004     -1.589657     -0.101533
  C     2.148648     -1.166678     -1.302166
  C     0.784484     -1.079976     -1.422004
  H    -0.101875     -2.123467      1.665994
  H     2.333265     -2.270956      1.891292
  H     2.788674     -0.894102     -2.127115
  H     0.346796     -0.743421     -2.348199
  N     4.090078     -1.577842      0.049779
  H     4.458444     -2.126148      0.808005
  H     4.630736     -1.638041     -0.796305
  C    -1.497761     -1.299538     -0.527632
  O    -2.026045     -0.968009     -1.579205
  O    -2.201184     -1.587393      0.558542
  H    -3.175900     -1.437248      0.405739
 ---------------------------------------------------

Cartesian coordinates of :    ABW8_ 9

 ---------------------------------------------------
 Atomic number (AN) and Cartesian coordinates
  AN         X             Y             Z
 ---------------------------------------------------
  O    -2.691294      2.251264      1.485879
  H    -2.092277      2.675803      2.104703
  H    -2.337506      2.432213      0.595052
  O    -4.197934      0.691981     -1.490349
  H    -4.592993      0.761499     -0.598112
  H    -4.781931      1.185491     -2.072249
  O    -1.150383     -1.237441     -1.624830
  H    -1.365667     -0.843899     -0.762531
  H    -1.147560     -0.493582     -2.233465
  O    -3.894039     -2.105014     -1.687211
  H    -4.167172     -1.172253     -1.731047
  H    -2.927377     -2.033962     -1.744451
  O    -1.882823     -0.454278      1.040997
  H    -2.180240      0.445044      1.259357
  H    -2.641500     -1.048139      1.187242
  O    -1.780244      2.165190     -1.169976
  H    -1.045990      1.595420     -0.901583
```

```
  H     -2.533105      1.574465     -1.344793
  O     -5.108478      0.722065      1.152243
  H     -4.346256      1.233191      1.460591
  H     -4.868370     -0.205931      1.294807
  O     -4.192533     -2.005247      1.084329
  H     -4.269480     -2.854281      1.525465
  H     -4.138817     -2.191396      0.125631
  C      2.773949      0.047965      0.238857
  C      3.495152     -0.999828      0.811223
  C      4.854049     -1.096625      0.640106
  C      5.546668     -0.140528     -0.114315
  C      4.821390      0.910869     -0.691335
  C      3.463802      0.997208     -0.514619
  H      2.980502     -1.747395      1.392563
  H      5.403105     -1.912895      1.084694
  H      5.344945      1.650530     -1.277901
  H      2.915858      1.809251     -0.965725
  N      6.897515     -0.207339     -0.250009
  H      7.347252     -1.080169     -0.041940
  H      7.321526      0.335248     -0.980283
  C      1.328677      0.173914      0.396360
  O      0.669329      1.073784     -0.106202
  O      0.771929     -0.773980      1.141082
  H     -0.209910     -0.647318      1.168678
 ---------------------------------------------------

Cartesian coordinates of :    ABW8_10

 ---------------------------------------------------
 Atomic number (AN) and Cartesian coordinates
  AN         X             Y             Z
 ---------------------------------------------------
  O     -2.068449      0.195716     -2.088028
  H     -3.022152      0.040703     -2.190793
  H     -2.019911      0.967895     -1.499463
  O     -2.063580      0.369059      1.979280
  H     -1.625937      0.427685      2.831784
  H     -3.003933      0.180136      2.160668
  O     -5.051779      1.784830     -0.014473
  H     -5.064982      1.172456      0.739689
  H     -4.130089      2.087318     -0.057659
  O     -4.767014     -0.232848      1.967971
  H     -4.713280     -0.997040      1.358217
  H     -5.323207     -0.507129      2.700775
  O     -4.883577     -0.203777     -1.944982
  H     -5.554519     -0.133964     -2.627978
  H     -5.037167      0.543307     -1.330943
  O     -2.232123      2.229748     -0.101554
  H     -2.042041      1.691554      0.688348
  H     -1.682986      3.014055     -0.028432
  O     -1.513691     -1.559289      0.011491
  H     -1.655519     -0.948332      0.759184
  H     -1.689312     -1.005488     -0.777817
  O     -4.379864     -2.178625      0.021099
```

```
  H     -3.411000     -2.142933      0.028959
  H     -4.630811     -1.600881     -0.719956
  C      3.070120     -0.140930     -0.011979
  C      3.915681     -1.249914     -0.017317
  C      5.280477     -1.096816     -0.011852
  C      5.853977      0.180949      0.000825
  C      5.004452      1.295436      0.001744
  C      3.642009      1.130448     -0.004232
  H      3.495797     -2.242440     -0.028178
  H      5.925935     -1.962106     -0.017666
  H      5.435386      2.285223      0.006217
  H      2.997891      1.995668     -0.004864
  N      7.204655      0.334920      0.054072
  H      7.773535     -0.452999     -0.197333
  H      7.582376      1.233744     -0.184669
  C      1.614160     -0.270934     -0.015650
  O      0.845686      0.678535     -0.013839
  O      1.176439     -1.526935     -0.019578
  H      0.181714     -1.531619     -0.014931
 ---------------------------------------------------
```

Cartesian coordinates of :   ABW8_11

```
 ---------------------------------------------------
 Atomic number (AN) and Cartesian coordinates
  AN          X             Y             Z
 ---------------------------------------------------
  O     -4.003238      1.263804      1.074276
  H     -3.171884      1.713881      0.845548
  H     -4.554159      1.917954      1.510264
  O     -1.941900      0.527380     -2.148802
  H     -1.707453      1.098409     -1.397685
  H     -2.837069      0.213503     -1.928501
  O     -0.374344     -0.359964      1.379654
  H     -1.202674     -0.658259      1.808425
  H      0.293740     -0.312984      2.067893
  O     -0.665009     -1.775227     -1.077257
  H     -1.035399     -1.005102     -1.547634
  H     -0.364933     -1.397766     -0.234845
  O     -2.908650     -1.112122      2.224553
  H     -3.045651     -1.746213      1.501024
  H     -3.356537     -0.303050      1.926736
  O     -1.429893      1.974110      0.272598
  H     -0.759309      2.665655      0.172645
  H     -0.966115      1.225888      0.695172
  O     -4.462398     -0.421469     -1.188959
  H     -4.100850     -1.258852     -0.854603
  H     -4.431191      0.177382     -0.425948
  O     -3.105443     -2.683714     -0.129735
  H     -2.220106     -2.489314     -0.500350
  H     -3.262086     -3.619553     -0.274511
  C      2.889008     -0.283599     -0.082229
  C      3.005977      0.601867      0.987821
  C      2.447709      1.856491      0.923782
```

C	1.752758	2.263209	-0.217911
C	1.636903	1.376250	-1.292674
C	2.200928	0.127042	-1.222205
H	3.538247	0.303949	1.876171
H	2.540206	2.538152	1.755457
H	1.098103	1.685072	-2.175605
H	2.105135	-0.553744	-2.052569
N	1.126404	3.484720	-0.258629
H	1.457099	4.172582	0.396389
H	0.945548	3.855969	-1.176131
C	3.448885	-1.631072	-0.045123
O	3.369154	-2.441125	-0.948296
O	4.077954	-1.927253	1.101974
H	4.409640	-2.833075	1.034250

Cartesian coordinates of :   ABW8_12

Atomic number (AN) and Cartesian coordinates

AN	X	Y	Z
O	-0.061435	-2.579736	0.217449
H	0.058380	-2.360963	1.158251
H	-1.018792	-2.559897	0.076973
O	2.317138	-0.258575	1.447955
H	1.710683	0.235657	0.885708
H	2.537910	-1.053754	0.924549
O	1.644512	2.377747	-0.943346
H	1.202001	1.518754	-1.058693
H	1.187335	2.793912	-0.199416
O	4.153387	1.307683	-0.356544
H	3.329792	1.795034	-0.552033
H	3.914359	0.787342	0.420704
O	0.666041	-0.253330	-1.487009
H	0.412690	-0.943344	-0.853819
H	1.552846	-0.501295	-1.793701
O	3.416691	-0.739237	-2.120189
H	3.798270	-0.007808	-1.597273
H	3.892356	-0.758388	-2.953513
O	2.819595	-2.582689	-0.095624
H	1.874166	-2.746874	-0.209291
H	3.085131	-2.084141	-0.886567
O	0.499031	-1.834321	2.927365
H	1.148726	-1.215557	2.547726
H	1.025733	-2.539782	3.311817
C	-2.490108	0.090764	-0.135042
C	-2.512014	1.283639	-0.855879
C	-1.822617	2.383402	-0.407943
C	-1.091740	2.325437	0.783002
C	-1.091894	1.137533	1.521095
C	-1.781589	0.042274	1.063976
H	-3.057459	1.340846	-1.783503
H	-1.826955	3.299422	-0.978586

H	-0.543461	1.090193	2.449781
H	-1.780891	-0.869384	1.638501
N	-0.326979	3.391977	1.176792
H	-0.588352	4.291042	0.809747
H	-0.040733	3.407945	2.140515
C	-3.136630	-1.119405	-0.624656
O	-2.954297	-2.236201	-0.168408
O	-3.964162	-0.919820	-1.655497
H	-4.317361	-1.776829	-1.931964

Cartesian coordinates of : ABW8_13

Atomic number (AN) and Cartesian coordinates

AN	X	Y	Z
O	3.322057	-0.719535	-2.504587
H	3.836592	-0.338853	-3.220636
H	2.559310	-0.122194	-2.391161
O	4.688157	-0.688653	-0.080044
H	4.272196	-0.667150	-0.964489
H	4.962666	0.217431	0.084047
O	2.384105	2.167688	0.216491
H	2.252264	1.301809	0.640995
H	1.690787	2.736221	0.581781
O	2.103096	-0.547180	1.031259
H	1.570270	-0.898071	0.294813
H	3.027576	-0.706898	0.765376
O	0.527083	-1.764456	-1.034131
H	0.128824	-2.400941	-0.407968
H	1.177409	-2.260538	-1.538650
O	1.059230	0.912366	-1.991974
H	1.449468	1.422819	-1.261374
H	0.625421	0.160608	-1.564989
O	0.640592	-1.946662	2.969878
H	1.200335	-2.563457	3.448142
H	1.242756	-1.469038	2.369835
O	-0.679676	-3.376744	0.900446
H	-0.296627	-2.907836	1.664064
H	-1.525893	-2.940308	0.725111
C	-2.289517	0.271866	-0.161319
C	-2.379653	1.515987	-0.784210
C	-1.687004	2.597288	-0.295211
C	-0.874800	2.464809	0.833969
C	-0.783589	1.217416	1.459475
C	-1.482875	0.144321	0.968432
H	-2.999828	1.636125	-1.657064
H	-1.761541	3.557959	-0.781354
H	-0.156445	1.106938	2.330997
H	-1.412108	-0.808562	1.465556
N	-0.123163	3.518689	1.287596
H	-0.400725	4.432926	0.974007
H	0.145367	3.486200	2.256696

```
  C     -3.011622     -0.895642     -0.652298
  O     -2.981554     -2.003284     -0.144021
  O     -3.748520     -0.660699     -1.743978
  H     -4.188111     -1.485000     -1.994657
 ---------------------------------------------------

Cartesian coordinates of :    ABW8_14

 ---------------------------------------------------
 Atomic number (AN) and Cartesian coordinates
  AN          X             Y             Z
 ---------------------------------------------------
  O      1.652580      1.368035     -0.351760
  H      1.723837      0.422146     -0.126563
  H      0.695261      1.515204     -0.281394
  O      3.492976     -1.953031     -1.581868
  H      4.236744     -1.862442     -0.949914
  H      3.623857     -2.788912     -2.035706
  O      5.483919      1.319510     -0.546709
  H      4.937324      1.638247      0.189156
  H      4.849341      1.184721     -1.272711
  O      5.412931     -1.376468      0.323963
  H      5.539746     -0.456136      0.026732
  H      4.833869     -1.292661      1.097927
  O      3.604366      1.905856      1.539177
  H      3.510732      2.692624      2.081392
  H      2.831155      1.890335      0.942876
  O      3.465589      0.757466     -2.500364
  H      3.476631     -0.199597     -2.337447
  H      2.757333      1.081534     -1.922701
  O      1.579678     -1.356358      0.382189
  H      2.161758     -1.243238      1.154794
  H      2.153657     -1.686158     -0.332268
  O      3.478294     -0.761970      2.355244
  H      3.521346      0.206183      2.245779
  H      3.442786     -0.924947      3.300683
  C     -3.197457      0.056211      0.037288
  C     -3.867743     -1.162076      0.151543
  C     -5.238478     -1.219896      0.104239
  C     -5.995756     -0.052758     -0.061456
  C     -5.322310      1.171389     -0.174030
  C     -3.952329      1.217726     -0.124522
  H     -3.305122     -2.071945      0.282069
  H     -5.747504     -2.167352      0.196589
  H     -5.896050      2.077137     -0.299026
  H     -3.446069      2.166009     -0.210795
  N     -7.350088     -0.110237     -0.151728
  H     -7.808074     -0.945157      0.165305
  H     -7.865949      0.742057     -0.030422
  C     -1.742170      0.151624      0.085518
  O     -1.133162      1.209520      0.007362
  O     -1.128336     -1.018367      0.223410
  H     -0.149199     -0.932854      0.261113
 ---------------------------------------------------
```

Cartesian coordinates of : ABW8_15

Atomic number (AN) and Cartesian coordinates

AN	X	Y	Z
O	0.464403	-0.148361	1.542398
H	1.290839	-0.494873	1.915230
H	0.725129	0.690849	1.125584
O	3.341837	-2.525391	-0.126198
H	3.687196	-3.416976	-0.040166
H	3.325196	-2.150894	0.774722
O	4.273591	-0.192306	-1.471120
H	4.068271	-1.059368	-1.084808
H	3.426835	0.105125	-1.840469
O	1.598623	0.575921	-2.090781
H	1.191320	-0.242453	-1.738376
H	1.110798	0.791859	-2.889451
O	1.383762	2.215063	0.183850
H	1.378548	1.751395	-0.673583
H	0.602777	2.787799	0.178898
O	0.688146	-1.721942	-0.815090
H	1.557095	-2.114596	-0.628244
H	0.473606	-1.227140	-0.002930
O	3.088200	-1.058264	2.218093
H	3.493847	-1.228518	3.071490
H	3.486350	-0.227200	1.883638
O	3.992037	1.231307	0.965563
H	4.161412	0.791042	0.111446
H	3.155700	1.698637	0.828544
C	-2.861637	-0.404611	-0.026485
C	-2.162531	0.066784	-1.136442
C	-1.692343	1.356936	-1.171495
C	-1.904432	2.217434	-0.090852
C	-2.618213	1.750681	1.016063
C	-3.086723	0.460025	1.042491
H	-1.984441	-0.584326	-1.975977
H	-1.145746	1.715079	-2.030571
H	-2.791457	2.413251	1.850188
H	-3.633947	0.104145	1.900865
N	-1.356701	3.477204	-0.092944
H	-1.189936	3.882710	-0.998709
H	-1.740041	4.126025	0.573149
C	-3.355532	-1.775860	0.056066
O	-3.977446	-2.235381	0.994346
O	-3.058714	-2.524444	-1.016486
H	-3.419488	-3.409959	-0.872522

Cartesian coordinates of : ABW8_16

Atomic number (AN) and Cartesian coordinates

AN	X	Y	Z
O	4.214064	-0.204874	-1.453419
H	4.024219	-1.079903	-1.066023
H	4.954397	-0.329265	-2.051714
O	3.097567	-0.932250	2.194944
H	3.481210	-0.103065	1.841136
H	3.530510	-1.092313	3.036845
O	1.332985	2.193644	0.126259
H	1.314427	1.673586	-0.701281
H	0.573880	2.791927	0.087040
O	0.427977	-0.097913	1.605671
H	0.665374	0.719485	1.135052
H	1.274540	-0.422254	1.952434
O	1.503249	0.444506	-2.055301
H	1.075367	-0.332909	-1.655780
H	2.452864	0.254008	-1.980993
O	3.972643	1.358416	0.901063
H	3.118587	1.785516	0.737692
H	4.173424	0.901852	0.066401
O	3.285987	-2.458815	-0.109689
H	3.740431	-3.300631	-0.027385
H	3.293127	-2.057292	0.780589
O	0.519534	-1.861930	-0.629403
H	1.416957	-2.202224	-0.488049
H	0.360095	-1.292038	0.144723
C	-2.832486	-0.407886	-0.050481
C	-2.136281	0.062231	-1.163041
C	-1.701269	1.363300	-1.218151
C	-1.943164	2.236889	-0.154072
C	-2.657561	1.772313	0.953681
C	-3.092839	0.470451	0.999151
H	-1.926990	-0.600759	-1.986075
H	-1.153431	1.719131	-2.077183
H	-2.854885	2.444626	1.774581
H	-3.637493	0.115214	1.859511
N	-1.420143	3.505719	-0.172379
H	-1.247812	3.899861	-1.081920
H	-1.814881	4.158359	0.482973
C	-3.275571	-1.793999	0.058073
O	-3.894163	-2.254967	0.998218
O	-2.932278	-2.555712	-0.990504
H	-3.258398	-3.451585	-0.829614

Cartesian coordinates of : ABW8_17

Atomic number (AN) and Cartesian coordinates

AN	X	Y	Z
O	2.099696	1.955094	-1.768521
H	2.401498	2.646045	-2.362973
H	1.413118	2.366382	-1.205293

```
  O      4.472414    -1.020189     0.550046
  H      5.274505    -1.428203     0.883639
  H      3.741926    -1.325932     1.123737
  O      3.086105    -0.779949    -1.940251
  H      3.648181    -0.875155    -1.156330
  H      2.715134     0.111966    -1.874148
  O      2.151545    -1.667630     1.926255
  H      1.781133    -0.768159     1.937841
  H      1.726269    -2.113476     1.180088
  O      0.383732     3.063339     0.095781
  H      0.525681     2.414054     0.801309
  H     -0.579825     3.144887    -0.013244
  O      1.427482     1.115647     1.982851
  H      2.278674     1.361613     1.563801
  H      1.485110     1.438275     2.886044
  O      3.748965     1.709060     0.571541
  H      3.305118     1.815490    -0.286152
  H      4.121525     0.811411     0.553177
  O     -2.401333     3.477983    -0.306859
  H     -2.467745     3.583033    -1.259496
  H     -2.908457     2.673844    -0.111607
  C     -2.641504    -0.755988    -0.040046
  C     -2.590004    -2.147600    -0.125000
  C     -1.402070    -2.791689    -0.372127
  C     -0.221417    -2.061110    -0.544893
  C     -0.274374    -0.664642    -0.463550
  C     -1.463497    -0.031317    -0.213718
  H     -3.489417    -2.727385     0.002372
  H     -1.368553    -3.868598    -0.436671
  H      0.633549    -0.095270    -0.588423
  H     -1.488473     1.042503    -0.147127
  N      0.979844    -2.686670    -0.727605
  H      0.947745    -3.654204    -0.997838
  H      1.705089    -2.140153    -1.175011
  C     -3.879817    -0.037908     0.224941
  O     -3.985372     1.175128     0.303771
  O     -4.947034    -0.828406     0.388036
  H     -5.714193    -0.265367     0.560694
 ----------------------------------------------------

Cartesian coordinates of :    ABW8_18

 ----------------------------------------------------
 Atomic number (AN) and Cartesian coordinates
  AN         X            Y            Z
 ----------------------------------------------------
  O      2.934279    -2.739862    -1.849820
  H      3.523544    -2.265194    -1.239582
  H      3.060584    -2.303566    -2.696659
  O      2.530616     2.519957     0.320165
  H      2.692475     2.016788    -0.496584
  H      1.649635     2.914591     0.207033
  O     -0.680608    -2.636761     1.222229
  H     -1.613724    -2.440925     1.062078
```

```
  H     -0.209350     -2.188350      0.495650
  O      3.041174      0.747665     -1.869702
  H      2.180525      0.314328     -1.866588
  H      3.591167      0.163261     -1.322622
  O      0.217467     -0.907695      3.249887
  H     -0.141684     -1.537256      2.594109
  H      0.678487     -1.453073      3.892283
  O      0.817993     -1.196171     -0.706454
  H      1.466447     -1.784861     -1.129864
  H      1.312869     -0.755766      0.008840
  O      4.272766     -1.080894     -0.018223
  H      5.144511     -1.015589      0.378086
  H      3.637156     -0.796426      0.661999
  O      2.193048     -0.015378      1.473012
  H      2.185083      0.933517      1.255867
  H      1.514977     -0.189103      2.151018
  C     -2.494938      0.161517     -0.327197
  C     -2.231288      1.013708     -1.397941
  C     -1.479554      2.150164     -1.219274
  C     -0.977936      2.474358      0.043356
  C     -1.229820      1.613311      1.115077
  C     -1.980199      0.479254      0.928428
  H     -2.616183      0.781109     -2.377172
  H     -1.280366      2.808860     -2.050827
  H     -0.839322      1.856509      2.091449
  H     -2.181933     -0.173334      1.762277
  N     -0.207720      3.599185      0.222474
  H     -0.306419      4.311453     -0.481513
  H     -0.189707      3.967208      1.159393
  C     -3.274577     -1.060717     -0.483566
  O     -3.413017     -1.911393      0.379335
  O     -3.847627     -1.193537     -1.684002
  H     -4.323577     -2.035496     -1.700697
 ---------------------------------------------------

Cartesian coordinates of :    ABW8_19

 ---------------------------------------------------
 Atomic number (AN) and Cartesian coordinates
  AN         X            Y            Z
 ---------------------------------------------------
  O      1.771695      2.020988     -2.063725
  H      2.530602      1.904594     -1.458654
  H      2.113368      2.505559     -2.819146
  O      3.906437     -0.262830      2.872453
  H      4.620840      0.208414      2.434898
  H      3.099382      0.156011      2.533172
  O      0.870924     -1.736132      0.092345
  H      1.075960     -1.423849     -0.813370
  H     -0.080247     -1.868978      0.119182
  O      3.456745     -2.460301      1.084807
  H      3.644002     -1.763422      1.737261
  H      2.521882     -2.340913      0.859348
  O      1.781491     -0.829297     -2.362771
```

```
  H      2.705985     -0.996504     -2.114263
  H      1.700765      0.139402     -2.353015
  O      1.559052      0.581617      1.529913
  H      0.975817      1.301514      1.239961
  H      1.253326     -0.209600      1.047833
  O      3.733847      1.364101     -0.187003
  H      4.057534      0.527886     -0.562028
  H      3.044196      1.094614      0.443436
  O      4.352587     -1.209597     -1.221828
  H      4.132188     -1.729080     -0.421733
  H      5.185348     -1.556295     -1.549766
  C     -3.510396     -0.086020      0.056454
  C     -2.445734     -0.235148     -0.831315
  C     -1.384978      0.637872     -0.807905
  C     -1.354226      1.691603      0.111048
  C     -2.424820      1.844715      0.997950
  C     -3.481358      0.969099      0.966874
  H     -2.450567     -1.041529     -1.546292
  H     -0.562947      0.515073     -1.495886
  H     -2.408809      2.657744      1.707747
  H     -4.303186      1.093809      1.653899
  N     -0.264357      2.519356      0.180715
  H      0.331629      2.551411     -0.634624
  H     -0.402990      3.403368      0.638977
  C     -4.654204     -0.992296      0.059904
  O     -5.607767     -0.906496      0.809735
  O     -4.578035     -1.966431     -0.859514
  H     -5.375090     -2.508650     -0.785289
 ---------------------------------------------------

Cartesian coordinates of :    ABW8_20

 ---------------------------------------------------
 Atomic number (AN) and Cartesian coordinates
  AN          X             Y             Z
 ---------------------------------------------------
  O     -0.938991      1.246867      0.350948
  H     -0.796753      0.282114      0.310543
  H     -0.068472      1.650264      0.493237
  O     -3.184975     -1.576882      1.927228
  H     -3.245372     -0.607311      2.052348
  H     -3.329821     -1.971484      2.790198
  O     -4.929991      1.234733     -0.425275
  H     -4.172637      1.373329     -1.030471
  H     -5.606299      1.862933     -0.688706
  O     -4.826870     -1.602761     -0.400016
  H     -4.337842     -1.711477      0.432662
  H     -5.020208     -0.651172     -0.435241
  O     -2.608028      1.347324     -1.940304
  H     -2.598278      0.401628     -2.161595
  H     -1.978141      1.421598     -1.202549
  O     -3.296923      1.193177      1.911242
  H     -3.948047      1.291750      1.197001
  H     -2.440810      1.315731      1.465791
```

O	-0.948443	-1.564786	0.176188
H	-1.427638	-1.626562	-0.666494
H	-1.640633	-1.675506	0.848900
O	-2.649351	-1.493932	-2.113694
H	-3.465267	-1.620843	-1.586768
H	-2.706529	-2.106454	-2.850734
C	3.031798	0.818031	0.107455
C	4.171905	0.440662	-0.605066
C	4.564899	-0.872280	-0.659389
C	3.829448	-1.864864	0.004299
C	2.689064	-1.484247	0.725967
C	2.303440	-0.169857	0.771825
H	4.751132	1.187233	-1.123392
H	5.446268	-1.155407	-1.214605
H	2.118519	-2.239605	1.244498
H	1.429976	0.108798	1.338879
N	4.192650	-3.168202	-0.079392
H	5.115537	-3.385148	-0.408091
H	3.789743	-3.817964	0.570384
C	2.585633	2.198795	0.176849
O	1.550441	2.577353	0.703913
O	3.419594	3.075782	-0.395649
H	3.034190	3.958520	-0.308346

Cartesian coordinates of :   ABW8_21

Atomic number (AN) and Cartesian coordinates

AN	X	Y	Z
O	2.744175	2.264952	-1.189948
H	3.154801	2.288728	-0.302257
H	3.024926	3.070282	-1.630801
O	3.089670	-2.336328	-1.245595
H	3.529405	-1.528975	-1.580743
H	3.432288	-3.057698	-1.778479
O	3.903522	2.022327	1.332948
H	3.254600	1.422087	1.734754
H	4.645575	1.439273	1.101838
O	5.778659	0.038797	0.525218
H	6.697665	-0.092878	0.769091
H	5.282782	-0.720456	0.894816
O	4.067695	-1.964390	1.394656
H	3.730144	-2.201431	0.512892
H	3.386120	-1.379299	1.764244
O	-7.941154	-0.481514	0.381252
H	-8.024460	0.467714	0.515126
H	-8.354270	-0.645033	-0.471768
O	4.369422	0.035443	-1.943838
H	4.975976	0.051048	-1.185440
H	3.780298	0.793994	-1.790735
O	2.105072	-0.029735	2.145709
H	1.436406	-0.095993	2.831998

```
  H      1.624592    -0.090605     1.296685
  C     -3.183818     0.122012    -0.116183
  C     -2.563581    -1.123852    -0.180910
  C     -1.200277    -1.223601    -0.330377
  C     -0.412389    -0.073457    -0.420338
  C     -1.033666     1.177226    -0.356975
  C     -2.396089     1.267145    -0.207952
  H     -3.156931    -2.021142    -0.116378
  H     -0.726312    -2.192006    -0.381798
  H     -0.430932     2.069871    -0.426995
  H     -2.868654     2.235390    -0.161799
  N      0.955715    -0.165825    -0.513130
  H      1.324532    -1.040960    -0.852254
  H      1.429002     0.641885    -0.891125
  C     -4.633141     0.264022     0.040857
  O     -5.207858     1.339263     0.093220
  O     -5.289029    -0.888595     0.125569
  H     -6.261186    -0.711741     0.218779
 ---------------------------------------------------

Cartesian coordinates of :    ABW8_22

 ---------------------------------------------------
 Atomic number (AN) and Cartesian coordinates
  AN          X             Y             Z
 ---------------------------------------------------
  O      3.671820    -2.264874     1.004565
  H      4.186832    -1.483706     0.739660
  H      2.962012    -1.905875     1.559873
  O      3.269872     1.390389     2.053896
  H      2.676893     0.655018     2.279217
  H      3.875938     1.017983     1.390355
  O      1.613837     3.086503     0.631003
  H      2.017158     3.957640     0.655820
  H      2.218210     2.507302     1.140047
  O      1.522369     2.224012    -2.003506
  H      0.597375     2.038271    -2.185831
  H      1.540103     2.543269    -1.078491
  O      4.839082     0.145203     0.073493
  H      5.765994     0.305153    -0.117761
  H      4.367685     0.175351    -0.786206
  O      3.329845     0.048138    -2.221479
  H      2.899400    -0.802937    -2.032702
  H      2.638563     0.729665    -2.152757
  O      2.347442    -2.525240    -1.442863
  H      2.725220    -3.234265    -1.968487
  H      2.833729    -2.535406    -0.594606
  O      1.558888    -0.859349     2.338511
  H      0.913278    -1.091773     3.010235
  H      1.055633    -0.724054     1.512431
  C     -3.738361    -0.082673    -0.012756
  C     -3.147489    -1.343688    -0.068621
  C     -1.783901    -1.473933    -0.181286
  C     -0.968219    -0.341401    -0.240275
```

C	-1.560181	0.923599	-0.191853
C	-2.923533	1.045347	-0.079197
H	-3.762216	-2.227589	-0.024621
H	-1.330815	-2.452544	-0.224676
H	-0.935263	1.802120	-0.243582
H	-3.375332	2.023860	-0.042613
N	0.400763	-0.469888	-0.291251
H	0.749441	-1.327205	-0.694521
H	0.892057	0.340213	-0.634779
C	-5.182575	0.095666	0.114479
O	-5.747119	1.171237	0.161222
O	-5.866752	-1.055515	0.182519
H	-6.805951	-0.842794	0.270919

Cartesian coordinates of : ABW8_23

Atomic number (AN) and Cartesian coordinates

AN	X	Y	Z
O	-1.997085	-0.009812	-2.523528
H	-1.669926	0.268580	-3.382241
H	-1.610997	0.611453	-1.871724
O	-3.920458	-1.582334	0.403809
H	-4.256368	-0.859719	-0.166271
H	-4.647938	-1.840645	0.974275
O	-1.642021	-0.399082	1.482427
H	-0.956950	-1.083553	1.494437
H	-2.446342	-0.845402	1.154969
O	-2.702075	1.845611	2.809615
H	-2.233546	1.068139	2.460843
H	-2.011017	2.475632	3.029552
O	-1.183326	1.632843	-0.456087
H	-1.996815	2.138793	-0.302092
H	-1.226457	0.925476	0.212280
O	-1.849635	-2.602537	-1.324339
H	-1.785131	-1.764953	-1.810996
H	-2.584590	-2.450583	-0.713079
O	-4.545189	0.499976	-1.301438
H	-3.759492	0.364174	-1.855294
H	-4.330257	1.274353	-0.755119
O	-3.743343	2.657552	0.355628
H	-4.041857	3.569745	0.362234
H	-3.461398	2.453366	1.269043
C	3.517137	0.114854	-0.049783
C	2.358297	0.128774	-0.825723
C	1.333085	-0.749037	-0.571934
C	1.432735	-1.673195	0.474479
C	2.597161	-1.689653	1.250826
C	3.617044	-0.809845	0.988846
H	2.261828	0.834057	-1.634902
H	0.438413	-0.732201	-1.175136
H	2.682246	-2.402152	2.057273

H	4.512064	-0.829370	1.590303
N	0.385894	-2.502676	0.764067
H	-0.292423	-2.660026	0.028975
H	0.587716	-3.299431	1.342195
C	4.627511	1.028747	-0.290520
O	5.658780	1.056387	0.354160
O	4.426753	1.872287	-1.315057
H	5.210952	2.432007	-1.396678

Cartesian coordinates of : ABW8_24

Atomic number (AN) and Cartesian coordinates

AN	X	Y	Z
O	2.861196	-1.269508	2.059616
H	3.756743	-1.412935	1.693914
H	2.748820	-1.909520	2.764431
O	3.925873	-1.791336	-1.736414
H	3.030707	-1.722984	-1.364565
H	3.889757	-2.489278	-2.392931
O	1.645438	1.801943	-0.567867
H	2.033502	1.796588	0.325611
H	0.702921	1.603933	-0.452920
O	4.022963	1.075760	-2.003552
H	4.030273	0.108126	-2.044292
H	3.151741	1.299372	-1.644797
O	3.027853	1.602551	1.877383
H	3.896667	1.630719	1.446673
H	2.899254	0.665526	2.082973
O	1.545956	-1.097839	-0.384112
H	1.696761	-0.154764	-0.553368
H	1.912820	-1.242050	0.508091
O	5.290642	-1.417922	0.748203
H	5.466911	-0.466776	0.676941
H	4.928993	-1.655942	-0.118734
O	5.426965	1.403026	0.369978
H	5.017330	1.368217	-0.516296
H	6.186265	1.983220	0.293158
C	-3.167075	-0.002003	-0.104065
C	-3.870358	-1.202413	-0.006350
C	-5.239487	-1.211076	0.092357
C	-5.960062	-0.010621	0.097992
C	-5.253708	1.195194	0.002155
C	-3.885586	1.192800	-0.097340
H	-3.335320	-2.136706	-0.006662
H	-5.773979	-2.144659	0.168168
H	-5.799558	2.125257	0.009171
H	-3.353470	2.126946	-0.168503
N	-7.319477	-0.016379	0.153431
H	-7.771912	-0.855963	0.463544
H	-7.781383	0.839112	0.399298
C	-1.712431	0.037085	-0.208038

O	-1.070323	1.076827	-0.305410
O	-1.129308	-1.154053	-0.188758
H	-0.143297	-1.070157	-0.255726

Cartesian coordinates of :   ABW10_ 1

Atomic number (AN) and Cartesian coordinates

AN	X	Y	Z
O	0.194421	-2.258783	-0.364662
H	-0.150944	-1.783618	-1.142510
H	1.126489	-2.430779	-0.557614
O	-1.144129	2.271323	0.631507
H	-0.411445	2.898395	0.560875
H	-1.437557	2.115881	-0.289855
O	-1.114535	-0.854900	-2.405143
H	-0.958204	-1.131186	-3.311800
H	-1.980684	-1.243742	-2.149435
O	-3.539285	1.405672	1.916707
H	-3.233340	0.554605	2.273876
H	-2.728475	1.834648	1.601568
O	-4.449148	0.707174	-0.603113
H	-5.363322	0.970195	-0.732649
H	-4.219825	0.955464	0.316547
O	-0.049682	-0.164101	1.595594
H	0.106425	-0.800756	0.877241
H	-0.362398	0.654454	1.170292
O	-2.391620	-1.082531	2.685736
H	-1.511532	-0.765744	2.384959
H	-2.287987	-1.299558	3.615658
O	-3.484196	-1.826464	-1.416248
H	-3.136048	-2.296277	-0.636065
H	-3.871776	-1.002377	-1.075580
O	-2.264710	-3.174045	0.753996
H	-2.371335	-2.539391	1.482283
H	-1.366844	-3.010647	0.426015
O	-2.149111	1.743820	-1.896677
H	-1.708177	0.917475	-2.156621
H	-3.013573	1.454462	-1.557708
C	3.065515	-0.191192	-0.127776
C	3.422205	0.743617	0.842567
C	2.908331	2.016796	0.810292
C	2.025457	2.398421	-0.203989
C	1.683081	1.466814	-1.191033
C	2.194037	0.194542	-1.145726
H	4.095117	0.463098	1.636176
H	3.176468	2.732932	1.571808
H	1.007008	1.757753	-1.980996
H	1.922191	-0.520844	-1.905397
N	1.451593	3.641852	-0.195037

H	1.911687	4.346033	0.355550
H	1.090228	3.976063	-1.072160
C	3.537384	-1.568207	-0.081715
O	3.089447	-2.477883	-0.761149
O	4.522200	-1.781900	0.796581
H	4.743427	-2.723305	0.780034

Cartesian coordinates of : ABW10_ 2

Atomic number (AN) and Cartesian coordinates

AN	X	Y	Z
O	-2.999298	-3.008972	0.098484
H	-3.627714	-2.680062	-0.574831
H	-2.845016	-3.936079	-0.096086
O	-0.485163	2.304106	-1.370728
H	-1.140837	1.717552	-1.774289
H	0.085155	1.724430	-0.841396
O	-4.727089	-1.673176	-1.631162
H	-4.101320	-1.013601	-1.965497
H	-5.172905	-1.223108	-0.893523
O	-2.293089	2.985815	0.703915
H	-3.139075	2.708338	0.312828
H	-1.646007	2.863706	-0.014129
O	-2.727066	0.480287	-2.109290
H	-3.354413	1.024682	-1.602683
H	-2.228237	-0.023592	-1.439724
O	-5.660641	-0.300777	0.672192
H	-5.010850	-0.629057	1.328897
H	-6.518293	-0.321647	1.103014
O	-4.598061	1.945228	-0.532348
H	-5.271896	2.452033	-0.991483
H	-5.064776	1.215797	-0.080112
O	-3.668237	-1.301594	2.298104
H	-2.961664	-0.636623	2.287145
H	-3.385346	-1.977406	1.662707
O	-1.622565	0.672565	2.033913
H	-0.765814	0.786019	2.453362
H	-1.842380	1.537022	1.624047
O	-1.197869	-0.914137	-0.212623
H	-1.711552	-1.733854	-0.084559
H	-1.333900	-0.380998	0.596569
C	3.493342	-0.113146	-0.017939
C	4.182414	-1.269879	-0.383274
C	5.554099	-1.312892	-0.353328
C	6.293173	-0.191848	0.047437
C	5.600922	0.971726	0.410618
C	4.229949	1.003531	0.376495
H	3.632466	-2.142947	-0.694693
H	6.078286	-2.212368	-0.638906
H	6.161109	1.842684	0.715693
H	3.708296	1.905045	0.656361

```
  N      7.648606     -0.244037      0.121262
  H      8.118493     -0.989103     -0.359701
  H      8.150641      0.623030      0.179933
  C      2.036638     -0.041018     -0.037457
  O      1.403073      0.953844      0.298130
  O      1.433568     -1.146569     -0.447950
  H      0.443583     -1.043454     -0.378181
 ---------------------------------------------------
```

Cartesian coordinates of :   ABW10_ 3

```
 ---------------------------------------------------
 Atomic number (AN) and Cartesian coordinates
  AN          X             Y             Z
 ---------------------------------------------------
  O      0.401194     -2.688106     -1.817537
  H     -0.022574     -1.812671     -1.845554
  H      1.347737     -2.511729     -1.724952
  O     -0.671377      0.372571      1.210407
  H     -1.541355      0.182839      1.602719
  H     -0.675518      1.339689      1.083925
  O     -2.502716      2.240012     -1.563966
  H     -1.951051      1.454874     -1.713752
  H     -3.286928      1.875099     -1.122613
  O     -3.293061     -0.127378      2.186422
  H     -3.612051      0.252509      3.008296
  H     -3.827025      0.268988      1.473111
  O     -4.515603      0.762409     -0.160489
  H     -5.467740      0.880625     -0.204981
  H     -4.330976     -0.091258     -0.596419
  O      0.080760     -2.435809      1.033681
  H      0.061739     -1.470236      1.094004
  H      0.212467     -2.613465      0.086860
  O     -2.735800     -2.729988      1.217278
  H     -1.762862     -2.694086      1.166983
  H     -2.979923     -1.921288      1.695919
  O     -1.097020     -0.283004     -1.550785
  H     -0.889373     -0.093941     -0.616876
  H     -1.940964     -0.768812     -1.515078
  O     -3.540287     -1.628984     -1.178561
  H     -3.290176     -2.134440     -0.376087
  H     -3.933535     -2.257734     -1.788132
  O     -1.100902      3.124205      0.728101
  H     -0.244090      3.472149      0.434148
  H     -1.581621      2.903606     -0.090941
  C      2.817806     -0.209006     -0.004495
  C      3.006523      0.621161      1.100056
  C      2.637839      1.943374      1.054067
  C      2.064367      2.477508     -0.102894
  C      1.853922      1.641637     -1.202861
  C      2.231713      0.322374     -1.151795
  H      3.454588      0.227203      1.997220
  H      2.793389      2.583297      1.909056
  H      1.402449      2.047001     -2.095524
```

H	2.084118	-0.309091	-2.013113
N	1.656969	3.788718	-0.137188
H	2.086688	4.401603	0.534922
H	1.562903	4.196513	-1.052200
C	3.240816	-1.604739	-0.002772
O	3.126509	-2.368886	-0.945740
O	3.795201	-2.005229	1.146046
H	4.039793	-2.936626	1.054689

Cartesian coordinates of : ABW10_ 4

Atomic number (AN) and Cartesian coordinates

AN	X	Y	Z
O	3.826520	0.881471	-1.683897
H	2.939919	1.275693	-1.683778
H	4.095436	0.870622	-0.750779
O	0.337423	-1.016100	-1.079021
H	1.192826	-1.339595	-1.417862
H	0.451161	-1.013324	-0.111923
O	2.946650	-1.744866	-1.795876
H	3.233021	-2.221824	-2.578238
H	3.330236	-0.846267	-1.863256
O	-0.960189	-3.441434	1.759651
H	-1.119786	-3.455397	0.795509
H	-0.444874	-4.231563	1.940489
O	0.838675	-1.284531	1.748288
H	0.213910	-2.031076	1.813590
H	1.710806	-1.674455	1.550954
O	1.634440	1.402454	1.193610
H	1.480129	1.528992	0.247181
H	1.193821	0.565075	1.406293
O	4.274655	0.610681	1.123563
H	3.374371	0.967050	1.267730
H	4.858907	1.113282	1.695914
O	3.431875	-2.110687	0.994523
H	3.839066	-1.243554	1.151898
H	3.312522	-2.136988	0.031419
O	-1.386781	-3.262695	-1.007049
H	-0.804462	-2.489317	-1.118950
H	-2.282265	-2.889788	-0.986788
O	1.076534	1.636327	-1.804667
H	0.446827	2.331205	-1.575013
H	0.633993	0.791022	-1.613870
C	-1.854623	1.717974	0.190173
C	-2.085494	0.789084	1.202473
C	-2.785935	-0.365410	0.945438
C	-3.296118	-0.613093	-0.331604
C	-3.061384	0.317033	-1.348305
C	-2.343096	1.457719	-1.089024
H	-1.702231	0.969327	2.193783
H	-2.956768	-1.085756	1.730379

H	-3.450965	0.127814	-2.336895
H	-2.164494	2.172666	-1.876715
N	-3.959196	-1.785737	-0.600768
H	-4.368040	-2.251874	0.191675
H	-4.550256	-1.780448	-1.414801
C	-1.118821	2.955158	0.428385
O	-0.649678	3.659221	-0.449308
O	-0.996521	3.275982	1.718867
H	-0.464436	4.081263	1.782742

Cartesian coordinates of : ABW10_ 5

Atomic number (AN) and Cartesian coordinates

AN	X	Y	Z
O	-4.765779	-2.351885	-1.093184
H	-4.191291	-2.590913	-0.337302
H	-5.106821	-3.176773	-1.446360
O	-1.194410	-0.616968	0.320617
H	-1.625930	0.133169	0.765138
H	-0.328735	-0.298021	0.021963
O	-6.013300	-0.147101	0.188768
H	-5.725912	-0.940973	-0.292565
H	-5.493100	0.569684	-0.207922
O	-4.103753	1.676301	-0.927738
H	-3.630006	0.980225	-1.424274
H	-4.175152	2.430864	-1.516913
O	-0.649095	1.626011	-2.251894
H	-1.408100	1.044416	-2.369982
H	0.047102	1.052314	-1.897871
O	-4.479346	-0.585534	2.456933
H	-5.126718	-0.422709	1.739948
H	-4.982244	-0.607889	3.274456
O	-3.082701	-2.648608	1.096322
H	-3.570179	-2.044719	1.681914
H	-2.322451	-2.122958	0.806811
O	-2.772383	-0.534520	-2.001060
H	-2.213555	-0.654817	-1.210015
H	-3.485736	-1.184169	-1.893006
O	-0.471022	2.747530	0.322228
H	-1.131603	2.355056	0.909472
H	-0.703054	2.427330	-0.569054
O	-2.757712	1.459239	1.526409
H	-3.254841	1.643147	0.709625
H	-3.306738	0.817630	2.008461
C	3.575881	0.166621	0.010586
C	4.541717	0.812894	0.783416
C	5.825780	0.333889	0.852565
C	6.195050	-0.819073	0.146266
C	5.223807	-1.470285	-0.627383
C	3.943921	-0.982113	-0.690395
H	4.278377	1.700354	1.335348

H	6.565856	0.840514	1.453211
H	5.496899	-2.361763	-1.171478
H	3.205643	-1.493271	-1.287545
N	7.473994	-1.272732	0.179040
H	8.077418	-0.933987	0.905749
H	7.651752	-2.207815	-0.138995
C	2.206453	0.654665	-0.081637
O	1.346458	0.113126	-0.775101
O	1.950351	1.729446	0.640462
H	1.007048	2.039308	0.515309

Cartesian coordinates of : ABW10_ 6

Atomic number (AN) and Cartesian coordinates

AN	X	Y	Z
O	-0.487272	-2.270946	-1.023006
H	-0.954327	-2.454634	-0.188336
H	-0.901828	-1.455387	-1.346936
O	-3.300076	2.699119	-1.291908
H	-2.741005	1.935001	-1.519202
H	-2.999963	3.426109	-1.842569
O	-0.951341	0.174731	1.362710
H	-1.221948	-0.756292	1.451943
H	-0.123164	0.169174	0.855707
O	-5.774479	1.330180	-0.979648
H	-5.715746	1.151220	-0.025940
H	-5.017435	1.911640	-1.158706
O	-4.459792	-1.000135	-1.705369
H	-5.016124	-0.207938	-1.556835
H	-4.762312	-1.394993	-2.526557
O	-2.905515	2.189930	1.502276
H	-2.195598	1.525951	1.459688
H	-3.010450	2.496042	0.587331
O	-5.194881	0.682518	1.738419
H	-4.382720	1.233027	1.742527
H	-5.705690	0.954229	2.504648
O	-1.909445	-2.453481	1.394344
H	-2.877115	-2.321433	1.289961
H	-1.792054	-3.056091	2.132082
O	-4.563602	-1.957565	0.958113
H	-4.787394	-1.095653	1.349865
H	-4.573118	-1.783596	0.002186
O	-1.946285	0.216717	-1.446569
H	-1.805114	0.258702	-0.488607
H	-2.795190	-0.249881	-1.563733
C	3.712254	-0.111467	-0.136276
C	4.713954	-1.070497	-0.288145
C	6.038046	-0.735884	-0.148168
C	6.412014	0.580650	0.152278
C	5.405656	1.544583	0.302751
C	4.085727	1.199129	0.159964

H	4.447529	-2.088280	-0.522241
H	6.806011	-1.484669	-0.270385
H	5.683129	2.562539	0.530783
H	3.320496	1.950173	0.276322
N	7.718602	0.907252	0.333895
H	8.413691	0.271183	-0.011906
H	7.967308	1.879289	0.307638
C	2.297666	-0.438304	-0.282905
O	1.400331	0.392407	-0.184601
O	2.048500	-1.712403	-0.535673
H	1.068877	-1.864023	-0.675698

Cartesian coordinates of : ABW10_ 7

Atomic number (AN) and Cartesian coordinates

AN	X	Y	Z
O	4.481518	-1.975582	-0.761739
H	5.075540	-2.628802	-1.138373
H	4.567122	-1.170319	-1.310892
O	1.839281	-2.076938	0.126720
H	2.716606	-2.191072	-0.279727
H	1.969550	-1.425158	0.833399
O	2.173597	2.100092	0.191047
H	3.133659	2.162681	0.330737
H	2.067709	1.497591	-0.566571
O	-0.646796	2.358470	0.608312
H	0.320785	2.415168	0.609270
H	-0.951222	3.269326	0.588335
O	4.501007	0.478697	-2.073513
H	4.729361	1.005937	-1.290173
H	3.534306	0.521559	-2.125871
O	1.662559	0.204686	-1.869023
H	0.747348	0.532920	-1.971440
H	1.606478	-0.565273	-1.287341
O	-0.884819	1.318182	-2.000135
H	-1.514049	0.591681	-2.003722
H	-0.898300	1.661016	-1.086231
O	4.954018	1.763842	0.431802
H	5.677021	2.305350	0.756588
H	4.945248	0.953689	0.979955
O	1.814043	0.043060	2.113441
H	0.891830	-0.251647	2.055328
H	1.878220	0.794151	1.495849
O	4.644709	-0.658573	1.767848
H	4.636858	-1.207348	0.965734
H	3.712425	-0.551192	2.002681
C	-2.764866	-0.715622	0.327124
C	-3.432602	-1.037128	-0.855271
C	-4.721850	-0.619321	-1.072704
C	-5.394063	0.146008	-0.109965
C	-4.723096	0.467424	1.078395

```
  C     -3.435939     0.041889     1.286650
  H     -2.932504    -1.623605    -1.609269
  H     -5.231768    -0.876337    -1.988840
  H     -5.232723     1.055918     1.826148
  H     -2.927399     0.297896     2.202663
  N     -6.652178     0.601087    -0.340347
  H     -7.181229     0.175936    -1.079569
  H     -7.183446     0.939215     0.440945
  C     -1.389265    -1.126061     0.579560
  O     -0.807429    -0.927499     1.637689
  O     -0.801740    -1.740906    -0.440964
  H      0.138327    -1.940271    -0.221798
 ---------------------------------------------------

Cartesian coordinates of :    ABW10_ 8

 ---------------------------------------------------
 Atomic number (AN) and Cartesian coordinates
  AN         X            Y            Z
 ---------------------------------------------------
  O     -1.010541    -3.346389     0.647635
  H     -1.868922    -2.998006     0.366539
  H     -0.372036    -2.821101     0.131196
  O      1.626480     2.203815     0.705664
  H      0.913696     2.804459     0.966672
  H      1.466936     1.377537     1.195624
  O     -0.297956    -2.083634     3.061022
  H     -0.587674    -2.568165     2.262969
  H      0.146342    -2.737837     3.606054
  O      4.251859     1.466257    -0.202338
  H      4.026991     1.189485    -1.106837
  H      3.440238     1.879292     0.126992
  O      4.112331    -1.020486     1.039844
  H      4.252064    -0.127562     0.662237
  H      4.899594    -1.217913     1.552768
  O      0.711320     1.065868    -1.772407
  H      0.556836     0.153438    -1.473145
  H      0.972872     1.532325    -0.959425
  O      3.276152     0.483751    -2.681954
  H      2.351120     0.738904    -2.489655
  H      3.469743     0.811765    -3.563169
  O      0.711414    -1.550709    -0.640617
  H      1.576240    -1.837737    -0.989741
  H      0.931439    -1.107181     0.200353
  O      3.352346    -2.099421    -1.506607
  H      3.402677    -1.276916    -2.021169
  H      3.714313    -1.857218    -0.638715
  O      1.487791    -0.354381     1.813247
  H      0.936257    -0.959954     2.344501
  H      2.385200    -0.722452     1.771538
  C     -2.803169     0.158063    -0.365606
  C     -2.870517     1.415830    -0.963958
  C     -2.309632     2.513128    -0.356377
  C     -1.655758     2.384290     0.871670
```

```
  C     -1.588202      1.123485      1.473098
  C     -2.156646      0.033922      0.863312
  H     -3.369718      1.533760     -1.911528
  H     -2.366023      3.484001     -0.824394
  H     -1.084760      1.015398      2.421915
  H     -2.108984     -0.929741      1.342525
  N     -1.030681      3.459190      1.450832
  H     -1.315252      4.367924      1.127585
  H     -0.891499      3.406751      2.445918
  C     -3.397266     -1.024212     -0.977580
  O     -3.382696     -2.144614     -0.496726
  O     -3.991454     -0.788886     -2.152941
  H     -4.352150     -1.623978     -2.481361
 ---------------------------------------------------------

Cartesian coordinates of :    ABW10_ 9

 ---------------------------------------------------------
 Atomic number (AN) and Cartesian coordinates
  AN         X            Y             Z
 ---------------------------------------------------------
  O      1.389452     -1.954663     -0.819708
  H      0.863251     -2.648052     -0.378907
  H      2.278498     -1.978117     -0.420212
  O      0.889829      2.338982     -0.190024
  H      0.651632      1.560481      0.347267
  H      0.057200      2.809242     -0.334952
  O     -0.309078     -3.773247      0.481616
  H     -0.511104     -3.236847      1.269555
  H     -1.038576     -3.586305     -0.132188
  O      3.666620      2.342212      0.501591
  H      3.978942      1.787700     -0.233942
  H      2.742523      2.530811      0.277821
  O     -0.809017     -2.052991      2.686643
  H     -0.207087     -2.319549      3.386073
  H     -0.361452     -1.309942      2.239881
  O      1.588729      0.497563     -2.273789
  H      1.355198     -0.340391     -1.844082
  H      1.295339      1.181472     -1.647418
  O      4.271143      0.506077     -1.574630
  H      3.366071      0.506990     -1.948548
  H      4.870447      0.636799     -2.313041
  O      3.977261     -1.634973      0.261248
  H      3.756072     -1.101243      1.040769
  H      4.210679     -0.973702     -0.411639
  O      3.135212      0.251118      2.248089
  H      3.396493      0.412395      3.157610
  H      3.385861      1.050226      1.740498
  O      0.558986     -0.088360      1.210041
  H      0.770420     -0.669006      0.456747
  H      1.406541      0.006899      1.683832
  C     -2.530121      1.136234     -0.189020
  C     -3.210523      0.177125      0.559711
  C     -3.202739     -1.142962      0.179360
```

```
  C     -2.514999     -1.544571     -0.968503
  C     -1.823169     -0.585786     -1.713713
  C     -1.837340      0.732018     -1.328950
  H     -3.747041      0.469769      1.447198
  H     -3.731100     -1.881656      0.762359
  H     -1.284485     -0.890856     -2.598040
  H     -1.311500      1.467167     -1.916501
  N     -2.481553     -2.870288     -1.330018
  H     -3.249473     -3.430898     -0.999821
  H     -2.235822     -3.055363     -2.288094
  C     -2.511210      2.543452      0.191879
  O     -1.826005      3.399775     -0.342648
  O     -3.324402      2.845227      1.208794
  H     -3.225884      3.787169      1.405528
 ---------------------------------------------------

Cartesian coordinates of :    ABW10_10

 ---------------------------------------------------
 Atomic number (AN) and Cartesian coordinates
  AN          X             Y             Z
 ---------------------------------------------------
  O     -0.109367     -2.215300      0.048699
  H      0.130885     -1.813303     -0.803045
  H      0.728937     -2.573139      0.387809
  O      1.017350     -0.996951     -2.274401
  H      1.879821     -1.427576     -2.091677
  H      0.775786     -1.257081     -3.166791
  O     -0.139963     -0.075038      1.780452
  H     -1.020193      0.071337      2.136492
  H     -0.224559     -0.816039      1.143031
  O      3.766143      1.512238      1.593410
  H      4.123996      1.183661      0.752007
  H      2.927073      1.929499      1.348703
  O      2.459715     -0.712444      2.759942
  H      1.535346     -0.475853      2.582874
  H      2.973871      0.023591      2.379594
  O      2.047577      1.594186     -1.740261
  H      1.607300      0.761208     -1.981895
  H      1.708163      1.809662     -0.852948
  O      4.473965      0.528893     -0.989675
  H      3.671286      0.967896     -1.344242
  H      5.215319      0.899987     -1.474423
  O      2.474295     -2.860348      1.029730
  H      2.753558     -3.660664      1.480322
  H      2.551182     -2.134548      1.684688
  O      3.443300     -2.066677     -1.491155
  H      3.905583     -1.226781     -1.329906
  H      3.183213     -2.383041     -0.608196
  O      1.085950      2.254193      0.853137
  H      0.605186      1.455847      1.143901
  H      0.398473      2.864989      0.546251
  C     -3.138244     -0.186859     -0.130045
  C     -2.264005      0.087095     -1.180240
```

C	-1.673708	1.322309	-1.297268
C	-1.945622	2.326696	-0.364529
C	-2.828481	2.055972	0.684925
C	-3.412114	0.817609	0.795915
H	-2.040887	-0.678020	-1.905412
H	-0.993186	1.527694	-2.109215
H	-3.043271	2.829157	1.406677
H	-4.090828	0.613753	1.608764
N	-1.298505	3.535515	-0.436974
H	-0.934554	3.788294	-1.340177
H	-1.729401	4.299610	0.055203
C	-3.761318	-1.496216	0.040610
O	-4.489588	-1.802208	0.965112
O	-3.462265	-2.370784	-0.929862
H	-3.904501	-3.206458	-0.726799

Cartesian coordinates of :   ABW10_11

Atomic number (AN) and Cartesian coordinates

AN	X	Y	Z
O	1.828322	1.106201	-2.012406
H	1.701175	1.555112	-1.160291
H	2.744084	0.784108	-1.976214
O	-1.606825	-1.894418	2.345127
H	-2.489342	-1.546344	2.189110
H	-1.424538	-2.445417	1.559026
O	3.318030	-2.438921	-0.552082
H	3.804165	-1.697489	-0.949379
H	3.241193	-2.192004	0.384010
O	4.202164	1.046515	1.057382
H	3.414519	1.601577	0.956817
H	3.866617	0.243533	1.491689
O	-0.914828	-3.310019	0.037098
H	-0.223780	-3.927694	0.290830
H	-0.456108	-2.628292	-0.478887
O	0.473140	-0.285759	1.291409
H	-0.298215	-0.679063	1.737045
H	0.475955	-0.680436	0.400362
O	2.946540	-1.304253	2.032487
H	2.979612	-1.662674	2.922470
H	2.031872	-0.984851	1.900109
O	1.600285	2.204071	0.670484
H	0.967980	2.935002	0.710502
H	1.103960	1.423550	0.975083
O	4.411465	0.012592	-1.501874
H	4.452973	0.420403	-0.612031
H	5.252692	0.203217	-1.923438
O	0.789418	-1.430637	-1.307350
H	1.023326	-0.546601	-1.646556
H	1.648408	-1.840586	-1.089127
C	-2.658615	0.253692	-0.419126

C	-1.843299	0.876924	-1.363210
C	-1.292724	2.108776	-1.108428
C	-1.531196	2.754228	0.108043
C	-2.356018	2.135383	1.052041
C	-2.911084	0.908376	0.785011
H	-1.637978	0.389629	-2.302138
H	-0.657964	2.584682	-1.839810
H	-2.550659	2.632719	1.990006
H	-3.552135	0.439467	1.515054
N	-0.902792	3.938741	0.393480
H	-0.610208	4.488385	-0.396594
H	-1.288706	4.480067	1.147787
C	-3.247645	-1.061346	-0.648385
O	-3.951887	-1.655771	0.145837
O	-2.945844	-1.588892	-1.842347
H	-3.355699	-2.463053	-1.895603

Cartesian coordinates of : ABW10_12

Atomic number (AN) and Cartesian coordinates

AN	X	Y	Z
O	-1.437536	-2.300706	-1.733019
H	-0.997659	-3.015162	-2.200786
H	-0.744167	-1.629418	-1.587001
O	2.112349	1.100439	1.888075
H	1.865860	1.590911	2.676014
H	1.525979	0.325731	1.874000
O	-1.492493	-2.835409	1.044918
H	-1.481638	-2.733555	0.075140
H	-2.295151	-2.369681	1.309865
O	4.523232	-0.107938	1.138543
H	4.549854	0.204596	0.218821
H	3.761386	0.362672	1.520284
O	3.210768	-2.524082	0.794526
H	3.755436	-1.730557	0.980964
H	3.684724	-3.268398	1.172902
O	1.752974	2.040700	-1.504553
H	1.276381	1.202511	-1.355307
H	1.445701	2.614009	-0.788850
O	4.220772	0.775399	-1.545165
H	3.418837	1.334371	-1.524728
H	4.841209	1.216551	-2.129946
O	0.586298	-0.478605	-1.061272
H	1.373000	-0.952943	-1.399754
H	0.603873	-0.638116	-0.103420
O	2.941703	-1.737019	-1.926992
H	3.468320	-0.920295	-1.900383
H	3.102414	-2.153908	-1.064427
O	0.791669	-1.387877	1.737363
H	-0.017619	-1.899272	1.543257
H	1.545024	-1.939487	1.468454

C	-2.836838	0.509589	0.088349
C	-2.612116	1.422500	-0.942284
C	-1.693735	2.432845	-0.796061
C	-0.966034	2.563542	0.391636
C	-1.188135	1.646027	1.423368
C	-2.107368	0.638187	1.268913
H	-3.166174	1.339573	-1.862716
H	-1.523105	3.135331	-1.597444
H	-0.634899	1.744102	2.344297
H	-2.276455	-0.060953	2.072678
N	-0.012003	3.538017	0.515562
H	-0.122925	4.352858	-0.063077
H	0.313686	3.730805	1.446916
C	-3.825657	-0.556108	-0.020953
O	-4.065607	-1.374888	0.849419
O	-4.481893	-0.574404	-1.187166
H	-5.106471	-1.312754	-1.169916

Cartesian coordinates of : ABW10_13

Atomic number (AN) and Cartesian coordinates

AN	X	Y	Z
O	4.597462	0.805857	-0.627753
H	4.059332	1.525784	-0.236687
H	5.411614	1.208739	-0.937506
O	0.380033	0.068246	1.515459
H	0.345743	0.707387	0.779451
H	1.131449	0.356851	2.059302
O	2.923713	2.611788	0.620657
H	2.065939	2.444747	0.188527
H	2.885439	2.103345	1.447164
O	-1.754415	3.551987	-0.872930
H	-1.772442	4.174436	-0.141322
H	-2.494880	2.948389	-0.703549
O	0.488742	1.933564	-0.577241
H	0.602472	1.498668	-1.444309
H	-0.291578	2.512731	-0.667562
O	1.704612	-2.104051	0.407147
H	1.058920	-2.754380	0.091364
H	1.174101	-1.371386	0.782626
O	2.855040	-0.996621	-1.924048
H	3.498197	-0.363701	-1.562653
H	2.442592	-1.398243	-1.137602
O	2.809652	0.785456	2.796416
H	3.041151	0.859155	3.724939
H	3.368637	0.074143	2.425208
O	0.904273	0.625132	-3.001128
H	1.612292	0.033131	-2.669559
H	1.345797	1.240344	-3.591758
O	4.240290	-1.154743	1.391782
H	4.478098	-0.535452	0.680805

```
  H      3.471823     -1.634393      1.049654
  C     -3.015398     -0.308355      0.158441
  C     -2.968771     -1.398858      1.026621
  C     -2.198393     -2.496804      0.732872
  C     -1.449338     -2.538764     -0.445883
  C     -1.488966     -1.443562     -1.314210
  C     -2.260871     -0.348633     -1.013347
  H     -3.545461     -1.384764      1.936615
  H     -2.166680     -3.338195      1.408009
  H     -0.908833     -1.471291     -2.223551
  H     -2.289766      0.490177     -1.691083
  N     -0.632087     -3.608049     -0.713372
  H     -0.852015     -4.464917     -0.234798
  H     -0.370933     -3.735323     -1.676582
  C     -3.840518      0.859255      0.436251
  O     -3.930448      1.839748     -0.284261
  O     -4.531646      0.781967      1.577982
  H     -5.041335      1.598051      1.677338
 ---------------------------------------------------

Cartesian coordinates of :    ABW10_14

 ---------------------------------------------------
 Atomic number (AN) and Cartesian coordinates
  AN          X             Y             Z
 ---------------------------------------------------
  O      0.623381      2.723902     -1.514849
  H      0.339413      1.904231     -1.069474
  H      0.872671      3.321160     -0.804912
  O     -1.004958     -2.197882     -1.342270
  H     -0.738692     -1.283832     -1.126075
  H     -0.215312     -2.613075     -1.710684
  O     -1.863855      3.208214     -2.734377
  H     -0.974099      3.102710     -2.348009
  H     -1.814024      2.781923     -3.593501
  O     -3.818420     -1.307574      1.502083
  H     -4.451733     -1.844671      1.984340
  H     -2.957702     -1.768041      1.583099
  O     -2.961370      1.359069      2.027100
  H     -3.339501      0.464057      2.005569
  H     -2.956387      1.612128      1.091395
  O     -1.270801     -2.392513      1.553519
  H     -1.112103     -2.436286      0.596902
  H     -0.830199     -1.577533      1.842940
  O     -3.800647     -1.137814     -1.342187
  H     -2.954276     -1.556970     -1.543155
  H     -3.901870     -1.258531     -0.382121
  O     -0.550232      0.404447     -0.443699
  H     -1.440713      0.796787     -0.507329
  H     -0.397265      0.282212      0.507139
  O     -3.111548      1.572002     -0.836530
  H     -3.412140      0.715230     -1.186103
  H     -2.774140      2.097287     -1.585544
  O     -0.445769      0.185618      2.479434
```

H	-1.220915	0.772021	2.416566
H	0.345638	0.736460	2.552513
C	2.624817	0.211733	0.496972
C	2.920341	0.220147	-0.865679
C	2.737921	-0.906847	-1.629202
C	2.268000	-2.088352	-1.046784
C	2.026092	-2.112677	0.331464
C	2.192492	-0.977678	1.082756
H	3.263543	1.127107	-1.335155
H	2.943012	-0.886792	-2.688668
H	1.684672	-3.027978	0.789957
H	1.980922	-0.998793	2.139776
N	1.979572	-3.180609	-1.816662
H	2.393722	-3.214451	-2.731817
H	1.933288	-4.067777	-1.346113
C	2.664791	1.424110	1.302124
O	2.166327	1.537740	2.410494
O	3.292760	2.450730	0.719625
H	3.231513	3.215562	1.308445

Cartesian coordinates of : ABW10_15

Atomic number (AN) and Cartesian coordinates

AN	X	Y	Z
O	3.836594	-1.858733	-1.920882
H	2.961950	-1.439006	-1.894252
H	3.954921	-2.152681	-1.002867
O	5.442799	-0.020275	1.003084
H	5.477278	0.222615	0.063761
H	4.904283	-0.830803	1.024909
O	5.103174	0.616093	-1.772281
H	4.739008	-0.276113	-1.940641
H	5.759488	0.773014	-2.455038
O	3.668460	1.912287	1.815860
H	4.353497	1.241295	1.608429
H	4.129811	2.667371	2.187760
O	2.790684	2.001093	-0.885576
H	3.585129	1.659585	-1.329965
H	3.052431	2.066378	0.049846
O	3.787661	-2.309026	0.907701
H	2.862450	-2.108424	1.138200
H	4.016930	-3.101629	1.398809
O	0.986229	1.155537	2.178631
H	1.919142	1.426299	2.147554
H	0.576935	1.618836	1.428031
O	1.155972	-1.529234	1.511754
H	0.300373	-1.746727	1.112120
H	1.072686	-0.595257	1.795316
O	-0.048610	2.543312	-0.119330
H	-0.608428	1.857523	-0.502193
H	0.791834	2.458289	-0.587661

```
  O      1.451053     -0.451436     -1.179148
  H      1.515655     -0.872426     -0.306259
  H      1.872053      0.424367     -1.062402
  C     -3.266407     -0.452833     -0.285954
  C     -3.809533      0.681970     -0.889450
  C     -5.132663      1.005173     -0.719856
  C     -5.968456      0.197987      0.063785
  C     -5.423245     -0.944221      0.666788
  C     -4.099114     -1.256062      0.493161
  H     -3.183704      1.316687     -1.495537
  H     -5.543828      1.885829     -1.189457
  H     -6.059888     -1.574616      1.268902
  H     -3.690032     -2.137282      0.961539
  N     -7.267450      0.536630      0.267734
  H     -7.681437      1.211809     -0.348480
  H     -7.887340     -0.164588      0.629714
  C     -1.862939     -0.814960     -0.437950
  O     -1.344701     -1.776805      0.114289
  O     -1.171642     -0.011155     -1.238171
  H     -0.206282     -0.262360     -1.244868
 ---------------------------------------------------

Cartesian coordinates of :    ABW10_16

 ---------------------------------------------------
 Atomic number (AN) and Cartesian coordinates
  AN          X             Y             Z
 ---------------------------------------------------
  O      0.935206     -1.276308      2.516228
  H      1.057787     -1.993384      1.861437
  H      1.758522     -0.779481      2.501088
  O     -3.214498      0.082200     -1.985520
  H     -3.034458      0.811033     -1.368358
  H     -2.355738     -0.354667     -2.098592
  O     -1.743903     -3.458853     -0.229247
  H     -2.147927     -4.308000     -0.422766
  H     -2.464613     -2.881002      0.079008
  O     -0.668984     -1.320872     -1.848413
  H     -0.578373     -0.836832     -1.008814
  H     -1.051941     -2.164676     -1.559590
  O     -3.724244     -1.565232      0.328157
  H     -3.649068     -1.051308     -0.494110
  H     -3.945068     -0.906221      1.009601
  O     -2.635819      1.907529      0.151125
  H     -2.152180      2.698581     -0.132655
  H     -1.942077      1.281911      0.429047
  O     -4.255865      0.598024      2.110339
  H     -3.817150      1.227368      1.517385
  H     -5.193094      0.792933      2.030309
  O      1.961191     -2.419821     -1.977707
  H      1.115138     -1.945887     -2.046440
  H      2.585721     -1.771662     -1.621359
  O     -0.969762     -0.232381      0.767741
  H     -1.704217     -0.847836      0.883082
```

H	-0.300845	-0.484178	1.433660
O	0.976040	-3.276264	0.597702
H	1.397432	-3.016998	-0.238887
H	0.032249	-3.347800	0.378271
C	2.309249	1.000349	0.048925
C	1.848078	1.851209	1.054202
C	0.909832	2.815021	0.780920
C	0.396407	2.956898	-0.511914
C	0.843808	2.093102	-1.516193
C	1.789924	1.137330	-1.237156
H	2.233456	1.756044	2.056094
H	0.560152	3.472506	1.562006
H	0.445229	2.191868	-2.514345
H	2.138648	0.485071	-2.022014
N	-0.581528	3.884242	-0.769774
H	-0.628038	4.665248	-0.137325
H	-0.711536	4.135005	-1.735171
C	3.323958	-0.013028	0.306361
O	3.786350	-0.771338	-0.528245
O	3.740598	-0.055216	1.577804
H	4.404669	-0.754345	1.657275

Cartesian coordinates of : ABW10_17

Atomic number (AN) and Cartesian coordinates

AN	X	Y	Z
O	2.192853	-1.623683	2.391119
H	1.377378	-1.127614	2.210929
H	2.321188	-2.163437	1.592144
O	0.607617	2.258630	0.360446
H	0.307749	1.440019	0.801191
H	-0.126948	2.878898	0.450091
O	-0.090417	-0.209971	1.444218
H	-0.096716	-0.747707	0.622480
H	-0.985156	-0.256723	1.792750
O	3.438056	2.284766	0.241769
H	3.712256	1.580787	0.854789
H	2.474586	2.350453	0.343004
O	4.790747	-1.222862	-0.650906
H	4.461263	-0.546584	-1.266625
H	4.757009	-0.786010	0.217224
O	0.772462	0.838091	-2.166160
H	1.738080	0.839336	-2.277631
H	0.626426	1.387477	-1.378718
O	3.616618	0.898602	-2.134698
H	3.604053	1.466718	-1.334165
H	4.109580	1.388983	-2.797178
O	0.161311	-1.620585	-0.911939
H	1.011452	-2.058019	-0.733239
H	0.383954	-0.823249	-1.427949
O	2.605011	-2.828522	-0.142093

H	2.794792	-3.750434	-0.330878
H	3.407570	-2.325266	-0.395168
O	4.274828	0.093196	1.812589
H	3.537188	-0.482693	2.105210
H	4.866153	0.174231	2.564527
C	-3.282027	-0.510428	-0.106519
C	-2.679675	0.196075	-1.146981
C	-2.375928	1.527207	-1.005648
C	-2.653958	2.195798	0.190582
C	-3.272108	1.492525	1.229575
C	-3.581889	0.162829	1.076464
H	-2.438953	-0.306581	-2.069317
H	-1.898691	2.065994	-1.809775
H	-3.498436	2.005144	2.152142
H	-4.055350	-0.374878	1.882582
N	-2.256968	3.495184	0.360567
H	-2.130695	4.037993	-0.476537
H	-2.680145	4.000450	1.119634
C	-3.584678	-1.933967	-0.208492
O	-4.102723	-2.599467	0.667974
O	-3.232850	-2.480167	-1.381362
H	-3.467327	-3.418033	-1.357022

Cartesian coordinates of :   ABW10_18

Atomic number (AN) and Cartesian coordinates

AN	X	Y	Z
O	-4.067564	0.809490	-1.888023
H	-4.392861	-0.026419	-1.492524
H	-4.720005	1.074632	-2.540000
O	-2.083689	-1.928346	2.119933
H	-1.276142	-1.418357	1.944160
H	-2.805583	-1.276178	2.115482
O	-4.856509	-1.468248	-0.547534
H	-4.080654	-2.052307	-0.597868
H	-4.787691	-1.064897	0.334704
O	-2.420146	-2.922331	-0.437198
H	-2.397719	-3.881727	-0.474113
H	-2.293905	-2.684064	0.505808
O	0.189259	-0.460292	1.245836
H	0.191344	-0.818176	0.332414
H	1.099842	-0.503668	1.550195
O	-0.773172	2.103849	0.711452
H	-0.028474	2.715419	0.608087
H	-0.376152	1.248430	0.963204
O	-1.262716	1.114893	-1.974540
H	-2.228082	1.010124	-2.014245
H	-1.093073	1.517551	-1.107193
O	-4.265560	-0.146086	1.893411
H	-4.869985	0.036334	2.616589
H	-4.038796	0.722825	1.499559

O	-0.187566	-1.401408	-1.328531
H	-0.579715	-0.560343	-1.631601
H	-0.944222	-1.973350	-1.115436
O	-3.591097	2.172653	0.559012
H	-3.780105	1.817201	-0.325912
H	-2.622279	2.214879	0.610969
C	3.490193	-0.338685	-0.131426
C	3.665177	0.422728	1.023380
C	3.140227	1.690135	1.113158
C	2.427755	2.237887	0.043540
C	2.257328	1.477556	-1.117025
C	2.781900	0.211359	-1.197792
H	4.213522	0.016430	1.857241
H	3.275521	2.273811	2.010814
H	1.707908	1.896104	-1.946266
H	2.641452	-0.371772	-2.093605
N	1.838765	3.473977	0.155474
H	2.211884	4.077556	0.868365
H	1.641981	3.946947	-0.710560
C	4.011633	-1.697198	-0.255231
O	3.873274	-2.407417	-1.231937
O	4.675496	-2.123065	0.829327
H	4.975263	-3.027071	0.662130

Cartesian coordinates of : ABW10_19

Atomic number (AN) and Cartesian coordinates

AN	X	Y	Z
O	-3.920938	0.033164	2.090891
H	-3.178165	0.652355	2.028255
H	-4.332233	0.044949	1.211985
O	-1.424023	1.506147	2.009422
H	-0.942819	2.213740	1.553443
H	-0.811930	0.753796	2.014659
O	-2.389642	-2.236209	1.616500
H	-2.464595	-2.888444	2.317832
H	-2.975250	-1.494101	1.873228
O	1.138521	-2.898370	-0.535213
H	0.971777	-2.386054	0.268037
H	2.024341	-2.628026	-0.812782
O	-3.292277	-2.676966	-1.034264
H	-2.998875	-2.674132	-0.107205
H	-3.895477	-1.919668	-1.077817
O	-2.142478	0.863341	-0.831598
H	-1.890770	1.005511	0.092714
H	-1.593608	0.119727	-1.149527
O	-1.610426	3.260563	-2.247560
H	-1.783624	2.412547	-1.801484
H	-0.924580	3.060358	-2.889421
O	-4.694851	-0.179297	-0.668283
H	-5.351726	0.324757	-1.154357

H	-3.839490	0.265923	-0.830090
O	-0.910920	-1.397603	-1.920536
H	-0.172446	-1.860763	-1.491030
H	-1.702755	-1.915892	-1.687237
O	0.061995	-0.936635	1.432235
H	-0.777463	-1.433482	1.500082
H	0.031421	-0.525256	0.561717
C	3.077644	0.118309	-0.058756
C	3.124492	1.008094	1.014359
C	2.231158	2.046736	1.103307
C	1.263910	2.239239	0.110748
C	1.228780	1.357405	-0.976883
C	2.118537	0.314218	-1.051405
H	3.860753	0.874391	1.789832
H	2.264117	2.723888	1.943169
H	0.498165	1.505900	-1.756046
H	2.079542	-0.363307	-1.889926
N	0.335696	3.235242	0.234391
H	0.584238	4.015854	0.817138
H	-0.201695	3.465270	-0.588284
C	3.989439	-1.012455	-0.169042
O	3.890406	-1.905031	-0.994985
O	4.976887	-1.010677	0.732439
H	5.511490	-1.805236	0.596019

Cartesian coordinates of : ABW10_20

Atomic number (AN) and Cartesian coordinates

AN	X	Y	Z
O	-1.164463	2.817864	0.935154
H	-2.045334	2.482354	0.712661
H	-0.613484	2.027910	1.067298
O	2.398041	-1.330355	-1.867530
H	2.104668	-1.665232	-1.003610
H	1.994746	-0.450035	-1.920109
O	-0.436015	3.404130	-1.705374
H	-0.695227	3.281853	-0.770919
H	0.046637	4.234324	-1.725963
O	4.854388	-0.480551	-0.700726
H	4.717663	-0.834112	0.193233
H	4.080749	-0.803433	-1.196776
O	3.882814	2.082970	-0.332527
H	4.327742	1.226124	-0.502448
H	4.507826	2.764005	-0.592083
O	1.511298	-2.084930	0.779636
H	1.057567	-1.232714	0.917411
H	0.837392	-2.706246	0.470904
O	4.011061	-1.421661	1.861355
H	3.183333	-1.842364	1.575408
H	4.419414	-2.027818	2.483977
O	0.661479	0.592702	1.080709

```
  H      1.459294      0.863194      1.574846
  H      0.878639      0.792368      0.150846
  O      3.124700      1.255335      2.294215
  H      3.479939      0.351873      2.260524
  H      3.454031      1.670514      1.480462
  O      1.447399      1.345555     -1.558778
  H      0.813929      2.075900     -1.692862
  H      2.264995      1.751705     -1.223342
  C     -3.222148     -0.323624      0.025883
  C     -3.480163     -1.618081      0.476828
  C     -2.638518     -2.654049      0.154620
  C     -1.513008     -2.427928     -0.642971
  C     -1.269454     -1.136069     -1.119681
  C     -2.106246     -0.102592     -0.780713
  H     -4.341832     -1.808999      1.095014
  H     -2.835277     -3.650846      0.518641
  H     -0.408607     -0.959497     -1.745557
  H     -1.902451      0.892280     -1.145311
  N     -0.622473     -3.437935     -0.896843
  H     -0.962057     -4.377374     -0.783129
  H     -0.019733     -3.310346     -1.692602
  C     -4.077854      0.800097      0.382630
  O     -3.819986      1.971008      0.155882
  O     -5.209233      0.452155      1.004108
  H     -5.694768      1.259844      1.222807
 ---------------------------------------------------

Cartesian coordinates of :    ABW10_21

 ---------------------------------------------------
 Atomic number (AN) and Cartesian coordinates
  AN         X             Y             Z
 ---------------------------------------------------
  O      2.970451     -0.063696      1.865643
  H      3.422632     -0.671914      1.253425
  H      2.109065     -0.473742      2.034617
  O      1.084685     -0.216289     -1.300102
  H      1.323972     -0.989746     -0.765868
  H      1.601150      0.523060     -0.945075
  O      4.147582     -1.713038     -0.141843
  H      3.289276     -2.151334     -0.241354
  H      4.186687     -1.061793     -0.863285
  O      2.882818      1.814427     -0.172614
  H      2.888910      1.242899      0.619970
  H      2.295228      2.555343      0.048836
  O      0.582729     -1.518042      2.567308
  H     -0.309790     -1.808456      2.283370
  H      0.812774     -2.082459      3.309589
  O     -1.018977     -3.276122     -0.989670
  H     -1.300784     -4.086258     -1.420710
  H     -1.161453     -2.554869     -1.634406
  O      4.253596      0.338397     -2.144637
  H      3.798485      0.959842     -1.552528
  H      3.567598      0.043742     -2.749770
```

```
  O      1.509045    -2.732300     0.111542
  H      0.677175    -3.028478    -0.297182
  H      1.258378    -2.388246     0.984135
  O     -1.211800    -1.057431    -2.654228
  H     -0.500971    -0.582619    -2.188715
  H     -2.017060    -0.566444    -2.467629
  O     -1.795983    -2.522275     1.617884
  H     -2.478109    -1.851597     1.472679
  H     -1.566394    -2.828754     0.723301
  C     -2.290686     1.057009     0.088842
  C     -1.933790     1.968371    -0.905138
  C     -0.912082     2.862240    -0.701086
  C     -0.207899     2.866760     0.505686
  C     -0.542176     1.932727     1.490695
  C     -1.576150     1.052452     1.285325
  H     -2.469638     1.980262    -1.840027
  H     -0.647763     3.573459    -1.468502
  H      0.010500     1.919414     2.417763
  H     -1.844331     0.351656     2.059403
  N      0.838167     3.736705     0.694026
  H      0.834119     4.560745     0.116600
  H      1.092937     3.916757     1.650770
  C     -3.396815     0.124051    -0.084930
  O     -3.802291    -0.647557     0.767085
  O     -3.964195     0.173216    -1.295594
  H     -4.683450    -0.473388    -1.320156
 --------------------------------------------------

Cartesian coordinates of :    ABW10_22

 --------------------------------------------------
 Atomic number (AN) and Cartesian coordinates
  AN          X            Y            Z
 --------------------------------------------------
  O     -1.293852    -1.167394    -1.541171
  H     -0.416147    -1.481530    -1.774992
  H     -1.342237    -1.239446    -0.570309
  O     -0.208223     3.568066    -1.042280
  H     -0.611694     4.423513    -1.209428
  H     -0.202665     3.471585    -0.072678
  O     -3.887747    -2.158933    -1.859259
  H     -2.938331    -1.954350    -1.920725
  H     -4.084386    -2.753470    -2.586921
  O     -1.640073    -1.293206     1.296310
  H     -2.476311    -1.795637     1.243749
  H     -1.904624    -0.357541     1.383507
  O     -4.795901     0.511173    -1.449392
  H     -3.954246     0.989210    -1.488734
  H     -4.565765    -0.403437    -1.683949
  O     -4.170586    -2.450340     0.959860
  H     -4.141097    -2.473411    -0.010803
  H     -4.626756    -1.617652     1.162970
  O     -2.105991     1.556807    -1.449285
  H     -1.421848     2.250657    -1.394856
```

H	-1.644292	0.711196	-1.567751
O	-0.172644	2.904327	1.720479
H	-1.067158	2.536938	1.777454
H	0.358052	2.140993	1.454063
O	-2.564902	1.364893	1.331320
H	-3.497646	1.133983	1.469477
H	-2.468355	1.456976	0.364222
O	-5.171231	0.221941	1.271970
H	-5.144678	0.364510	0.302552
H	-6.029663	0.538681	1.562794
C	2.941559	-0.421840	0.265656
C	3.740363	-1.548446	0.462679
C	5.085459	-1.514855	0.190645
C	5.685516	-0.343778	-0.291209
C	4.881514	0.786659	-0.493632
C	3.538675	0.742173	-0.218163
H	3.298333	-2.459338	0.832188
H	5.695571	-2.391721	0.345803
H	5.333115	1.691986	-0.869923
H	2.929159	1.617369	-0.379948
N	7.023798	-0.292367	-0.518717
H	7.521193	-1.160582	-0.596968
H	7.376680	0.464087	-1.076011
C	1.511550	-0.423574	0.547330
O	0.785410	0.548650	0.394088
O	1.036483	-1.582818	0.993280
H	0.065464	-1.500359	1.149033

Cartesian coordinates of :   ABW10_23

Atomic number (AN) and Cartesian coordinates

AN	X	Y	Z
O	2.651874	3.947163	-0.679708
H	1.729988	3.712289	-0.889052
H	2.592974	4.554357	0.062106
O	0.726746	-3.147439	0.095594
H	-0.170595	-3.440130	-0.124468
H	1.060353	-2.713460	-0.712256
O	0.137172	2.927545	-1.446913
H	0.401977	2.044858	-1.127225
H	-0.763677	3.036131	-1.112393
O	3.284416	-2.060751	1.266931
H	3.027183	-1.320989	1.845258
H	2.455299	-2.492346	1.030454
O	4.406153	-0.751871	-0.921690
H	5.327484	-1.006213	-1.013756
H	4.070822	-1.256827	-0.153984
O	0.004742	-0.925766	1.697113
H	-0.954367	-0.856120	1.711806
H	0.201794	-1.740960	1.196963
O	2.441642	0.227059	2.747103

```
  H      2.783965      0.802136      2.043957
  H      1.537456      0.010511      2.479193
  O      1.113183      0.524356     -0.449317
  H      1.946758      0.844147     -0.066333
  H      0.632635      0.113026      0.291313
  O      3.607729      1.594854      0.535015
  H      4.039184      0.980971     -0.079773
  H      3.379447      2.404733      0.042277
  O      1.800720     -1.654746     -2.052512
  H      2.758165     -1.627750     -1.928860
  H      1.500809     -0.853702     -1.582442
  C     -2.857000      0.575748      0.001510
  C     -3.396890     -0.414904      0.822752
  C     -3.187006     -1.745543      0.548422
  C     -2.426102     -2.127823     -0.560956
  C     -1.892356     -1.135675     -1.389013
  C     -2.109495      0.191040     -1.109865
  H     -3.984173     -0.138750      1.682855
  H     -3.605686     -2.506577      1.188823
  H     -1.307260     -1.424099     -2.249090
  H     -1.694656      0.948098     -1.755303
  N     -2.152662     -3.450531     -0.799222
  H     -2.760428     -4.116906     -0.354025
  H     -1.918546     -3.687817     -1.748798
  C     -3.051719      1.996015      0.273067
  O     -2.574446      2.906031     -0.382037
  O     -3.825231      2.244607      1.335485
  H     -3.892457      3.203637      1.441298
 ---------------------------------------------------

Cartesian coordinates of :    ABW10_24

 ---------------------------------------------------
 Atomic number (AN) and Cartesian coordinates
  AN          X             Y             Z
 ---------------------------------------------------
  O      0.061232      3.158162      1.676888
  H      0.640951      3.240608      0.908828
  H      0.347746      2.345831      2.133669
  O     -3.615021     -2.694516     -1.014425
  H     -3.823111     -2.515349     -0.083216
  H     -3.931224     -1.902972     -1.481257
  O     -4.745539      0.768486      0.505725
  H     -4.607315     -0.071111      0.974248
  H     -3.899515      1.235000      0.587771
  O     -3.999945     -1.739889      1.648132
  H     -3.089679     -1.413577      1.782477
  H     -4.271057     -2.134435      2.480333
  O     -2.130856      1.919418      0.369767
  H     -1.379256      2.395243      0.768063
  H     -1.834936      1.593600     -0.499946
  O     -4.326968     -0.150651     -2.081012
  H     -5.033853      0.072289     -2.691095
  H     -4.582140      0.225275     -1.213785
```

O	0.617070	0.725884	3.024796
H	0.255174	0.837319	3.907743
H	-0.042240	0.187822	2.550593
O	-0.965059	-1.879069	-0.851822
H	-1.839505	-2.304217	-0.961299
H	-0.302953	-2.550983	-1.030094
O	-1.451341	-0.593605	1.587315
H	-1.741200	0.260462	1.216922
H	-1.148597	-1.094443	0.806601
O	-1.606293	0.637139	-2.104843
H	-2.547402	0.428597	-2.232381
H	-1.232088	-0.187219	-1.755794
C	3.421427	-0.561344	-0.218655
C	4.033377	0.534345	-0.826496
C	3.305626	1.652081	-1.159142
C	1.934654	1.705915	-0.892776
C	1.321870	0.607287	-0.283589
C	2.054405	-0.503837	0.047421
H	5.088730	0.507039	-1.042833
H	3.785977	2.495350	-1.631508
H	0.267658	0.651577	-0.064627
H	1.570233	-1.342924	0.522795
N	1.203289	2.833190	-1.162787
H	1.609579	3.487994	-1.808354
H	0.213319	2.696789	-1.287229
C	4.161160	-1.764799	0.146297
O	3.672075	-2.749883	0.665659
O	5.470060	-1.707820	-0.142099
H	5.873244	-2.543038	0.131901

Cartesian coordinates of :   ABW10_25

Atomic number (AN) and Cartesian coordinates

AN	X	Y	Z
O	2.290668	1.771138	0.064968
H	1.629610	2.407585	0.397521
H	1.905146	1.347757	-0.719728
O	3.911032	-1.838275	1.694595
H	3.067172	-1.384666	1.858352
H	3.749200	-2.339643	0.879492
O	0.409759	3.477369	1.255385
H	0.146260	2.837177	1.944001
H	0.900337	4.160202	1.719906
O	4.829054	0.518767	0.349179
H	4.018862	1.050932	0.372021
H	4.607758	-0.277916	0.862403
O	1.451443	-0.359854	1.786877
H	1.027395	-0.941565	1.131451
H	1.802787	0.372122	1.246738
O	3.199550	-2.921341	-0.854916
H	3.443822	-3.728739	-1.313226

H	3.538242	-2.180523	-1.399147
O	1.264329	0.249298	-2.173947
H	0.552009	0.874972	-2.360788
H	0.872974	-0.462261	-1.637366
O	4.024316	-0.604402	-2.134979
H	4.378141	-0.182817	-1.330433
H	3.152379	-0.202313	-2.257658
O	-0.270789	1.464095	3.111813
H	0.152204	1.629602	3.958328
H	0.259893	0.758125	2.699458
O	0.648815	-1.899741	-0.443368
H	1.453333	-2.424173	-0.615301
H	-0.096191	-2.498986	-0.532779
C	-3.369891	-0.448465	-0.071051
C	-3.770863	0.244489	-1.213019
C	-2.965052	1.208123	-1.771355
C	-1.726802	1.509606	-1.197897
C	-1.328432	0.819861	-0.049876
C	-2.135236	-0.143298	0.499697
H	-4.723215	0.024030	-1.666650
H	-3.281150	1.738324	-2.656843
H	-0.367525	1.041194	0.385069
H	-1.809797	-0.673968	1.381344
N	-0.875125	2.414274	-1.777203
H	-1.282116	3.058489	-2.432985
H	-0.194379	2.829223	-1.161404
C	-4.196556	-1.481899	0.543728
O	-3.887128	-2.124738	1.528717
O	-5.368368	-1.682669	-0.077970
H	-5.841738	-2.379770	0.396457

Cartesian coordinates of : ABW10_26

Atomic number (AN) and Cartesian coordinates

AN	X	Y	Z
O	3.690699	0.179043	1.911891
H	2.826455	-0.234568	1.782071
H	4.317697	-0.431650	1.488622
O	1.097899	0.355102	-1.314629
H	1.851920	0.907377	-1.041556
H	0.374018	0.976947	-1.492667
O	0.764681	1.334539	2.779147
H	0.805003	0.488512	2.304549
H	1.605105	1.389327	3.242564
O	2.378835	-1.960959	-2.291443
H	2.495211	-2.441332	-1.456058
H	1.786575	-1.226619	-2.063509
O	3.230077	1.941968	-0.246434
H	3.427760	1.390129	0.533560
H	2.524161	2.541562	0.052163
O	2.847012	-3.015939	0.339681

H	2.913016	-3.940109	0.591292
H	3.746662	-2.649850	0.414052
O	4.553025	-0.158547	-1.929180
H	3.829999	-0.776237	-2.143637
H	4.112920	0.607890	-1.539374
O	0.999910	-1.011223	1.150446
H	0.942877	-0.558953	0.288986
H	1.559768	-1.787340	0.983117
O	5.244938	-1.573128	0.353701
H	6.161227	-1.854514	0.402567
H	5.146068	-1.075102	-0.483043
O	0.919963	3.259202	0.739498
H	0.836089	2.622465	1.476202
H	0.980402	4.122570	1.156024
C	-4.045573	-0.347039	0.008986
C	-4.120826	0.195459	-1.273116
C	-3.174003	1.088146	-1.714071
C	-2.117266	1.468914	-0.881813
C	-2.042749	0.926819	0.406237
C	-2.992727	0.035721	0.838439
H	-4.929720	-0.084679	-1.927723
H	-3.237907	1.504473	-2.707858
H	-1.230953	1.218383	1.054159
H	-2.928340	-0.377090	1.832660
N	-1.134859	2.309334	-1.334282
H	-1.354837	2.858717	-2.146820
H	-0.586003	2.788259	-0.632800
C	-5.031740	-1.297624	0.511673
O	-5.014012	-1.796875	1.620631
O	-5.998119	-1.595775	-0.370043
H	-6.601127	-2.225542	0.048297

\---
